# Comparison of normalization and differential expression analyses using RNA-Seq data from 726 individual *Drosophila melanogaster*

**DOI:** 10.1186/s12864-015-2353-z

**Published:** 2016-01-05

**Authors:** Yanzhu Lin, Kseniya Golovnina, Zhen-Xia Chen, Hang Noh Lee, Yazmin L. Serrano Negron, Hina Sultana, Brian Oliver, Susan T. Harbison

**Affiliations:** Laboratory of Systems Genetics, Center for Systems Biology, National Heart Lung and Blood Institute, 10 Center Drive, MSC 1640, Bethesda, MD 20892 USA; Developmental Genomics Section, Laboratory of Cellular and Developmental Biology, National Institute of Diabetes and Digestive and Kidney Diseases, Bethesda, MD USA

**Keywords:** RNA-Seq, Differential expression analysis, *Drosophila melanogaster*

## Abstract

**Background:**

A generally accepted approach to the analysis of RNA-Seq read count data does not yet exist. We sequenced the mRNA of 726 individuals from the *Drosophila* Genetic Reference Panel in order to quantify differences in gene expression among single flies. One of our experimental goals was to identify the optimal analysis approach for the detection of differential gene expression among the factors we varied in the experiment: genotype, environment, sex, and their interactions. Here we evaluate three different filtering strategies, eight normalization methods, and two statistical approaches using our data set. We assessed differential gene expression among factors and performed a statistical power analysis using the eight biological replicates per genotype, environment, and sex in our data set.

**Results:**

We found that the most critical considerations for the analysis of RNA-Seq read count data were the normalization method, underlying data distribution assumption, and numbers of biological replicates, an observation consistent with previous RNA-Seq and microarray analysis comparisons. Some common normalization methods, such as Total Count, Quantile, and RPKM normalization, did not align the data across samples. Furthermore, analyses using the Median, Quantile, and Trimmed Mean of M-values normalization methods were sensitive to the removal of low-expressed genes from the data set. Although it is robust in many types of analysis, the normal data distribution assumption produced results vastly different than the negative binomial distribution. In addition, at least three biological replicates per condition were required in order to have sufficient statistical power to detect expression differences among the three-way interaction of genotype, environment, and sex.

**Conclusions:**

The best analysis approach to our data was to normalize the read counts using the *DESeq* method and apply a generalized linear model assuming a negative binomial distribution using either *edgeR* or *DESeq* software. Genes having very low read counts were removed after normalizing the data and fitting it to the negative binomial distribution. We describe the results of this evaluation and include recommended analysis strategies for RNA-Seq read count data.

**Electronic supplementary material:**

The online version of this article (doi:10.1186/s12864-015-2353-z) contains supplementary material, which is available to authorized users.

## Background

Next-generation transcriptome sequencing promises to reveal the underlying complexities of gene expression. Lagging behind the technology is a generally accepted approach to the analysis of RNA-Seq data, including the experimental design, normalization, and statistical analysis approach [1]. One issue is whether low levels of read counts qualify as a rare transcript, or whether they should be discarded due to the uncertainty in their quantification [2]. Both RNA-Seq and microarrays are problematic for the detection of rare transcripts, although for different reasons [3, 4]. In RNA-Seq technical replicates of the same biological sample may not contain a given transcript if it is rare [2, 5]. Low-expressed transcripts can skew the results when certain types of statistical analyses, such as *t*-statistics, are used [6]. Furthermore, differences in library preparation across samples [2, 6–8], sequencing errors [9, 10], and mapping and annotation errors [9] can all contribute to inaccuracies in read count data. Complicating matters further are well-known biases in RNA-Seq data, such as sequence composition and similarity [5, 7, 10], gene length [6, 7, 9], and sequencing depth [4, 6]. In addition, the best choice of statistical model for differential gene expression is not straightforward. Differences due to technical variation in read count data have been modeled with a Poisson distribution, which assumes that the variance is equal to the mean [6]. However, differences among biological replicates of RNA-Seq read count data are far more variable than would be expected under a Poisson distribution, a phenomenon known as over-dispersion [6, 11, 12]. To account for over-dispersion, a generalized linear model (GLM) using a negative binomial distribution has been proposed [13, 14]. These issues leave the experimenter with several choices to make regarding data analysis: 1) which read counts to include in the analysis and which to discard; 2) which normalization methods will mitigate bias across samples; and 3) the best choice of statistical model to identify differentially expressed genes. Systematic comparisons of these choices as well as other experimental parameters have been made previously with microarray data. Large-scale comparisons using microarrays found that some experimental parameters were more critical than others in the analysis and interpretation of gene expression data. These studies use spike-in RNA standards, similar sample preparation and handling procedures, and/or known mutations as benchmarks to examine potential technical differences in sample preparation and analysis [15–18]. The MAQC Consortium identified and quantified the degree of variability and reproducibility both within and across different microarray platforms [15]. Comparisons of analytical models, normalization methods, and team performance have also been made [16, 19]. The results of differential gene expression analyses were often strongly influenced by the choice of normalization method [17–19]. Differential gene expression was also dependent on the analysis model used [19]; interestingly, differences in model performance could also be traced to the practices and experience of the team doing the analysis [19]. Furthermore, studies of gene expression-phenotype associations revealed that the phenotypic trait itself often had a large effect on the performance of the analysis model [19]. Finally, the distribution of false discovery rates used to control the Type I error rate in multiple testing can be altered by the analysis methods [18]. Comparison studies such as these have provided biologists with the most critical parameters to consider when designing microarray gene expression studies and analyzing the results.

Recently, we generated RNA-Seq data sets for 726 individual *Drosophila melanogaster*. We froze 8 individual flies of each sex from 16 *Drosophila* Genetic Reference Panel (DGRP) genotypes. We replicated this experiment at three different calendar times, maintaining the same environmental conditions for each replicate. Environmental controls included parental culture density; rearing environment including food, temperature, and light: dark cycle; mating status; social exposure; and the circadian time of RNA extraction. Furthermore, we checked for technical sources of variation in our sequence data by adding ERCC spike-in control RNA during library preparation, and prepared duplicate libraries for 118 flies. These procedures enabled us to determine that the differential gene expression we observed was due to largely biological rather than technical sources. In addition, we used the sequence data itself to verify the sex and genotype of each fly. These sex and genotype analyses in combination with other considerations (such as RNA and library preparation failure or low numbers of uniquely mapped reads) led us to exclude the RNA-Seq data for 42 flies, yielding 726 data sets (See Methods for additional detail). We were interested in studying the effects of genotype, environment, and sex on gene expression in individual flies; we were also interested in characterizing the first- and second-order interactions (e.g., Genotype × Environment, Genotype × Sex, Environment × Sex, and Genotype × Environment × Sex). This large dataset is also valuable for understanding the impact and sensitivity of different analysis strategies on the ability to detect differential gene expression, in the spirit of previous comparison studies performed using microarray and RNA-Seq data. We wanted to optimize our strategy in order to detect differential gene expression using the number of read counts per gene as a proxy. Several decisions must be made with regard to the filtering, normalization, and statistical analysis of read count data; any of these decisions may change the number and identity of differentially expressed genes. We therefore systematically examined the impact of three different filtering strategies, eight normalization methods, and two statistical approaches.

First, we wanted to remove genes having read counts below a reliable threshold of detection [2]. We derived an empirical threshold from the read count data [20] and examined the impact of removing read counts below this threshold both before and after normalizing the data.

Second, we wanted to choose the best normalization method for our read count data. We applied eight different normalization strategies to our read count data as in [21]: total counts (TC); upper quartile (UQ) [6], median (Med) [21], trimmed mean of M-values (TMM) [22], normalization using the *DESeq* package (DESeq) [13], quantile normalization (Q) [23, 24] RPKM [7], and remove unwanted variation (RUVg) [8]. We evaluated their effectiveness at reducing sources of bias as well as their impact on differential gene expression.

Third, we wanted to evaluate the ability of different statistical models to accurately detect differential gene expression. We applied a generalized linear model (GLM) to read counts fitted with a negative binomial distribution and compared this model to an analysis of variance (ANOVA) of read counts fitted with a log-normal distribution. We employed three software packages--*edgeR*, *DESeq*, and *SAS*--[13, 14, 25] to estimate differential gene expression.

These evaluations revealed important considerations to be made regarding the analysis of RNA-Seq read count data. Failing to consider these issues has a profound effect on the number and identity of genes designated as differentially expressed. The best analysis scheme for our data was to first normalize using the DESeq method and apply a generalized linear model assuming a negative binomial distribution using either *edgeR* or *DESeq* software. We found that the removal of low-expressed genes after the normalization and data distribution fitting procedures was the most flexible filtering strategy. Two normalization methods, DESeq and TMM, properly aligned the distribution of our data across samples and accounted for the dynamic range of our data [21]; however, TMM was sensitive to filtering strategy. In addition, we found that at least 3 biological replicates per genotype/environment/sex condition were required to have sufficient statistical power to detect gene expression differences, particularly among the three-way interaction of these factors.

## Results

### Application of low gene expression threshold

**Fig. 1:**
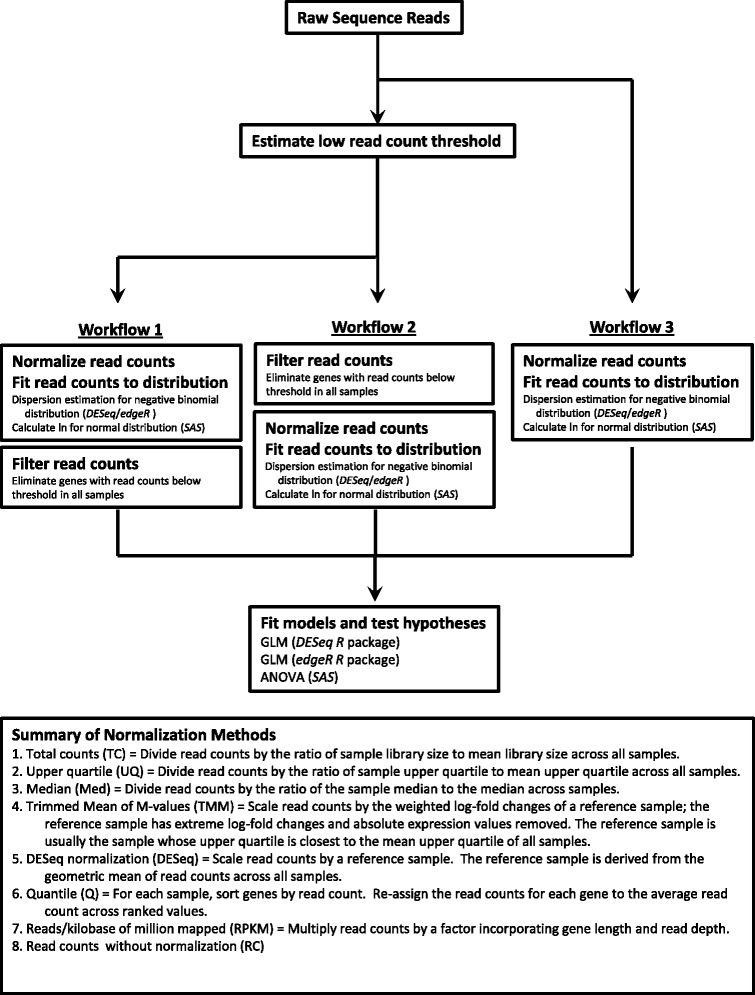
Flow chart showing analysis approach

**Fig. 2:**
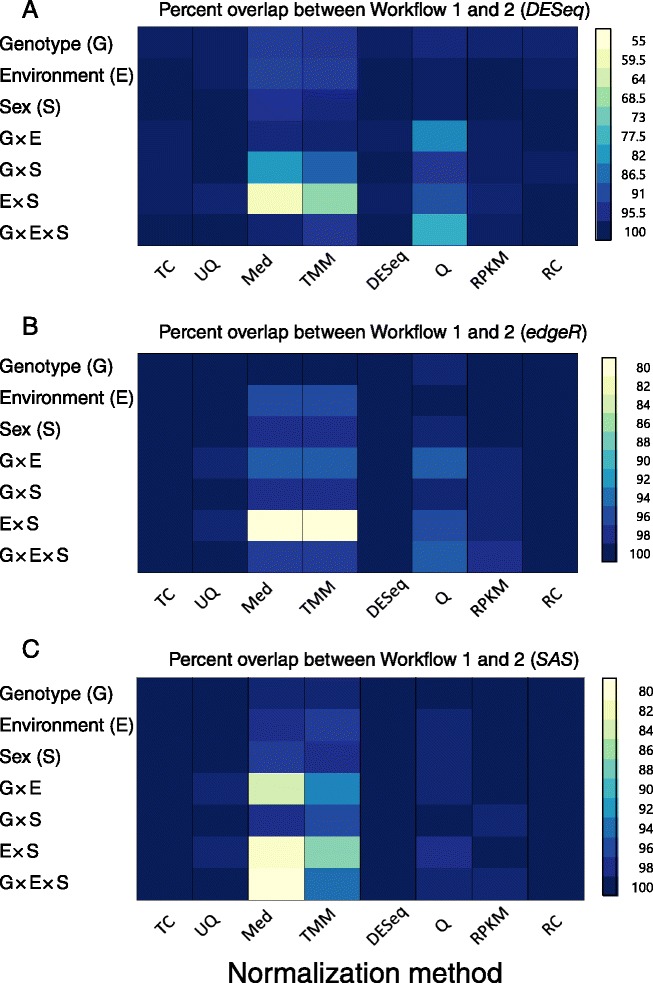
Effect of removing low-expressed genes before or after read count normalization on differential gene expression. The cell plot shows the percentage of agreement between Workflow 1 and Workflow 2. **a** Generalized linear model using *DESeq*. **b** Generalized linear model using *edgeR*. **c** ANOVA using *SAS*. Abbreviations for normalization methods are the same as defined in Fig. 1

It is well known that RNA-Seq read counts, which are presumed to be the signal of gene expression, contain a certain degree of uncertainty. This uncertainty is due to differences in library preparation among samples, sequencing error, sequence composition, and mapping/annotation errors. Very low read counts cannot be reliably distinguished from background noise [2]. Genes with very low expression might not be adequately represented across all fly samples; if this is the case, they are more likely to be incorrectly identified as differentially expressed. We therefore wanted to define an appropriate read count threshold from our data. We determined the low read count threshold for each normalization method separately (Methods and Additional file 1: Figure S1). We compared the distribution of intergenic read counts to those within the gene coding region [20]. We set the 95^th^ percentile of the intergenic read counts as the threshold of detection [20]. Low threshold values were comparable for all of the normalization methods, and were 3.40 log_2_ (normalized counts + 1) for TC, 3.59 for UQ, 4.52 for Med, 3.68 for TMM, 3.42 for DESeq, 3.77 for Q, 1.33 for RPKM, and 3.32 for the un-normalized read count data. Genes having read counts below these threshold values in all samples were removed from further analysis. Note that genes having read counts above the threshold level in one fly would be retained in the analysis using this criterion, even if all of the counts for the remaining 725 flies were below the threshold level. Normalization strategies often require the use of the entire data set to calculate the parameters for normalization. Whether the removal of genes from the analysis takes place before or after normalization could therefore potentially impact the number and identity of differentially expressed genes. We examined these effects using three workflows (Fig. 1). We normalized read count data and estimated distribution parameters first, then removed genes with read counts below the threshold value; we called this filtering strategy Workflow 1. We removed genes having read counts below the threshold value from the analysis first, then normalized the read count data and estimated distribution parameters; we called this filtering strategy Workflow 2. In addition, we analyzed the data without removing any low-expressed genes from the analysis; we called this filtering strategy Workflow 3. Figure 2 shows the impact of filtering out low-expressed genes before or after normalization and distribution parameter estimation for each statistical analysis approach. As Fig. 2 shows, the generalized linear model analysis using *DESeq* was the most sensitive to filtering strategy, while the ANOVA model was the least sensitive. Fewer genes overlapped between Workflow 1 and Workflow 2 for the generalized linear model than for the ANOVA model. Most of the normalization methods were robust to filtering strategy, particularly when the main effects of genotype, environment, and sex were considered. However, the Med, TMM, and Q normalization methods were sensitive to whether the removal of low-expressed genes occurred before or after the normalization/distribution parameter estimation step. First-order interaction terms normalized using these three methods were the most sensitive to filtering approach. In the case of Med-normalized read counts, the agreement of differentially expressed genes between Workflows 1 and 2 was as low as 59 % (Fig. 2a). Thus, some model terms and normalization methods were sensitive to low-expression filtering strategy. In contrast, the TC, UQ, DESeq, and RPKM normalization methods were robust to filtering strategy.

### Comparison of normalization strategies

**Fig. 3:**
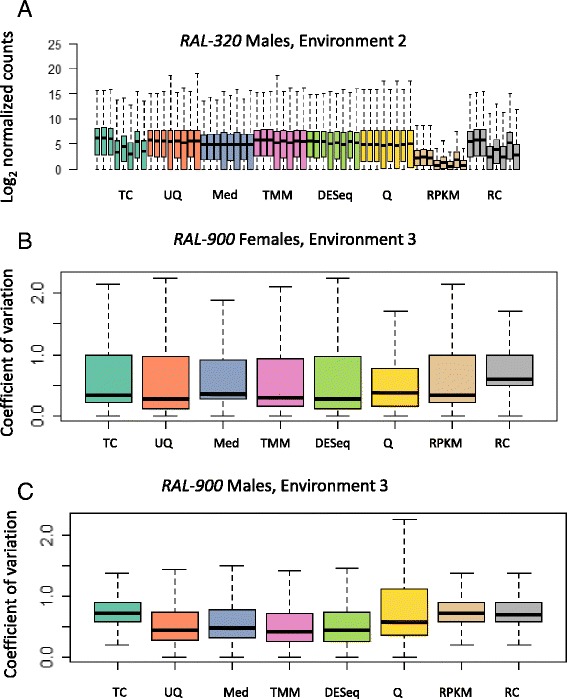
Examples of differences observed in normalization methods. **a** Boxplots of individual *RAL-320* males of Environment 2. **b** Boxplots of the coefficient of variation for *RAL-900* females of Environment 3. **c** Boxplots of the coefficient of variation for *RAL-900* males of Environment 3. A complete set of box plots can be found in Additional files 2 and 3. Abbreviations for normalization methods are the same as defined in Fig. 1. It has come to our attention that the line number designation for the Drosophila Genetic Reference Panel has been officially changed in Flybase. Specifically, the lines used to have a “*RAL*-” prefix; they now have “*DGRP*-” as the prefix (for example, “*RAL*-320” is now “*DGRP*-320”). We have used the “*RAL*-” prefix several times in our manuscript, in Fig. 3, and in Additional files 2 and 3. Future usage would be with the “*DGRP* –“prefix

We compared the performance of seven popular normalization methods for RNA-Seq read count data as in [21]: TC, UQ, Med, TMM, DESeq, Q, and RPKM. The TC method consists of dividing the read counts by a ratio of the library size for a given sample to that of the average library size across samples [9, 21]. Similar strategies are employed for the UQ and Med methods, where the ratios are the upper quartile and median read counts, respectively; these values are computed after removing genes having zero read counts for all samples [6, 21]. The TMM method scales read counts by the weighted log-fold-change values of a reference sample with genes that have extreme log-fold-changes (M values) and extreme absolute expression levels (A values) removed from the data. Usually, the sample whose upper quartile is closest to the mean upper quartile is chosen as the reference sample [22]. Likewise, DESeq scales the read counts by a reference sample based on the geometric mean of read counts across all samples [13]. Q normalization aligns the distribution of read counts across samples by ranking the read counts and then re-assigning the read counts for each gene to the average read count across ranked values [23, 24]. Finally, RPKM normalization multiplies read counts by a factor constructed from the gene length and read depth [24]. We compared these normalization strategies to the un-normalized read count data (RC). We used boxplots to display the data before and after normalization for each fly within genotype, environment, and sex (Fig. 3; Additional files 2 and 3). One of the purposes of normalization is to align the distribution of read counts across samples [6, 21, 22]; however, not all of the normalization methods successfully met this goal. Notably, the TC and RPKM methods did not improve the distribution of the data in many instances. For example, *RAL*-*320* males of Environment 2 do not show a change in distribution of read counts after normalization with either the TC or RPKM methods, consistent with previous observations [6, 21] (Fig. 3a). A second purpose of normalization is to reduce the within-condition variability due to background noise. The Q normalization method behaved differently from the other normalization methods in this respect. In some cases, Q normalization decreased within-condition variability for some samples (Fig. 3b), but added new variation to the data in other samples (Fig. 3c). In general, the UQ, Med, TMM, and DESeq methods improved some of the systematic biases in RNA sequence data. However, the number and identity of differentially expressed genes varied greatly across normalization procedures, as discussed below.

We noted that some of the highest read counts in our data set came from those aligned to non-protein coding genes. High read counts from rRNAs, pseudo-rRNAs and other RNA species were present in the data set; these species are present in the total RNA of each sample and may persist after library preparation if they have poly-A tails and/or poly-A selection is incomplete. We examined the impact of these species on differential gene expression. First, we removed all non-protein-coding genes from the data set of Workflow 1 DESeq- and TMM-normalized data, and compared the differential expression analysis results to that of the original data, which contained all of these species. The results were quite similar for DESeq-normalized data; 85 % or more of the differentially expressed genes overlapped. The results were somewhat less similar for TMM-normalized data; 73 % or more of the differentially expressed genes overlapped. Most of the differences in analysis were due to the removal of non-protein-coding genes that had significant differential expression in the original analysis (see Additional file 1: Table S1 for more detail). The number and identity of differentially expressed protein-coding genes was robust to the removal of non-protein-coding genes from the analysis. Thus, the DESeq and TMM normalization methods can compensate effectively for RNA-Seq data with a large dynamic range.

In addition, we explored the possibility of using the remove unwanted variation (RUVg) normalization method [8] for our data. This method uses spike-in (or negative) control genes to remove sources of technical variation such as differences in library preparation or variation among flow cells. The method considers these sources of technical variation as a covariate in the differential expression model. With this technique, a principal components analysis performed after normalization should reveal differences among experimental factors rather than the sources of technical variation [8]. A principal components analysis using the un-normalized data revealed that the first principal component, which explained 59.13 % of the biological variation among flies, was able to distinguish between the two sexes (Additional file 1: Figure S2A). The second principal component, which explained 25.53 % of the biological variation, was able to distinguish among the three environmental conditions to a very limited extent (Additional file 1: Figure S2A). We therefore used only the first principal component in the models for differential expression analysis. We found, however, that the sex and environmental conditions overlapped in the PCA plot using RUVg normalized read counts (Additional file 1: Figure S2D). This suggests that the technical variation is not the same between the spike-ins and the experimental sample. These differences may be a result of differences in our experimental approach and library preparation to that of [8] (see Discussion). As the technical variation between spike-ins and sample must be equivalent to use the RUVg method, we did not pursue this method further.

### Comparison of dispersion estimation methods

Unlike microarray data, RNA-Seq read count data do not have a continuous distribution. One common procedure is to use a Poisson distribution to model the data. However, a Poisson distribution assumes that the mean and the variance are equal, which is not an ideal fit to RNA-Seq data as they exhibit over-dispersion [5, 6, 13]. To apply the generalized linear model, we therefore modeled our read count data with a negative binomial distribution [13, 14, 26]. This distribution relies on an estimated dispersion parameter, which controls the relationship between the mean and the variance of the count data. The estimation of the dispersion parameter will affect the detection of differential genes reported by the generalized linear model. We used both *DESeq* and *edgeR* software to estimate the dispersion parameter for each gene. *DESeq* first calculates a dispersion value for each gene, and then fits a curve through the estimates [13]. Dispersion values are then assigned to each gene based on a choice between the greater of the per-gene estimate or the fitted value. *edgeR* estimates a common dispersion based on the Cox-Reid adjusted profile likelihood and then applies an empirical Bayes method to estimate the per-gene dispersion [12, 26]. The dispersion estimations for each normalization method using *DESeq* have a similar shape, with the exception of RPKM-normalized read counts (Additional file 1: Figure S3). RPKM-normalized counts also have a different pattern of dispersion when calculated by *edgeR* (Additional file 1: Figure S4). We also noted that dispersion estimations using Q normalization were different from the other normalization methods. We examined the effect of filtering strategy on dispersion estimates by plotting dispersion estimates of Workflow 1 versus Workflow 2. In most cases, the dispersion estimates for each workflow were well correlated, as expected. However, the correlations were different for RPKM- and Q-normalized data (Additional file 1: Figures S5 and S6). Workflow 1 dispersion estimations were higher than those of Workflow 2 for Q-normalized data regardless of the algorithm employed. However, Workflow 2 dispersion estimates were higher for RPKM-normalized data using *DESeq*, while Workflow 1 dispersion estimates were higher for RPKM-normalized data using *edgeR*. This suggests that dispersion estimates using Q and RPKM normalized data are sensitive to the filtering strategy. However, it should be noted that RPKM values cannot be directly input into either *DESeq* or *edgeR*; both programs expect read count data as input. We therefore input RPKM data as the normalized read counts rounded to be integers. This common practice impacts the dispersion estimates, which use the read counts for all samples to get each per-gene estimate. Because Q and RPKM normalization procedures use the entire data set, filtering and rounding effects are more apparent. The remaining normalization methods (TC, UQ, Med, TMM, and DESeq) had good agreement in dispersion parameters across workflows, and the resulting statistical analyses using *edgeR* and *DESeq* were also comparable (see below).

### Comparison of differential gene expression models

**Fig. 4:**
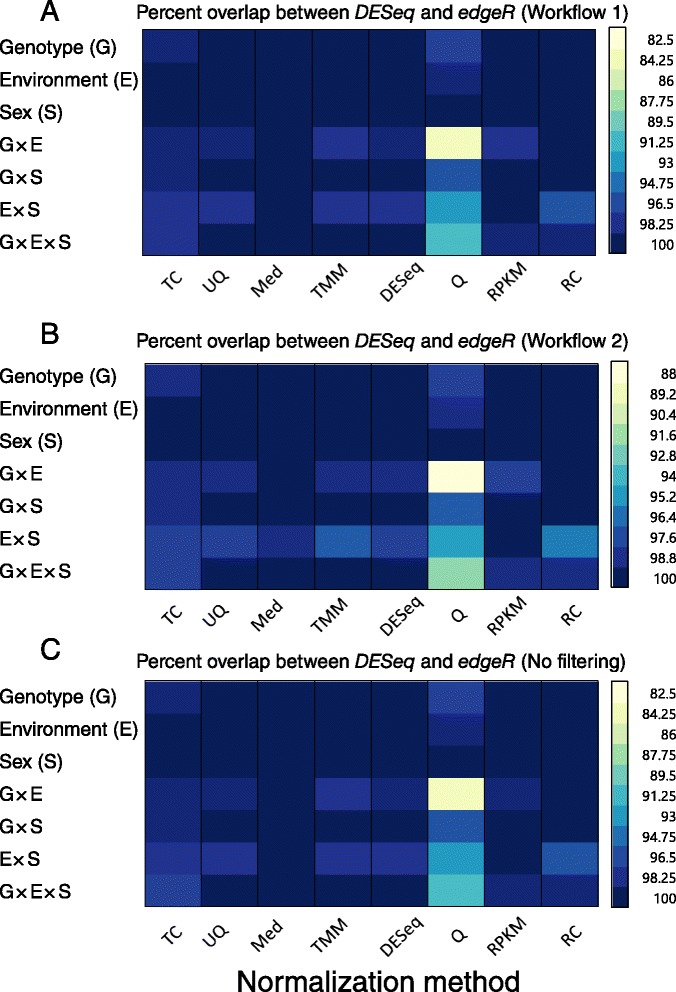
Comparison between the number and identity of differential genes estimated by *DESeq* and *edgeR*. The cell plot shows the percentage overlap between the two programs for each normalization method. **a** Workflow 1. **b** Workflow 2. **c** No filtering. Abbreviations for normalization methods are the same as defined in Fig. 1

We used two different models to determine differences in gene expression among flies: a generalized linear model using a negative binomial distribution and an analysis of variance (ANOVA) using a log-normal distribution. We used the *DESeq* and *edgeR* packages to estimate gene expression differences using a generalized linear model, and *SAS* to calculate the ANOVA parameters. Our models consider the main effects of Genotype, Environment, Sex, and their interactions. Table 1 shows the numbers of differentially expressed genes calculated for each factor in the generalized linear model using *DESeq* with the Workflow 1 strategy; these are compared across all normalization methods. The overlap of differentially expressed genes across all normalization methods was surprisingly low. Even when the un-normalized data were removed from the comparison, the agreement among normalization methods was as low as 10 % (i.e., compare the number of overlapping genes in Table 1 with the Med-normalized number for Enviroment × Sex). The lack of overlap stresses that the choice of a proper normalization method is crucial. We compared the *DESeq*-derived results from Table 1 to the numbers of differentially expressed genes calculated for the generalized linear model using the *edgeR* package. We compared the differences among software packages for Workflow 1 (Fig. 4a), Workflow 2 (Fig. 4b), or if the low-expression genes were left in the data set (Workflow 3; Fig. 4c). There were few, if any, differences in differentially expressed genes identified using these two programs across model factors, workflows, or normalization methods. Virtually every comparison was 96 % or greater in agreement. Only Q normalization showed any discrepancy in the genes identified by the two programs, and the differences were predominantly in the first- and second-order model terms. Thus, while *DESeq* and *edgeR* employ somewhat different algorithms, the number and identity of differentially expressed genes were in good agreement.

**Table 1:** Numbers of differentially expressed genes called by *DESeq* using the Workflow 1 generalized linear model. Numbers of genes are listed by analysis factor and normalization method

Factor\Method	TC	UQ	Med	TMM	DESeq^C^	Q	RPKM	RC	Overlap
Genotype (G)	10,765	10,110	11,133	11,425	**10,464**	11,226	8076	5471	4302^A^/6372^B^
Environment (E)	12,328	8821	10,146	9847	**9469**	9906	11,737	13,277	5779^A^/5819^B^
Sex (S)	14,303	14,840	13,042	14,850	**14,866**	14,515	13,553	14,018	9799^A^/10346^B^
G x E	3000	3265	1160	3158	**3463**	3147	896	1318	329^A^/483^B^
G x S	5942	6926	8441	8084	**7163**	7703	3414	2659	1771^A^/2700^B^
E x S	5539	2393	6533	3234	**2725**	4738	3032	82	62^A^/650^B^
G x E x S	2492	3410	1889	3501	**3611**	3767	1018	1202	652^A^/900^B^

*TC* total counts, *UQ* upper quartile, *Med* median, *TMM* trimmed mean of M-values, *DESeq* DESeq normalization method, *Q* quantile, RPKM reads per kilobase per million mapped, *RC* un-normalized count data

^A^ Numbers of overlapping genes among all methods

^B^ Numbers of overlapping genes among all methods except RC, the un-normalized count data

^C^Bold indicates preferred normalization method

**Fig. 5:**
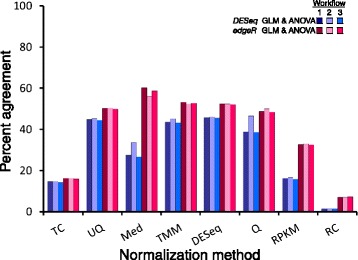
Comparison of the number and identity of differentially expressed genes obtained using the generalized linear model (GLM) with those obtained using the ANOVA model. The graph shows the percentage of differentially expressed genes for the Genotype × Environment × Sex term that agree between the GLM and ANOVA methods. Dark blue bars, overlap of *DESeq* GLM and ANOVA for Workflow 1; Light blue bars, overlap of *DESeq* GLM and ANOVA for Workflow 2; Medium blue bars, overlap of *DESeq* GLM and ANOVA for Workflow 3; Dark red bars, overlap of *edgeR* GLM and ANOVA for Workflow 1; Light pink bars, overlap of *edgeR* GLM and ANOVA for Workflow 2; Medium pink bars, overlap of *edgeR* GLM and ANOVA for Workflow 3. Abbreviations for normalization methods are the same as defined in Fig. 1

Striking differences were observed in the comparison of the generalized linear model with the ANOVA model. We compared the second-order term (Genotype × Environment × Sex) among these methods as the significance test has the same structure. Figure 5 shows the differences in differentially expressed genes identified by these methods for Workflows 1–3 and for each of the normalization methods. As Fig. 5 shows, the agreement across model types is quite low; as expected, the un-normalized read count data was the least consistent across models, averaging only 4.3 % agreement in differentially expressed genes. For every normalization method, there was better agreement between the *edgeR* GLM and the ANOVA than the *DESeq* GLM and the ANOVA. The greatest agreement was 60.4 % between the *edgeR* GLM and the ANOVA for Med-normalized data. Thus, the differences observed in the number and identity of differentially expressed genes hinge on the assumption of the underlying data distribution, and the assumption of a normal distribution, while robust in many types of data analysis, does not capture the dynamic range of RNA-Seq read count data.

The application of the generalized linear model necessitated decisions about the order in which the first-order interaction terms (Genotype × Environment, Genotype × Sex, and Environment × Sex) were evaluated in the model. However, the number and identity of differentially expressed genes can change depending on the order in which these terms are fitted to the model. We evaluated three different approaches to model these terms. In the first approach, we tested each first-order interaction term by sequentially adding it to the main-effect model (Methods). We added Genotype × Environment first, Genotype × Sex second, and Environment × Sex third. In the second approach, we fitted each first-order interaction term separately, using a model of main effect terms as the reduced model (Methods). In the third approach, we used a model with the main effects and all first order interaction terms, with the reduced model having each first order interaction term removed in turn (Methods). We compared the number and identity of differentially expressed genes for each of these approaches for both Workflows, the *DESeq* and *edgeR* analysis programs, and all normalization methods. The results of these comparisons are of Additional file 1: Table S2. It can be readily seen that the second approach yields the same answers for the Genotype × Environment interaction term as the first model; the third approach yields the same answers for the Sex × Environment interaction term as the first model. The number of differentially expressed genes for the Genotype × Environment interaction term was substantially reduced when all of the first-order interaction terms were fitted to the model (approach 3); thus, it explained less variation in the data. Also, after quality control, some of our samples were removed from the data set, giving us an unbalanced data set; a balanced data set would give us the same overlap using the first and third approach . Each of these approaches has merit, depending on the overall goal of the experiment; for example, if one is particularly interested in Genotype × Environment interactions, it would make sense to use the first approach in order to identify the largest potential subset of genes having significant Genotype × Environment effects.

### Statistical power calculations

**Fig. 6:**
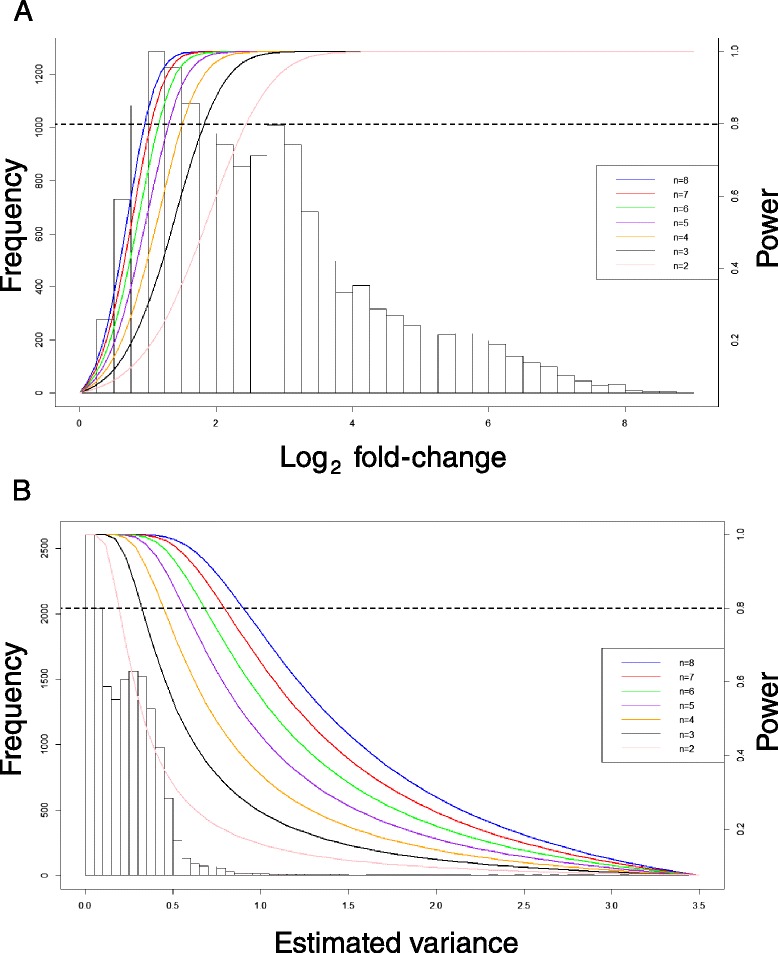
Statistical power analysis. **a** Detectable fold-change versus statistical power for *n* = 2, 3, 4, 5, 6, 7, and 8 flies per genotype/environment/sex. **b** Estimated variance

We based our differential expression analysis on eight individual flies per genotype/environment/sex. This is a higher number of samples per condition than would normally be anticipated in an RNA-Seq experiment. This approach ensured that we could detect subtle differences in gene expression. However, we wished to determine whether similar results could be obtained using a smaller sample of flies. Figure 6a shows how the statistical power to detect second-order differential gene expression varies with sample sizes of 2, 3, 4, 5, 6, 7, and 8 flies per genotype/environment/sex. For a statistical power of 80 %, it can be seen that a sample size of 2 flies per condition would reliably detect gene expression differences only if those differences were 2.5-fold or greater. Three flies would detect 1.8-fold or greater changes. Estimated variance is reduced for 2 flies per condition versus 8 flies per condition when fold-change was held constant (Fig. 6b). The required number of flies for detection of differential gene expression is somewhat lessened for first-order terms (Additional file 1: Figure S7). Two flies per condition would detect 1.75-fold or greater changes; three would detect 1.4-fold changes. These results indicate that using three flies per condition would be adequate for detecting large (1.8-fold or greater) changes among experimental factors. Our data were far more sensitive, however; we could detect 0.9-fold differences in expression for the three-way interaction term.

**Fig. 7:**
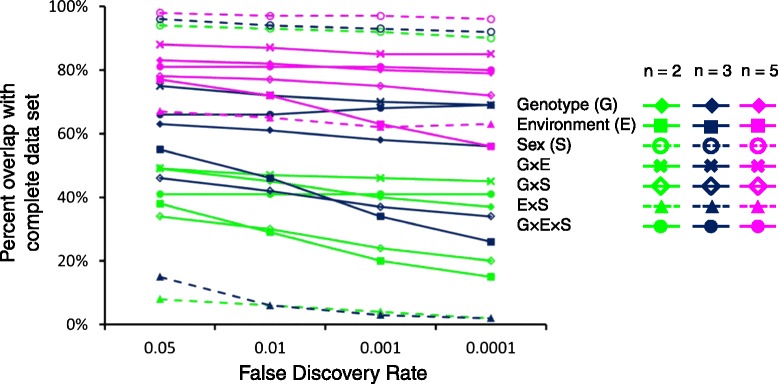
Empirical analysis approach using a reduced data set. The percentage of genes overlapping with the full data set is plotted against different false discovery rate thresholds for *n* = 2, 3, and 5 flies per genotype/environment/sex

Our power calculation was based upon the ln&ANOVA approach so that we could calculate the statistical power for the first and second-order terms we were interested in (See Methods). Current methods to calculate the statistical power for RNA-Seq read counts assuming a negative binomial distribution are limited to a single factor [27, 28]. However, it is possible to examine the impact fewer flies would have on the on the number and identity of differentially expressed genes using an empirical approach, as has been applied previously to microarray data [16, 18]. We randomly sampled our data for 2, 3, and 5 flies per genotype/environment/sex condition and ran the generalized linear model for Workflow 1 using DESeq-normalized read counts. We compared the percentages of differentially expressed genes that overlap with the full data set for different false discovery rate thresholds [16] (Fig. 7). This analysis shows a greater level of agreement with the complete data set when greater numbers of flies are analyzed per condition, as expected. The percentage agreement tended to drop slightly as the false discovery rate was reduced. Note also that the percentage of overlap depended upon the factor being studied. For example, using even 2 flies per condition would be sufficient to produce the same estimate of differentially expressed genes between males and females as the full data set, but at least 5 flies per condition would be required to estimate differential expression for the first-order Environment × Sex interaction term. Thus, while we were not able to calculate the statistical power directly for a negative binomial distribution, the empirical approach indicates that at least 3 flies per condition would be required to sufficiently estimate differential expression for most of the factors we considered.

## Discussion

Here we applied three different filtering strategies, eight normalization methods, and two statistical models to RNA-Seq read count data. We derived an empirical low gene expression threshold using both genic and intergenic read count data, and we estimated the numbers of biological replicates necessary to detect differential gene expression. In most instances, the number and identity of differentially expressed genes were robust to the method employed. However, certain manipulations of the data can profoundly alter the results. Our comparisons reveal the key decisions to be made in the analysis of RNA-Seq data and the consequences of ignoring those decisions. We address filtering strategy, normalization methods, model comparison, and statistical power considerations below.

### Filtering strategy

Whether the statistical analysis results were robust to filtering strategy (Workflows 1 and 2) depended upon the factor of interest in the statistical model. The main effects of Genotype, Environment, and Sex were not sensitive whether low-expressed genes were removed before or after normalization. However, the first- and second-order interaction terms were sensitive to filtering; in particular, the Med, TMM, and Q normalization methods showed less agreement in the number and identity of differentially expressed genes between Workflows 1 and 2. The sensitivity of these normalization methods in particular results from the fact that all of the read count data in the data set is used to calculate normalization parameters. Clearly, the median of the data set will change if the low-expressed genes are removed before normalization, making the Med read count data different in Workflow 1 versus Workflow 2. Likewise, the TMM method uses subsets of genes in the middle 40 % and 90 % of the read count data to calculate the normalization parameter; the removal of low-expressed genes changes how the middle 40 % and 90 % are defined. Finally, the Q normalization assigns new read count values based on the rank order of the read counts. Removal of the smallest read counts will change this data set as well. In contrast, use of the remaining normalization methods lead to very similar results whether using Workflow 1 or Workflow 2. We prefer the Workflow 1 strategy of removing low-expressed genes after the normalization step, because it gives us the option to apply different low-expression thresholds without the necessity of repeating the statistical analysis; only the false discovery rates need be re-calculated. The removal of low-expressed genes may constitute a *de facto* statistical test, resulting in an increase in the true false-positive rate [29]. However, we found that the majority of differentially expressed genes in our data set were the same whether the data were filtered or not, which is likely due to 1) the increased statistical power from the large sample size in our data set, and 2) the fact that the filter did not remove many genes. For example, only 5.7 % of the genes were removed from the analysis in Workflow 1 DESeq-normalized data. (See Additional file 1: Table S3 for a comparison of Workflow 1 with Workflow 3 for DESeq-normalized data). It is not necessary to devise a filtering strategy for all RNA-Seq experiments. For example, some RNA-Seq experiments profile small numbers of genes, or focus on rare transcripts. In these cases, a filtering strategy would not be warranted. However, there is a risk of an increased number of false positives if small numbers of read counts for a gene are detected in only one sample.

### Normalization methods

We noted that the TC and RPKM normalization methods were not effective at aligning the distribution of read counts across samples, an issue reported previously [21]. One reason for this lack of improvement can be observed in the distribution of our read count data. Fifty percent of the read counts for males align to only 45 genes, while fifty percent of the read counts in females align to only 186 genes. Similar distributions of read counts have been observed in other RNA-Seq data sets, including data from other species [5, 21]. Using simulated data, [21] noted that TC, UQ, Med, Q, and RPKM normalization methods were not able to control false positives in data having a small number of genes with very high read counts. In contrast, the DESeq and TMM normalization methods are designed to account for these extreme differences in read count number [13, 22]. TMM in particular has adjustable parameters that could be used to account for data with a large dynamic range [22]. Note that here we used only the default settings of these parameters, but we could have adjusted the parameters to improve the alignment across samples. Many of the genes having the highest read counts in our data set are non-protein coding genes such as rRNAs, pseudo-rRNAs, etc. The presence of these additional types of RNA is due to non-specific binding of poly-A RNA species to the oligo-dT beads used during library construction; we did not completely eliminate these species in our RNA-Seq library preparations. This issue could potentially be alleviated in the future with double polyA isolation, which would require the use of more total RNA, or by using protocols that would eliminate ribosomal RNA species. We found good agreement in the number and identity of differentially expressed genes in the DESeq- and TMM-normalized data sets whether these non-protein coding genes were retained in the data set or not. Thus, it is important to use a normalization method that will account for extreme values in the data if the specific RNA extraction and library preparation processes used cannot eliminate other, potentially abundant species that may be present in the library due to non-specific binding. Alternatively, these species may be of interest biologically, and it would be advantageous to have methods that can account for them in the event they are retained in the data set.

RUVg normalization has the advantage of producing the same normalized read counts no matter what filtering strategy is used because the normalization parameters are constructed using only spike-in RNA. However, the technical variation did not affect both spike-ins and experimental sample equivalently in our data, which is a key assumption of the method [8]. We note that there are differences between our experiment and that of [8] that might have contributed to the differences in technical variation between the spike-ins and the experimental sample in our data. They systematically varied both library preparation and flow cell designation in their experiment, while our experiment did not. Further, they added spike-ins during the RNA isolation step, while we added the spike-ins after the polyA isolation step. We were thus unable to fully evaluate this normalization method.

In summary, we found that the TC and RPKM methods were not effective at aligning the read count distributions across samples [21]. Use of the UQ method may produce false positives [21]. The Med, TMM, and Q methods were sensitive to filtering strategy, although TMM is able to account for the large dynamic range in RNA-Seq read count data. For our data, the DESeq normalization method had the best properties: it aligned read count distributions across samples, reduced background noise, and accounted for the large dynamic range present in our data.

### Model comparison

We observed many differences in the number and identity of differentially expressed genes across the seven normalization methods. This is in contrast to [21], who documented more consistent results across normalization methods. We propose that there are two reasons for this difference. First, the data they used for their comparisons contained at most three replicates per condition. Our data had 8 flies per genotype/environment/sex condition, which greatly magnified the sensitivity of our data to the normalization method chosen. Second, the data they used examined differences in a single condition; our statistical model tested differences in gene expression across three main factors and their interactions. The additional complexity in our model revealed interacting factors that were more sensitive to normalization strategy. These observations suggest that comprehensive data sets used to address multi-factor conditions will be more sensitive to the choice of normalization. We observed very little difference between differentially expressed genes identified by the *DESeq* and *edgeR* programs using the same generalized linear model, regardless of normalization method and filtering strategy. Only the Q normalization method, which is an appropriate method commonly used for normalizing microarray data [23, 24], exhibited any substantive differences. Perhaps not surprisingly however, the differences between the generalized linear model and the ANOVA model were much higher. The underlying assumption for the generalized linear model is a negative binomial distribution [13], while the assumption for the ANOVA is a normal distribution. RNA-Seq read count data have a very wide dynamic range that is typically skewed towards low read count end of the distribution; such data do not fit a normal distribution very well [13]. In addition, our read count data were not homoscedastic for the log-normal distribution, which violates one of the basic assumptions for the use of an analysis of variance [30]. Thus, modeling RNA-Seq read count data with a negative binomial distribution is the most appropriate approximation.

### Dispersion estimation

Dispersion estimates were different for RPKM and Q normalized data, whether using either *DESeq* or *edgeR* software. But these normalization methods cannot be properly implemented using either *DESeq* or *edgeR*. Because both of these programs expect read count input, an integer value, a common practice is to perform the normalization step using another program and then round the normalized read count data to the nearest integer value. The dispersion estimates per gene are different whether using Workflow 1 or Workflow 2, so the rounding procedure in effect increases sensitivity. The authors of the *DESeq* and *edgeR* programs [S. Anders, personal communication, [14]] caution against using RPKM or Q-normalized data as input, yet the practice persists. The downstream effects of this practice can be seen by comparing the TC and RPKM normalization methods for unfiltered data. The only difference between these two normalization methods is the gene length, which is constant for a given gene across samples. Further, our generalized linear models compare the effects of Genotype, Environment, and Sex within a gene rather than among genes. Thus, we would expect the TC and RPKM normalization methods to give identical lists of differentially expressed genes when the data are unfiltered. However, this is not what we observed. The newer *DESeq2* program can be used to implement RPKM [31]. Note, however, that we were unable to use *DESeq2* with our large data set due to limitations in the program. Although *edgeR* and *DESeq* use different algorithms to estimate dispersion, the number and identity of differentially expressed genes were the same for DESeq- or TMM-normalized data (Fig. 4), indicating that either software package could be used for these estimates.

### Statistical power considerations

We calculated the sample sizes necessary to control the risk of making both Type I and Type II errors *post-hoc*. Here we used the ln&ANOVA model assuming a balanced design as the calculations for a three-factor model are relatively straightforward. Methods have been developed for the calculation of statistical power for RNA-seq data using a negative binomial distribution ([27, 28]; however, these calculations address comparisons made within a single factor. We were particularly interested in the ability to detect interactions among the factors we varied in the experiment, namely, Genotype, Environment, and Sex. Thus, we applied the ln&ANOVA calculation, but it should be realized that there were large differences between the differential gene expression analyses assuming a normal distribution and those assuming a negative binomial distribution. The extent to which the underlying data distribution assumption affects the statistical power of our data is unknown, and is a limitation of this analysis. We found that at 80 % power, two biological replicates (i.e., two flies) could only detect differences in expression of 2.5-fold or greater for the three-way interaction term, Genotype × Environment × Sex. Detection of differences was somewhat better for the Genotype × Environment term; two replicates could detect 1.75-fold or greater differences. The empirical study using 2, 3, and 5 flies per condition supports the notion of using three or more biological replicates in RNA-Seq experiments. This approach also shows a dependency on the factors being analyzed; for example, differences between male and female expression were more robust than differences across replicate environments. Often the choice of the number of biological replicates is not a function of statistical considerations, but economic ones. Furthermore, there is often a trade-off between experimental parameters such as the number of biological replicates, the number of genotypes or treatments, and the amount of sequence coverage. Previous studies have noted that adding biological replicates to the experiment is a more effective way to increase the statistical power to detect differential gene expression than increasing sequence depth [32, 33]. Increased biological replication can mitigate the number of false positives [32]; though both increased replication and sequencing depth improves the detection of genes with characteristically low expression [32, 33]. We demonstrated that three biological replicates produced a substantial improvement in fold-change detection over two; we recommend using at least three biological replicates if possible.

## Conclusions

Our investigation revealed that the most critical considerations for the analysis of RNA-Seq read count data were the numbers of biological samples, normalization method, and underlying data distribution assumptions. The statistical power analysis suggests that at least three replicates are required to detect differential expression among a three-way interaction of experimental factors. The sensitivity of our data set to differences in normalization method indicates that this is a crucial choice and that the normalization method chosen should account for the large dynamic range observed in RNA-Seq read count data. We recommend the DESeq or TMM normalization methods, noting that TMM is more sensitive to filtering strategy. A generalized linear model using a negative binomial distribution can be readily adapted to multi-factor comparisons using *DESeq* or *edgeR* software as we demonstrated here. The size and complexity of these data enable it to serve as a benchmark for RNA-Seq analyses. Future challenges include the incorporation of alternative splicing and polymorphism data into this analysis.

## Methods

### Sample collection and library preparation

We obtained RNA-Seq read count data from individual *Drosophila* Genetic Reference Panel (DGRP) flies [34, 35]. The details of the RNA extraction, and library preparation are provided in Additional file 4: Supplemental Methods. Briefly, we collected 8 virgin male and 8 virgin female flies from 16 DGRP genotypes for our study in three separate biological replicates. The genotypes examined were: *RAL*-93, *RAL*-229, *RAL*-320, *RAL*-352, *RAL*-370, *RAL*-563, *RAL*-630, *RAL*-703, *RAL*-761, *RAL*-787, *RAL*-790, *RAL*-804, *RAL*-812, *RAL*-822, *RAL*-850, and *RAL*-900. Flies were frozen after 7 days post-eclosion in 96-well plates. We replicated the experiment three times to produce 768 RNA sequences. To control the environmental conditions, we seeded the fly cultures with 5 males and 5 females; reared the flies in a single incubator on standard *Drosophila* food (Bloomington, IN) at 25 °C, 60 % humidity, and a 12:12-h light:dark cycle; collected male and female virgin flies to control mating status; maintained virgins at 20 to a same-sex vial for four days prior to RNA extraction to control for social exposure [36] and froze all flies for RNA extraction at the same circadian time (1:00 pm). We isolated total RNA using the RNeasy 96 Plate Kit (Qiagen, Valencia, CA) according to the manufacturer instructions using either vacuum or spin technology as modified in Additional file 4: Supplemental Methods. We added 96 synthetic ERCC spike-in control RNAs to the total RNA prior to library preparation. Strand-specific libraries 300–350 bp in size were prepared by modifying an existing protocol [37] (Additional file 4).

Variation in read counts among individual flies could be due to biological differences, or it could be due to technical variation in library preparation and sequencing. We prepared duplicate RNA-Seq libraries for 118 flies chosen randomly. To determine whether the read count differences observed among flies were biological or technical we fit a generalized linear model to the DESeq-normalized read count data. The model considered each individual fly (F) as a factor and the duplicate RNA-Seq libraries as replicates within the factor.

$$ \log \left({\mu}_{\mathrm{ik}}\right) = {\beta}_0 + F $$

While 9495 genes were differentially expressed among the individual flies, none of the ERCC spike-in controls were differentially expressed, indicating the presence of large biological rather than technical effects (FDR <0.05). We further examined technical differences by plotting the absolute difference in raw (un-normalized) read counts between replicate libraries for each sample (Additional file 1: Figure S8). Differences between libraries are less than our low-expression threshold (the 95^th^ percentile of intergenic raw read counts), indicating that there is little technical difference between libraries.

### Quality assurance procedures

We used the raw sequence data to verify the sequence pool index, genotype, and sex labeling of each fly, to define a threshold for low (undetectable) gene expression, and to assess the technical variance between library preparations. We searched the raw sequence data for all 24 indices used in the experiment in order to confirm the expected index and to identify any contaminating indices. We retained all samples with 95 % or greater of the expected index in the analysis. The DGRP lines are fully sequenced [34, 35]; thus, we were able to use known single nucleotide polymorphism (SNP) sites to verify the genotype of each fly. Base calls at 2,192,560 informative SNP locations are known for all 16 DGRP lines. Base calls were extracted from the sequence data using SAMtools mpileup [38] for those SNP sites having more than two reads. We required the base calls to be present in greater than 95 % of the reads with less than 5 % technical errors from sequencing. 1000 SNP sites across the genome having a base call in the greatest number of samples were chosen to uniquely identify each DGRP line. We calculated the differences in SNPs between each fly sample and known SNPs in each DGRP line using two measurement variables *r*_ij_ and *R*_ij_.

$$ {r}_{ij}=\frac{D_{ij}}{M_{ij}} $$

,where *D*_*ij*_ is the number of mismatched SNP sites between sample *i* and DGRP line *j* and *M*_*ij*_ is the number of matched SNP sites between sample *i* and DGRP line *j*.${R}_{ij}=1-\frac{r_{ij}- \min \left({r}_{i.}\right)}{max\left({r}_{i.}\right)- \min \left({r}_{i.}\right)}$, where max(*r*_*i*._) is the maximum *r*_*ij*_ over sample *i* and all DGRPlines, and min(*r*_*i*._) is the minimum of *r*_*ij*_ over sample *i* and all DGRP lines. *R*_*ij*_ has a value with the range [0,1], where *R*_*ij*_ = 1 when *r*_*ij*_ = min(*r*_*i*._) and *R*_*ij*_ = 0 when *r*_*ij*_ = max(*r*_*i*._). The genotype of each fly was assigned to the DGRP line having an *R*_*ij*_ = 1. We used the 5 % level of the distribution of *r*_ij_ across genotypes, 0.10, as the threshold for the number of acceptable mismatched SNPs. We included all fly sequences that could be assigned to their expected DGRP line in subsequent analyses.

Some genes present on the Y chromosome have duplicates in other regions of the genome, and the Y chromosome is gene-poor; thus, mapping sequences to the Y chromosome is not a reliable indicator of sex. We used the well-known high levels of sex dimorphism in *Drosophila* gene expression [39–46] to verify the sex of each fly. We defined a male standard sample as the median value of normalized read counts for each gene across all male samples, and defined a female standard sample in the same manner. We calculated the Spearman correlation coefficients of normalized read counts between each sample fly and each sex standard. The comparison revealed that 95 % of the flies had a correlation of 0.795 or less with their opposite sex standard. We therefore required a correlation of 0.795 or greater for each fly with its same-sex standard as the threshold for sex verification. We eliminated the sequence of any fly that did not pass the genotype and sex quality checks, samples that failed RNA extraction or library preparation, and samples that did not have at least 2.5 million uniquely mapped reads (ModENCODE Consortium, personal communication); this left us with sequence data for 726 flies. This data set and additional information is available in the NCBI Gene Expression Omnibus (GEO) under the accession number GSE60314.

### Empirical low-expression threshold determination

We defined a gene expression threshold based on a comparison of the distribution of read counts in annotated gene regions (Flybase annotation 5.57) [47] to read counts observed in intergenic regions. We compiled read counts from all intergenic regions. We removed all intergenic regions smaller than the read length of 76 bp, as there will not be unique reads for these regions. Because any normalization method used will alter the distribution of read counts, we made separate distribution plots for each normalization method and for the un-normalized count data. We combined the genic and intergenic data and normalized it, then plotted the distributions separately. We chose the 95^th^ percentile of the intergenic distribution as the low threshold level of gene expression [20]. We removed those genes having normalized read counts below the low threshold level in all samples. When this filtering strategy was applied, it was applied to normalized read counts from the genic regions only. This analysis can be implemented using the pipeline in Additional file 5.

### RNA sequence normalization

We examined the impact that read count normalization methods have on the identification of differentially expressed genes. We considered the effect of seven popular normalization methods used in RNA-sequencing data analysis as well as the un-normalized number of reads that mapped uniquely to each gene. We applied total count normalization (TC) [9, 21], upper quartile normalization (UQ) [6], median normalization (Med) [21], full quantile normalization (Q) [23, 24], reads per kilobase per million mapped reads (RPKM) [7], trimmed mean of M-values (TMM) [22], and the normalization method supplied in the DESeq package (DESeq) [13] (see Additional file 5 for pipeline). Here we defined the un-normalized number of reads (counts) that mapped uniquely to each gene as the raw count data (RC). To use the TC, UQ, and Med normalization methods, the raw count data in each sample is divided by a ratio. For the TC method, the ratio is the total number of mapped reads for a given sample divided by the mean total number of mapped reads across all samples. Likewise, the UQ ratio is the upper quartile of the raw count data across all genes in each sample divided by the mean upper quartile across all samples. In addition, the Med ratio is the median read counts for all genes in a given sample divided by the median read across all samples. Both the upper quartile and the median ratios are calculated after removing genes with zero read counts across all samples from the data. The Q normalization equalizes the distribution of raw counts across samples by ranking the raw counts for each gene in each sample and applying a new mean count for each gene based upon rank. RPKM normalization is widely used for RNA-Seq data and consists of multiplying the raw counts for each gene in each sample by a factor incorporating both sequencing depth and gene length [7]. The trimmed mean of M-values (TMM) normalization [22] is accomplished in two steps. In the first step, the gene-wise log fold-changes (M-values) and absolute expression levels (A-values) are calculated, respectively, where

$$ {M}_{ik}^r={ \log}_2\left(\frac{x_{ik}/{N}_k}{x_{ir}/{N}_r}\right) $$

and${A}_{ik}^r=\frac{1}{2}\left({ \log}_2\left({x}_{ik}/{N}_k\right)+{ \log}_2\left({x}_{ir}/{N}_r\right)\right)$ for *x*_*i*._ ≠ 0. *x*_*ik*_ and *x*_*ir*_ are the read counts for gene *i* of sample *k* and the reference sample respectively, while *N*_*k*_ and *N*_*r*_ are the sample sizes of sample *k* and a reference sample respectively. We used the default reference sample, which is the sample having the upper quartile most similar to the mean upper quartile across all samples. The genes in the middle 40 % of the M-values and the middle 90 % of the A-values are recorded. The list of genes overlapping in these two groups of genes is designated as G^*^. Note that the user may choose M-value and A-value percentages other than 40 % and 90 %, respectively; these are the default percentages. In the second step, a normalization factor is calculated as the weighted mean of M-values for each sample, which is:

$$ \frac{{\displaystyle \sum_{i\in {G}^{*}}{w}_{ik}^r{M}_{ik}^r}}{{\displaystyle \sum_{i\in {G}^{*}}{w}_{ik}^r}} $$

*w* is the weight calculated as the inverse of approximate asymptotic variance as given by the following expression:

$$ {w}_{ik}^r=\frac{N_k-{x}_{ik}}{N_k{x}_{ik}}+\frac{N_r-{x}_{ir}}{N_r{x}_{ir}} $$

To obtain the TMM-normalized read counts when using the *DESeq* program, we also divided the normalized read counts by the mean of the normalized library size [21].

Like the TMM normalization, DESeq normalization requires a reference sample to calculate the scaling factor for normalization [13]. DESeq constructs the reference sample as the geometric mean of raw counts across all samples for each gene. The scaling factor for each sample is then calculated as the median of the ratio of raw counts of the sample and the reference sample across all genes.

In addition to the seven normalization methods applied above, we also considered a recently published normalization method called remove unwanted variation with negative control genes (RUVg) [8]. RUVg normalization assumes that a set of negative control genes is available and the expression of these negative control genes are affected by technical, but not biological, sources of variation in the same way as gene read counts. RUVg normalization constructs the factors that capture technical variation from negative control genes, which are treated as additional covariates in the models for differential expression analysis. We used External RNA Control Consortium (ERCC) spike-ins during library preparation [5]; 32 of these spike-ins were added across all samples and did not vary with biological sources of variation when compared as replicate libraries. We used these 32 ERCC spike-ins as negative control genes and applied the *RUVSeq* R package [8] to normalize our read count data.

### Count data distribution estimations

We modeled the count data as both a negative binomial distribution and as a normal distribution. To model the count data as a negative binomial (*NB*) distribution, [13, 26, 48], we assumed that the number of read counts for gene *i* in sample *k* can be modeled by

$$ {x}_{ik}\sim NB\left({\mu}_{ik},{\sigma}_{ik}^2\right) $$

where *μ*_*ik*_ is the mean, and *σ*_*ik*_^2^ is the variance. The mean is

$$ E\left({x}_{ik}\right)={\mu}_{ik} $$

and the relation between variance and mean is given as:

$$ {\sigma}_{ik}^2={\mu}_{ik}+{\mu}_{ik}^2{\varphi}_i $$

The dispersion parameter ϕ_*i*_ determines the extent to which the variance exceeds the mean. We used the *DESeq* and *edgeR* packages to estimate the dispersion parameter [13, 14] (Additional file 5).

Another strategy for RNA-Seq count data analysis is to model a normal distribution by ln-transforming normalized count data. This is done by simply taking the ln of the read count data then applying standard microarray analysis techniques [49, 50] using the *limma R* package (Additional file 5). We used both the negative binomial and the normal distribution to model the read count data.

### Model fitting and hypothesis testing

To understand how gene expression varies among individual flies, we tested each gene for differential expression among DGRP genotype, environment, sex, and their interactions. For count data modeled with a negative binomial distribution, we fitted the following generalized linear model (GLM) for each gene *i*:

$$ \log \left({\upmu}_{\mathrm{ik}}\right)\kern0.37em ={\beta}_0 + S + G + E + G \times E + S \times G + S \times E + S \times G \times E $$

where *S* is sex, *G* represents the DGRP genotype, and *E* is the environmental condition. To test the significance of all factors in the model, we fitted the following series of models:

$$ Model\;1:\; \log \left({\mu}_{\mathrm{ik}}\right)\kern0.37em ={\beta_0}^1 + S + G + E $$

$$ Model\;2\left(\mathrm{a}\right):\; \log \left({\mu}_{\mathrm{ik}}\right)\kern0.37em ={\beta_0}^1 + S + G + E + G \times E $$

$$ Model\;2\left(\mathrm{b}\right):\; \log \left({\mu}_{\mathrm{ik}}\right)\kern0.37em =\kern0.37em {\beta_0}^1 + S + G + E + G \times E + G \times S $$

$$ Model\;2: \log \left({\mu}_{\mathrm{ik}}\right)\kern0.37em ={\beta_0}^1 + S + G + E + G \times E + G \times S + E \times S $$

To test each term of the main effects, we used *Model 1* as the full model, and calculated the likelihood ratio between *Model 1* and *Model 1* with each of the main effects removed in turn, which we term the reduced *Model 1*. The likelihood ratio statistic comparing these two models is simply the difference between the deviances of the full model and the reduced model

$$ -2\left[L\left({\widehat{\upmu}}_{ikR},{\widehat{\upvarphi}}_i;x\right)-L\left({\widehat{\upmu}}_{ikF},{\widehat{\upvarphi}}_i;x\right)\right] $$

To test the two-way interaction terms *G* × *E*, *G* × *S*, and *E* × *S*, we used the same approach; we added each term to be tested in turn, defining it as the full model, and compared it to the previous reduced version of the model. For example, *Model 2(b)* and *2(a)* were used to find genes with a significant *G* × *S* interaction; *Model 2(b)* was the full model, while *Model 2(a)* was the reduced model. To test the significance of the three-way interaction term *S* × *G* × *E*, we used the same approach, where *Model 2* was the reduced model. Inspection of the *Model 2*, *2(a)*, and *2(b)* equations above suggests that differential expression detected for each first-order interaction term is dependent upon its ordering in the equation. We therefore compared this analysis with two other ways of detecting differential gene expression for first-order interaction terms. In the second approach, we used the *Model 1* as the reduced model and then added each first-order interaction term in turn to *Model 1* to test the significance of each first-order interaction term. In the third approach, we assessed the contribution of each first-order interaction term by using *Model 2* as the full model and *Model 2* without each of the first-order interaction terms in turn as the reduced model.

In addition to using the GLM with negative binomial distribution to model the count data, we also evaluated the ln-transformation of the normalized count data combined with analysis of variance (ANOVA), which we called the ln&ANOVA method. We ln-transformed the normalized read counts and then fitted the ANOVA model below using *SAS* (version 9.3) [25]:

$$ \ln \left(\mathrm{normalized}\ \mathrm{count} + 1\right)={\beta}_0+S+G+E+G\times E+S\times G+S\times E+S\times G\times E + \varepsilon $$

where *S*, *G*, and *E* are as defined above, and *β*_0_ is the intercept, while ε is error.

### Correction for multiple tests

The Benjamini-Hochberg procedure [51] was used to control the false discovery rate (FDR) based on the *P*-values obtained from the analysis. Genes having *P*-values with an FDR threshold of < 0.05 were designated as differentially expressed (Additional file 5).

### Statistical power calculations

For a fixed-effect multi-factor ANOVA model, the test statistic has an *F* distribution under the null hypothesis [52]. The test statistic has a non-central *F* distribution with non-centrality parameter φ when the null hypothesis is false [52]. Thus, the power of an *F* test is the probability that the observed test statistic is greater than a critical value of the test, where the probability is calculated using the significance level and non-centrality parameter λ (or φ). Given an ANOVA model with three fixed factors [52, 53], the non-centrality parameter for testing the three-way interaction term with balanced design is given as $\lambda =\frac{{\displaystyle \sum_{i=1}^a{\displaystyle \sum_{j=1}^b{\displaystyle \sum_{k=1}^c{\left(\alpha \beta \gamma \right)}_{ijk}^2}}}}{\sigma^2/n}$ or ${\varphi}^2=\frac{n{\displaystyle \sum_{i=1}^a\sum_{j=1}^b\sum_{k=1}^c{\left(\alpha \beta \gamma \right)}_{ijk}^2}}{\sigma^2\left[\left(a-1\right)\left(b-1\right)\left(c-1\right)+1\right]}$, where a, b, c are the number of conditions for the three main effects (i.e., a = 16, b = 3 and c = 2), and (*αβγ*)_*ijk*_ is the difference between the condition mean and the value that would be expected if main effects and two-way interaction terms are sufficient to account for all factor effects. By introducing a new parameter $d=\frac{ \max \left({\mu}_{ijk}\right)- \min \left({\mu}_{ijk}\right)}{\sigma }=\frac{D}{\sigma }$ [54], it can be shown that the minimum value of λ is $\frac{n{d}^2}{2}$, that is $\frac{n{D}^2}{2{\sigma}^2}$, where *μ*_*ijk*_ refers to the mean of the three-way interaction condition for the first factor at the *i*^th^ level, the second factor at the *j*^th^ level and the third factor at the *k*^th^ level. For our data, *μ*_*ijk*_ is the mean of ln-transformed normalized counts under the condition of *i*^th^ genotype, *j*^th^ environmental condition and *k*^th^ sex; D is called the fold-change. Hence we can calculate a conservative power estimate using the ln-transformed normalized counts, the desired significance level, sample size (1–8 flies), and variance σ^2^ (as estimated by the mean sum of squares).

### Implementation of analysis

Additional file 5 provides the R code used to implement these analyses.

### Ethics statement

The research performed in this study on the fruit fly, *Drosophila melanogaster*, did not require approval from an ethics committee.

## Availability of supporting data

All RNA-seq data from this study are available from the NCBI Gene Expression Omnibus (GEO) under the accession number GSE60314.

## Additional files

Additional file 1:
**Contains Figures S1-S8 and Tables S1-S3.** (PDF 1892 kb) Additional file 2:
**Comparison of normalization methods for each fly.** Boxplots show the differences in the read count distribution for each fly. Flies are grouped by genotype, sex, and environment. (PDF 437 kb) Additional file 3:
**Comparison of normalization methods across conditions.** Boxplots show the differences in the coefficient of variation across flies in each genotype/sex/environment condition. (PDF 245 kb) Additional file 4:
**Supplemental Methods.** (PDF 133 kb) Additional file 5:
**R code pipeline for analysis.** (PDF 245 kb)

## Abbreviations

Med: median normalization
Q: quantile normalization
RC: un-normalized read counts
RPKM: reads per kilobase of million mapped reads
RUVg: remove unwanted variation
TC: total counts
TMM: trimmed mean of M-values
UQ: upper quartile
