# Supplementary material for: Comparison of normalization and differential expression analyses using RNA-Seq data from 726 individual Drosophila melanogaster
Source: BMC Genomics. 2016 Jan 5;17:28. doi: 10.1186/s12864-015-2353-z (PMC4702322; doi:10.1186/s12864-015-2353-z)
Supplement: Additional file 1: — Contains Figures S1-S8 and Tables S1-S3. (PDF 1892 kb) [file 12864_2015_2353_MOESM1_ESM.pdf]

Additional File 1. This file contains Figures S1-S8 and Tables S1-S3.

Figure Legends

Figure S1. Low gene expression threshold determination. For each normalization method, the distributions of genic and intergenic normalized read counts are shown. The genic distribution is pink, the intergenic distribution is blue, and the overlap of both distributions can be seen as purple bars. The red vertical line marks the location of the 95th percentile of the intergenic distribution, which is the low expression threshold. (a) Total counts (TC). (b) Upper quartile (UQ). (c) Median (Med). (d) Trimmed mean of M-values (TMM). (e) DESeq normalization. (f) Quantile (Q). (g) RPKM. (h) Un-normalized read count data.

Figure S2. Principal components plot for RUVg normalization. Plots show the first, second and third principal components plotted against each other. (a) PC1 vs. PC2 for the un-normalized data. (b) PC1 vs. PC3 for the un-normalized data. (c) PC2 vs. PC3 for the un-normalized data. (d) PC1 vs. PC2 for the RUVg-normalized data. (e) PC1 vs. PC3 for the RUVg-normalized data. (f) PC2 vs. PC3 for the RUVg-normalized data. Light red, females of environment 1; dark red, males of environment 1; light green, females of environment 2; dark green, males of environment 2; light blue, females of environment 3; dark blue, males of environment 3.

Figure S3. Coefficient of dispersion estimation using *DESeq*. Plots show the estimated dispersion coefficient plotted against the mean normalized read counts for each gene. Green dots indicate genes that pass the low expression threshold; black dots indicate genes that would be removed from the data set as they do not pass the low expression threshold. A red line shows the curve fit to the data. (a) TC. (b) UQ. (c) Med. (d) TMM. (e) DESeq. (f) Q. (g) RPKM (h) un-normalized read count data.

Figure S4. Coefficient of dispersion estimation using *edgeR*. Plots show the biological coefficient of variation (the dispersion parameter) plotted against log of the counts per million for each gene. Green dots indicate genes that pass the low expression threshold; black dots indicate genes that would be removed from the data set as they do not pass the low expression threshold. A red line shows the common dispersion based on the Cox-Reid adjusted profile likelihood. (a) TC. (b) UQ. (c) Med. (d) TMM. (e) DESeq. (f) Q. (g) RPKM. (h) un-normalized read count data. (j-p) Plots are shown in the same order as A-H with axes not to the same scale.

Figure S5. *DESeq* dispersion estimation comparison between Workflows 1 and 2. Plots show the similarities and differences in the dispersion estimation between the two workflows. (a) TC. (b) UQ. (c) Med. (d) TMM. (e) DESeq. (f) Q. (g) RPKM. (h) Un-normalized read count data.

Figure S6. *edgeR* dispersion estimation comparison between Workflows 1 and 2. Plots show the similarities and differences in the dispersion estimation between the two workflows. (a) TC. (b) UQ. (c) Med. (d) TMM. (e) DESeq. (f) Q. (g) RPKM. (h) Un-normalized read count data.

Figure S7. Statistical power estimates for the Genotype×Environment interaction term.

Figure S8. Comparison of RNA-seq data from flies with two library preparations. The difference in raw (un-normalized) read counts between all genes is plotted as the $\log_2$ of the absolute difference plus one for 117 flies in the experiment that had two library preparations, and one fly that had three libraries. (a) read counts for each gene and ERCC spike-in RNAs combined; (b) read counts for each gene; (c) read counts for ERCC spike-in RNAs only.

Figure S1.

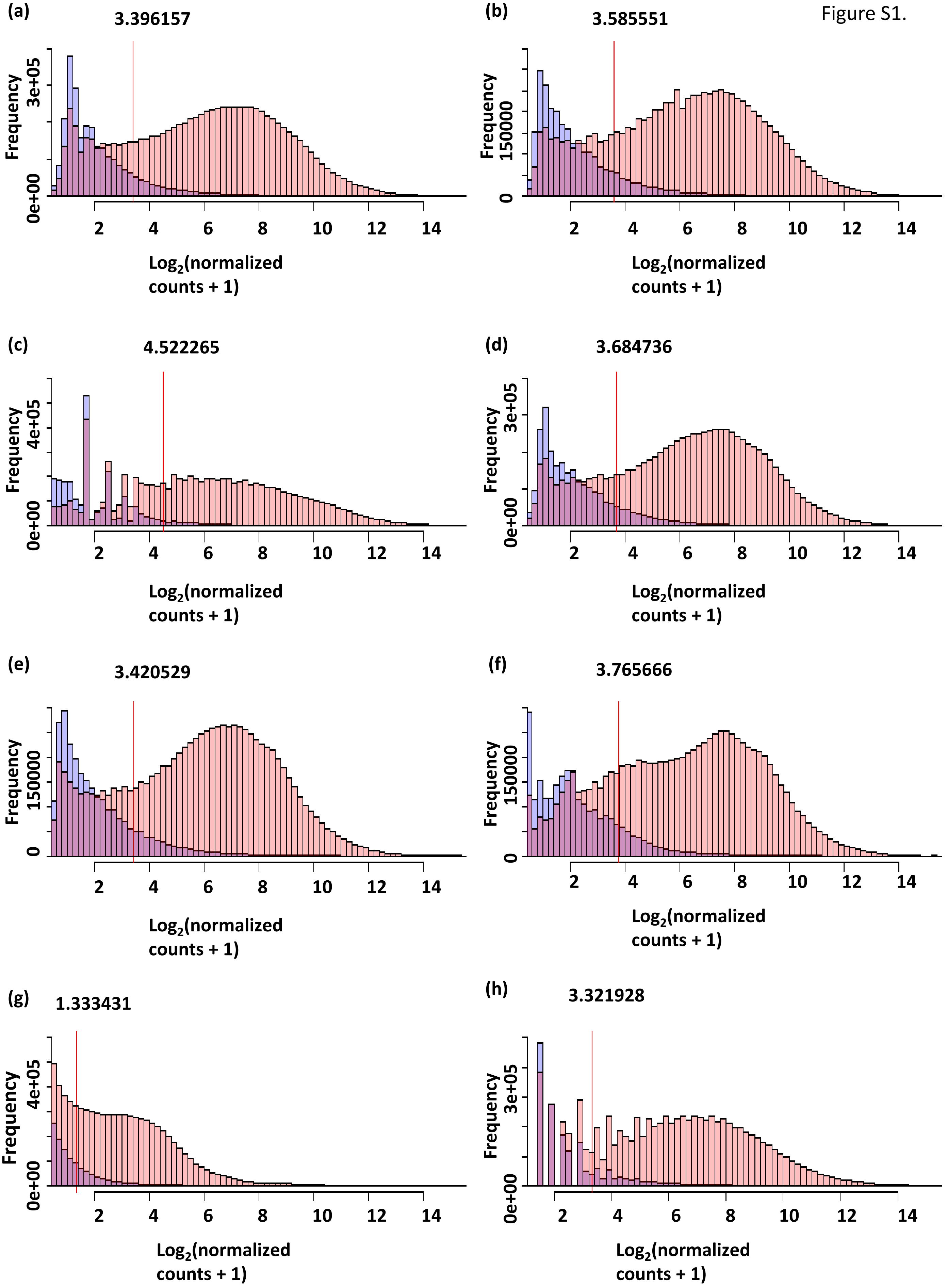

(a)
3.396157
Frequency
0e+00
3e+05
2
4
6
8
10
12
14
Log2(normalized counts + 1)
(b)
3.585551
Frequency
0
150000
2
4
6
8
10
12
14
Log2(normalized counts + 1)
(c)
4.522265
Frequency
0e+00
4e+05
2
4
6
8
10
12
14
Log2(normalized counts + 1)
(d)
3.684736
Frequency
0e+00
3e+05
2
4
6
8
10
12
14
Log2(normalized counts + 1)
(e)
3.420529
Frequency
0
150000
2
4
6
8
10
12
14
Log2(normalized counts + 1)
(f)
3.765666
Frequency
0
150000
2
4
6
8
10
12
14
Log2(normalized counts + 1)
(g)
1.333431
Frequency
0e+00
4e+05
2
4
6
8
10
12
14
Log2(normalized counts + 1)
(h)
3.321928
Frequency
0e+00
3e+05
2
4
6
8
10
12
14
Log2(normalized counts + 1)

Figure S2.

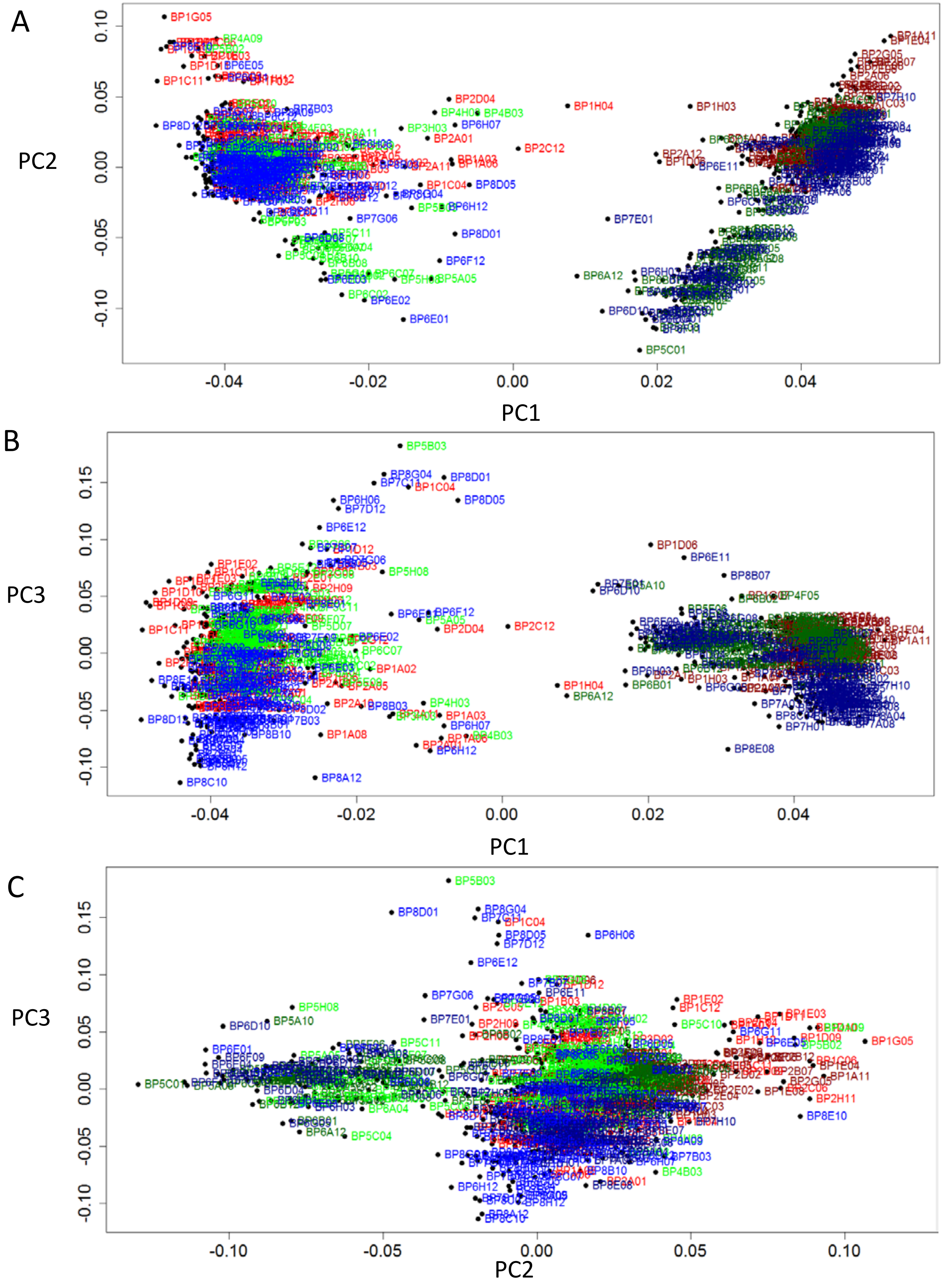
A
PC2
0.10
0.05
0.00
-0.05
-0.10
-0.04
-0.02
0.00
0.02
0.04
PC1
B
PC3
0.15
0.10
0.05
0.00
-0.05
-0.10
-0.04
-0.02
0.00
0.02
0.04
PC1
C
PC3
0.15
0.10
0.05
0.00
-0.05
-0.10
-0.10
-0.05
0.00
0.05
0.10
PC2

Figure S2. (Continued)

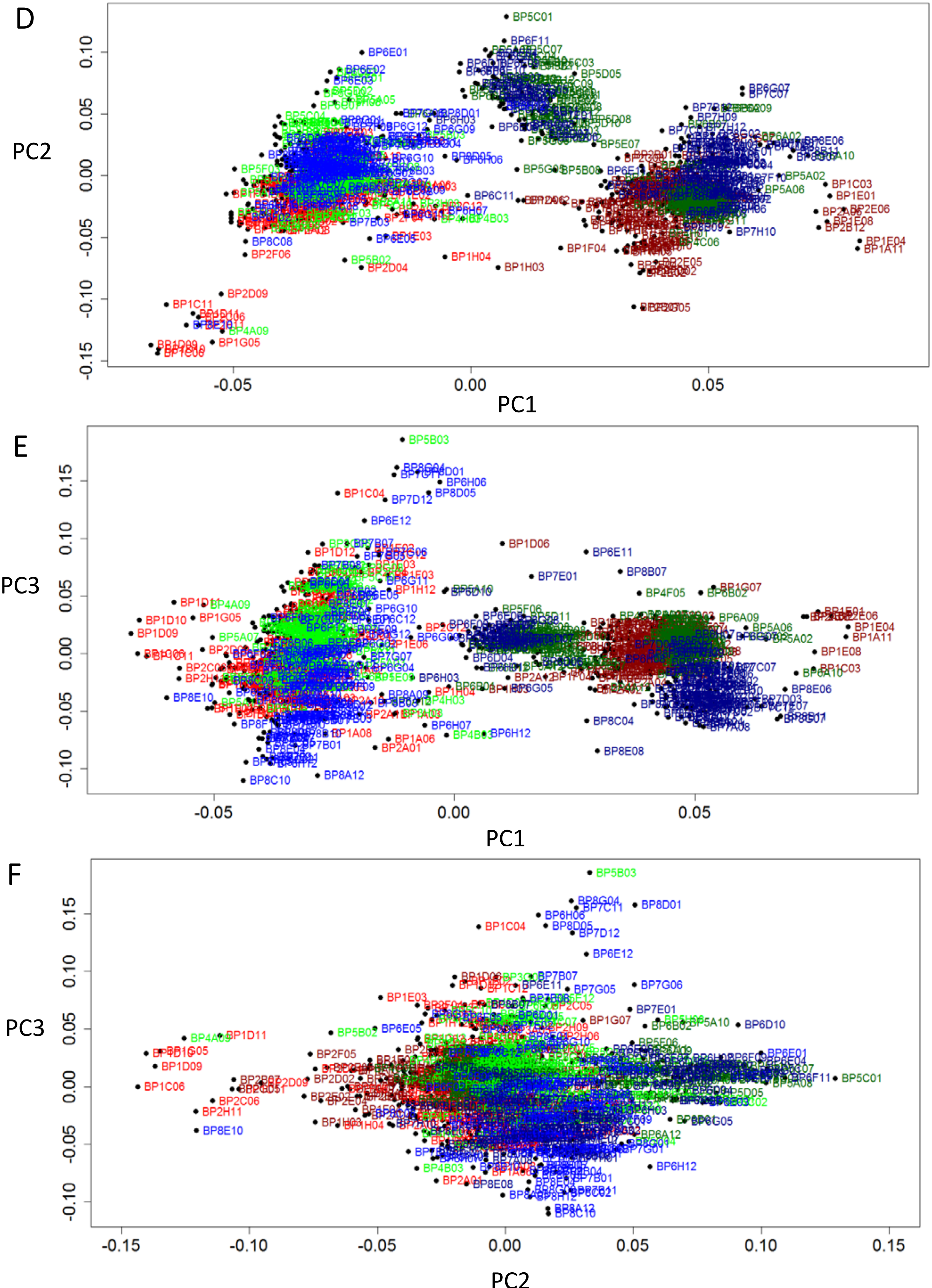

Figure S3.

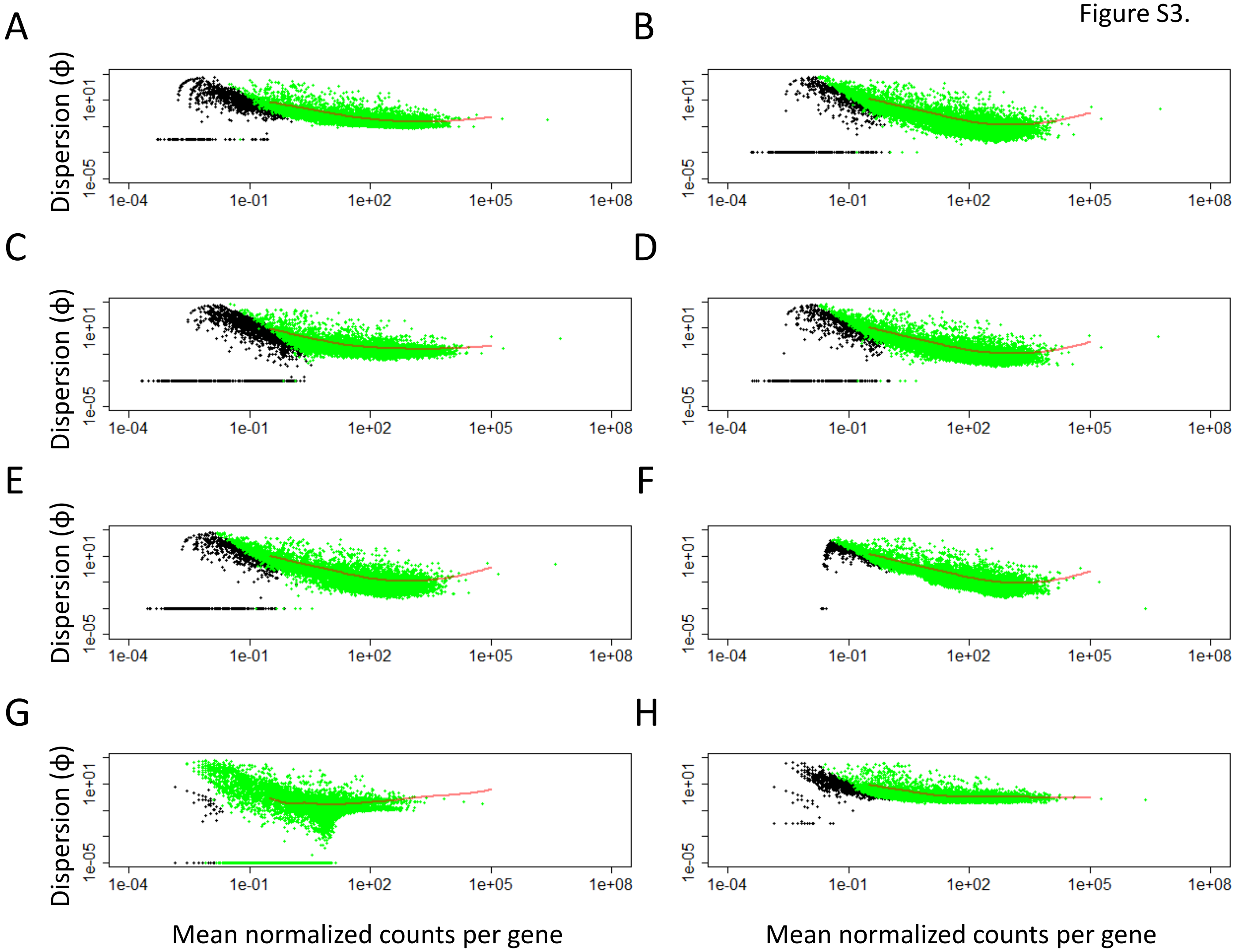
A
B
C
D
E
F
G
H
Dispersion (Φ)
1e+01
1e-05
1e-04
1e-01
1e+02
1e+05
1e+08
Mean normalized counts per gene
Mean normalized counts per gene

Figure S4.

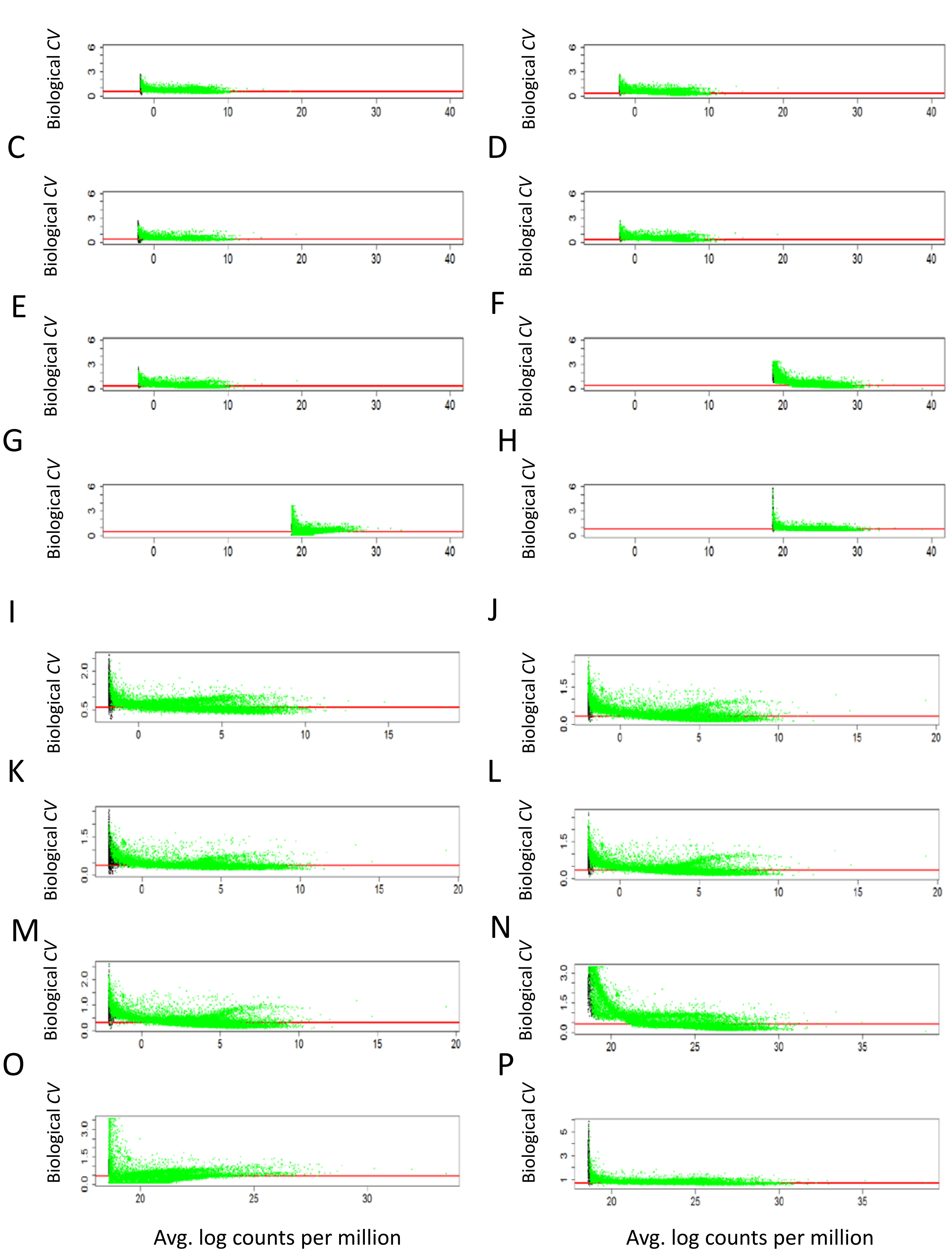
A
B
C
D
E
F
G
H
I
J
K
L
M
N
O
P
Biological CV
Avg. log counts per million
Avg. log counts per million

Figure S5.

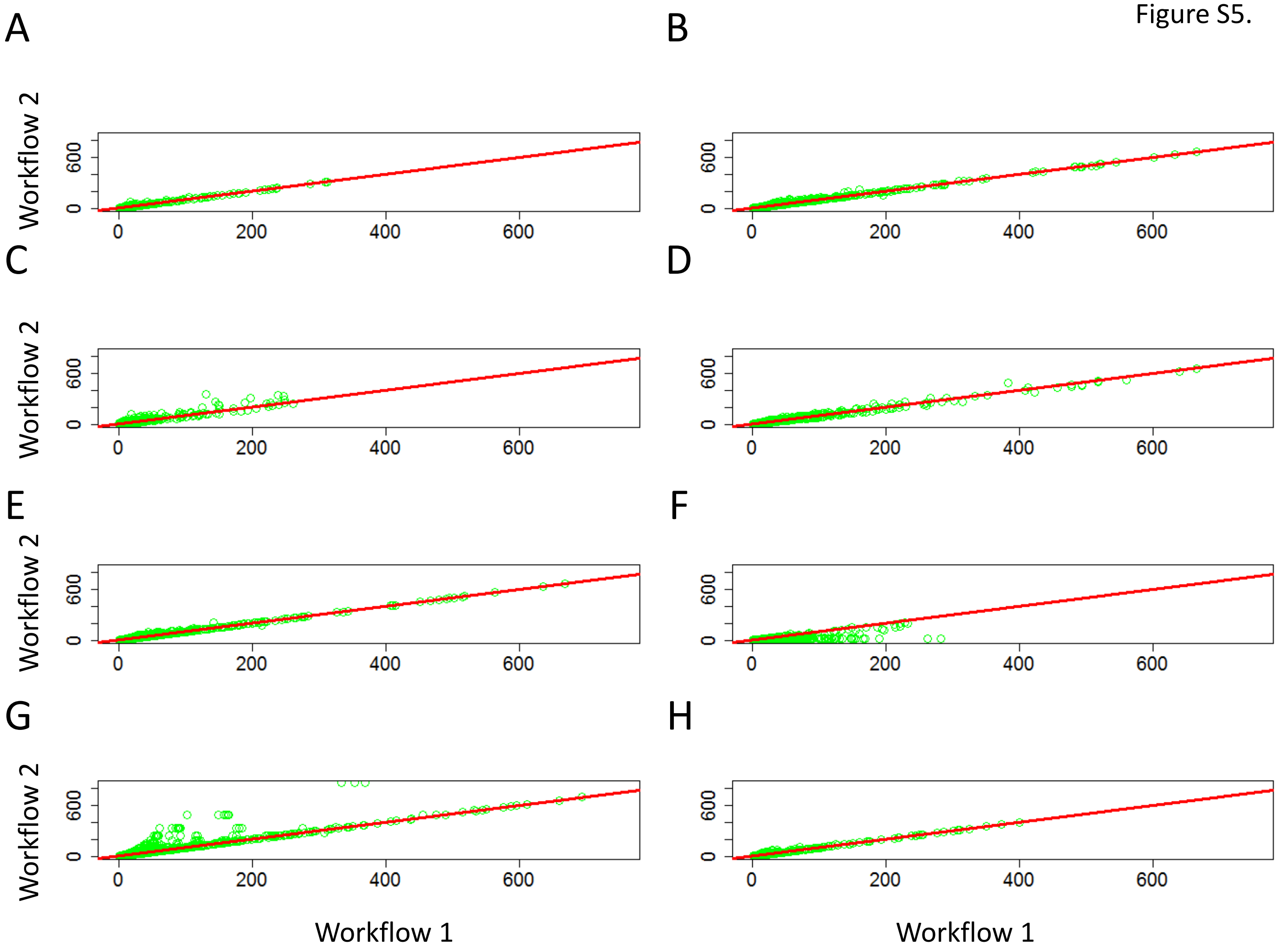

Figure S6.

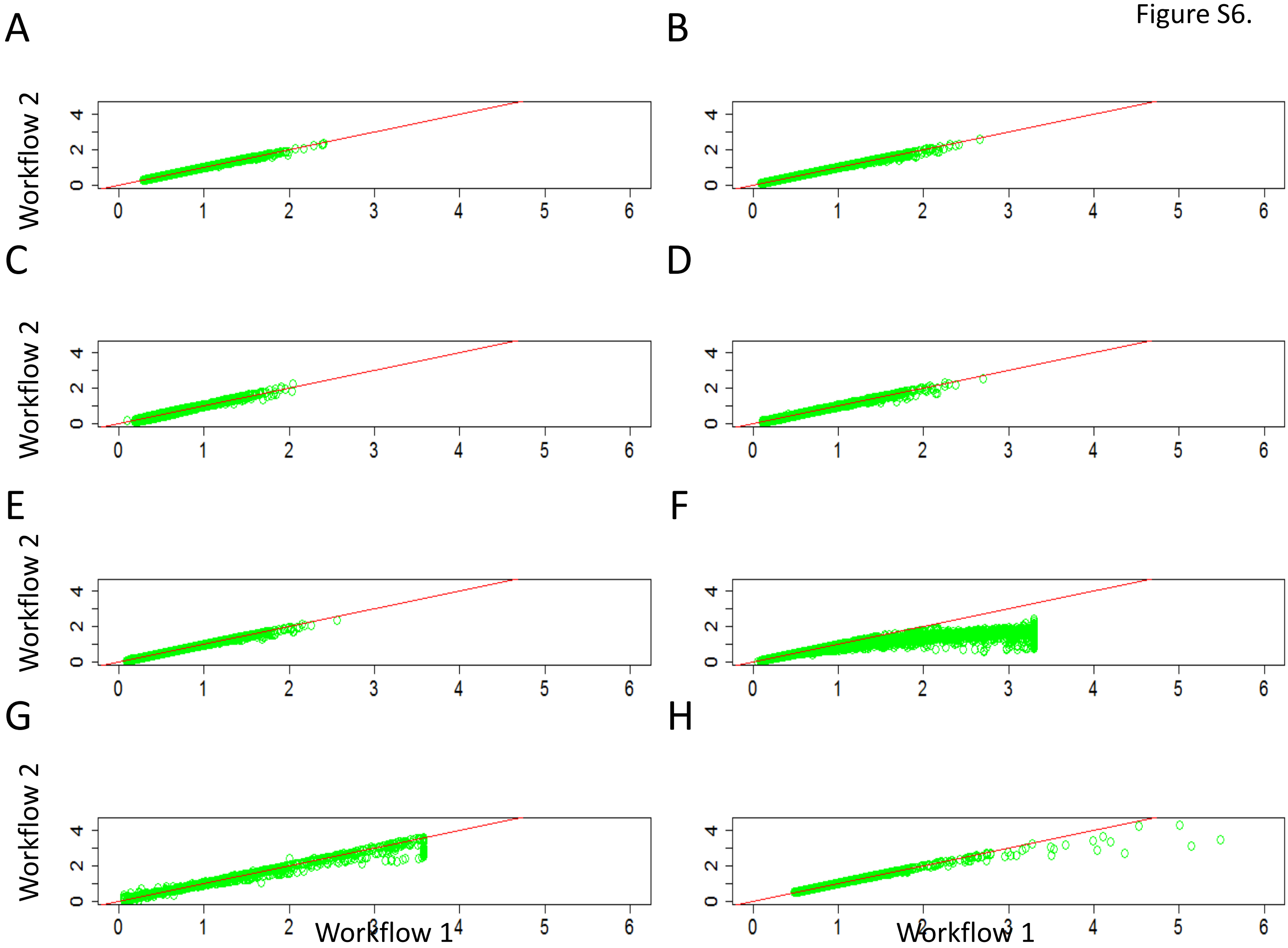

Figure S7.

A

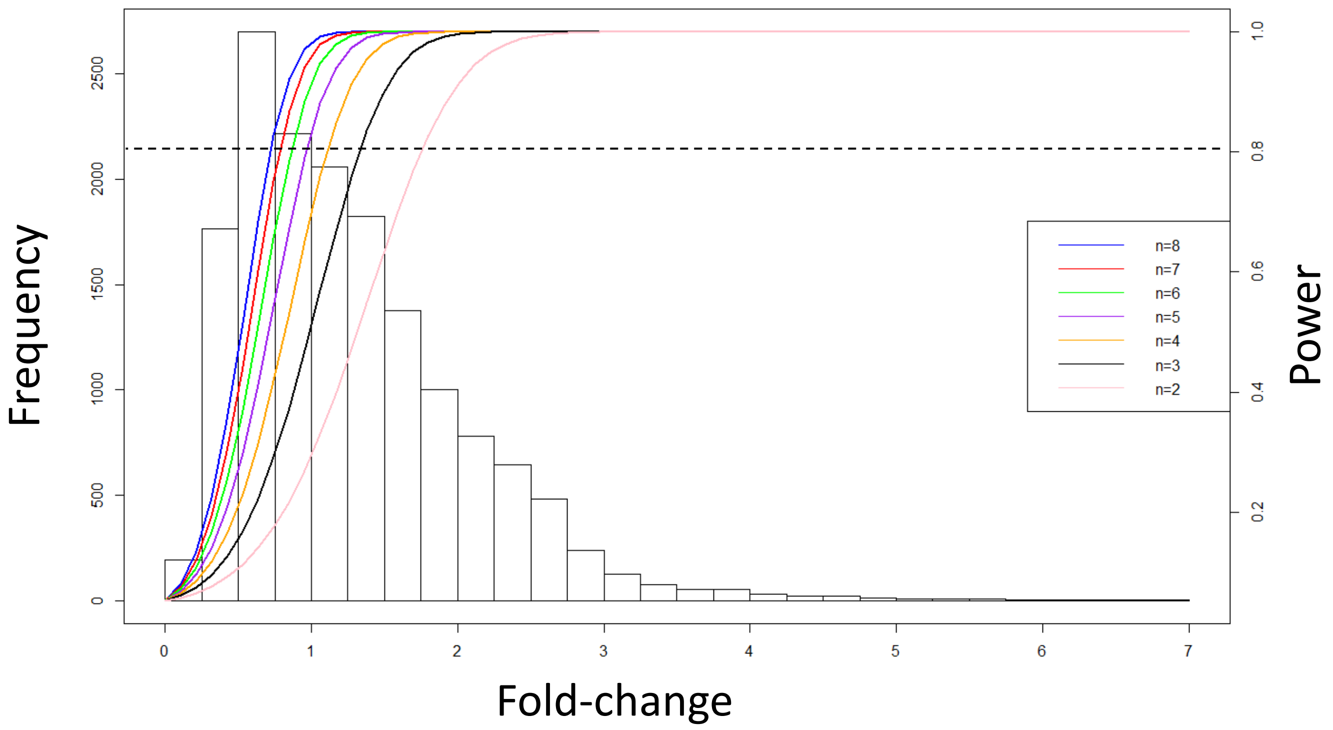

Frequency
Power
n=8
n=7
n=6
n=5
n=4
n=3
n=2
Fold-change

B

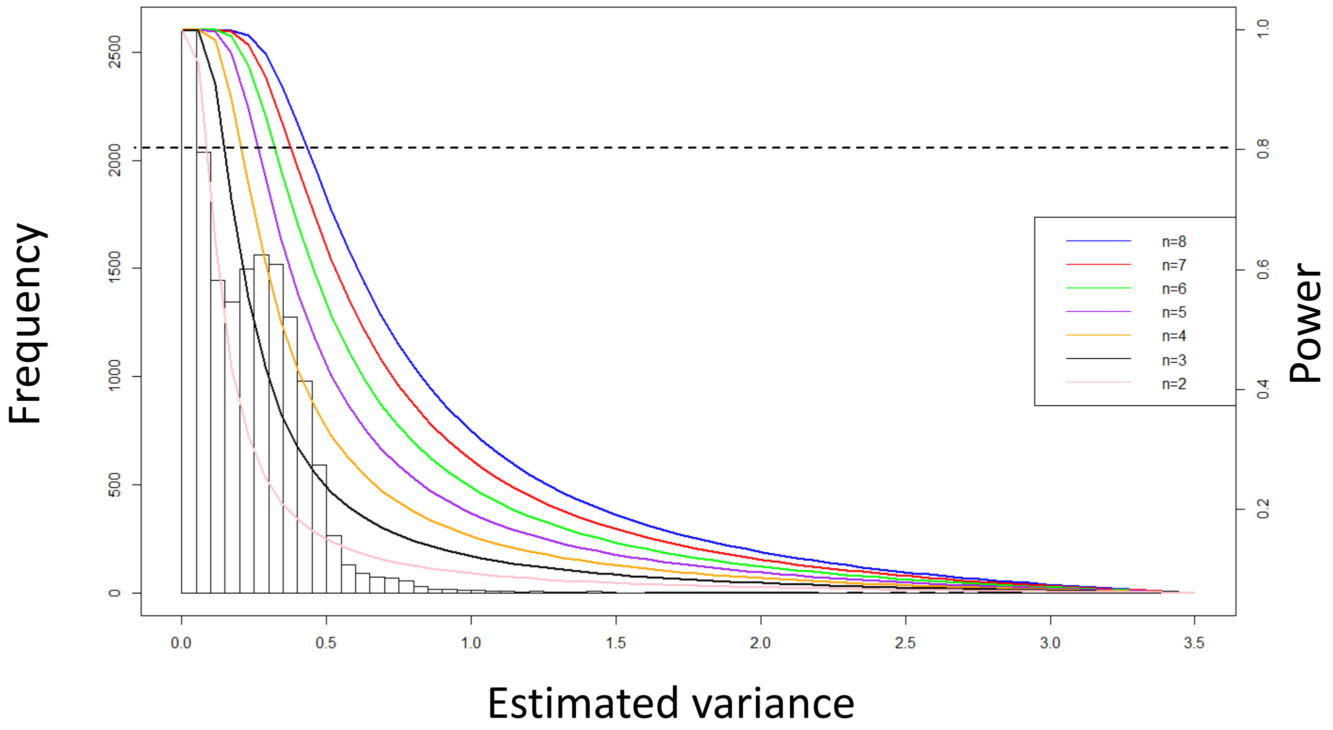

Frequency
Power
n=8
n=7
n=6
n=5
n=4
n=3
n=2
Estimated variance

Figure S8.

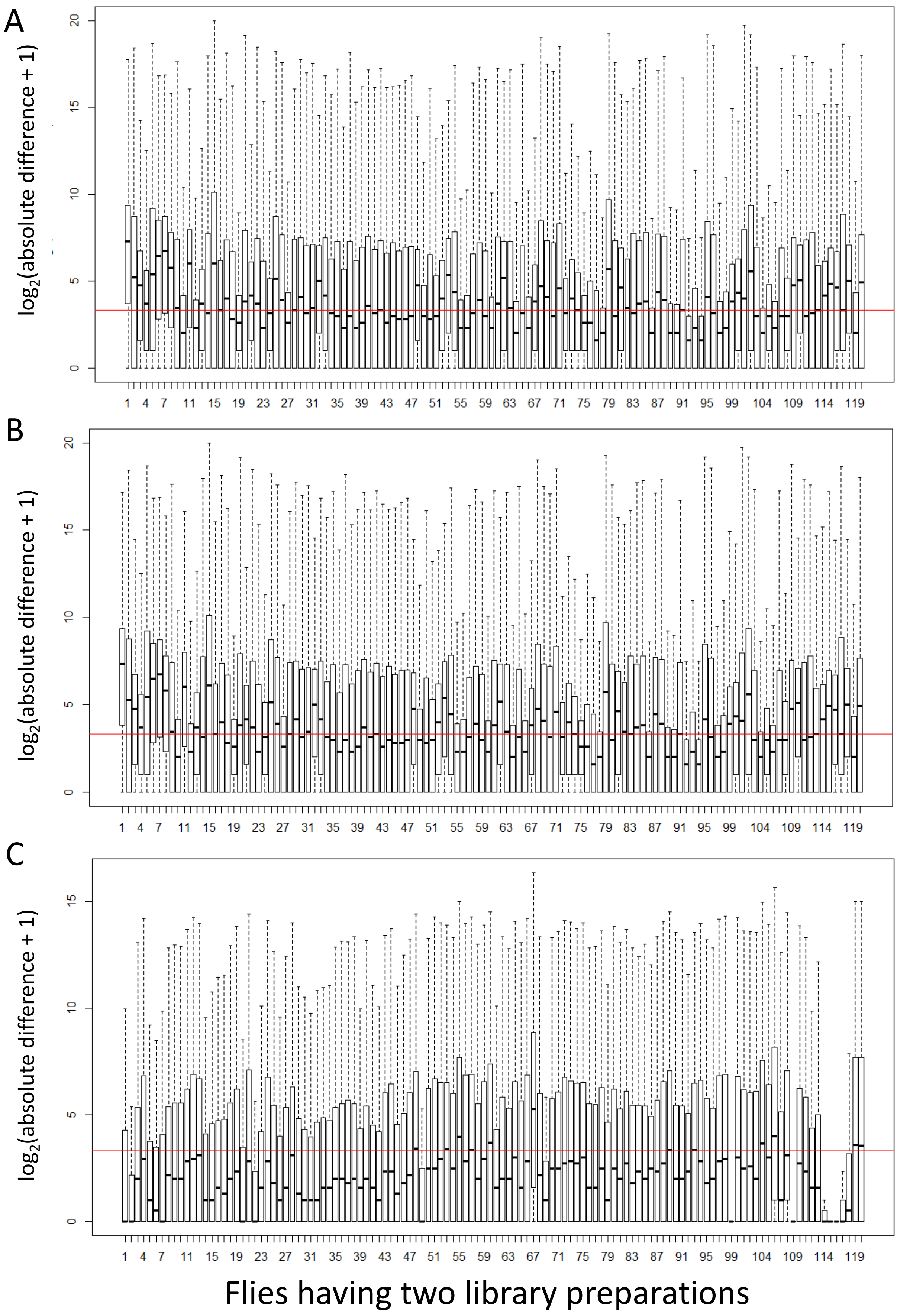
A
B
C
$\log_2$(absolute difference + 1)
Flies having two library preparations

Table S1. Comparison of analyses with and without non-protein-coding genes. Numbers of differentially expressed genes called by *DESeq* using the Workflow 1 generalized linear model with DESeq- and TMM-normalized read count data.

Factor\Method	DESeq normalization			TMM normalization		
	**All Genes (15,695)**	**Non-protein-coding genes removed (13,318)**	**Overlap (%)**	**All Genes (15,692)**	**Non-protein-coding genes removed (13,313)**	**Overlap (%)**
Genotype (G)	10464	9183	9095 (86.9)	11425	9424	9333 (81.6)
Environment (E)	9469	8727	8652 (91.4)	9847	8852	8283 (84.1)
Sex (S)	14866	12733	12716 (85.5)	14850	12750	12677 (85.4)
G x E	3463	3432	3350 (96.7)	3158	3428	3030 (95.9)
G x S	7163	6481	6401 (89.4)	8084	6615	6352 (78.6)
E x S	2725	2737	2592 (95.1)	3234	3009	2367 (73.2)
G x E x S	3611	3544	3424 (94.8)	3501	3515	3140 (89.7)

Table S2. Percentage overlap of differentially expressed genes for first-order interaction terms. The table shows the percentage overlap between the first approach to first-order terms in the generalized linear model and approaches 2 and 3. See Methods for additional details. A, Workflow 1 using *DESeq* software; B, Workflow 1 using *edgeR* software; C, Workflow 2 using *DESeq* software; D, Workflow 2 using *edgeR* software. TC, total counts; UQ, upper quartile; Med, median; TMM, trimmed mean of M-values; DESeq, DESeq normalization method; Q, quantile; RPKM, reads per kilobase per million mapped; RC, un-normalized count data.

A.

**Factor\Method**	**TC**	**UQ**	**Med**	**TMM**	**DESeq**	**Q**	**RPKM**	**RC**	**Approach**
G x E	100	100	100	100	100	100	100	100	2
G x E	78	79	69	82	82	79	65	60	3
G x S	99	99	95	97	99	98	99	100	2
G x S	99	99	99	99	99	99	98	99	3
E x S	97	93	98	95	93	94	96	91	2
E x S	100	100	100	100	100	100	100	100	3

B

**Factor\Method**	**TC**	**UQ**	**Med**	**TMM**	**DESeq**	**Q**	**RPKM**	**RC**	**Approach**
G x E	100	100	100	100	100	100	100	100	2
G x E	86	92	94	93	92	84	85	76	3
G x S	98	99	100	100	99	99	99	100	2
G x S	99	100	100	100	100	100	98	100	3
E x S	97	93	98	95	92	95	97	80	2
E x S	100	100	100	100	100	100	100	100	3

C

Factor\Method	TC	UQ	Med	TMM	DESeq	Q	RPKM	RC	Approach
G x E	100	100	100	100	100	100	100	100	2
G x E	78	79	73	81	82	82	65	60	3
G x S	99	99	96	98	99	99	99	100	2
G x S	99	99	99	99	99	99	98	99	3
E x S	97	93	98	94	92	97	96	92	2
E x S	100	100	100	100	100	100	100	100	3

D

Factor\Method	TC	UQ	Med	TMM	DESeq	Q	RPKM	RC	Approach
G x E	100	100	100	100	100	100	100	100	2
G x E	86	92	94	93	92	84	85	76	3
G x S	98	99	100	99	99	99	98	100	2
G x S	99	99	100	100	100	100	98	100	3
E x S	97	93	98	93	92	96	97	80	2
E x S	100	100	100	100	100	100	100	100	3

Table S3. Comparison of Workflow 1 and Workflow 3 analyses for DESeq-normalized data. Numbers of differentially expressed genes called by *DESeq* using the generalized linear model with DESeq-normalized read count data.   Total number of genes analyzed are given in parentheses.

**Factor\Method**	**Workflow 1 (16,649)**	**Workflow 3 (17,142)**	**Overlap (%)**
Genotype (G)	10464	10474	99.6
Environment (E)	9469	9444	99.5
Sex (S)	14866	15285	99.9
G x E	3463	3450	99.6
G x S	7163	7125	99.5
E x S	2725	2674	98.1
G x E x S	3611	3587	99.3
