# Supplementary material for: Comparison of normalization and differential expression analyses using RNA-Seq data from 726 individual Drosophila melanogaster
Source: BMC Genomics. 2016 Jan 5;17:28. doi: 10.1186/s12864-015-2353-z (PMC4702322; doi:10.1186/s12864-015-2353-z)
Supplement: Additional file 2: — Comparison of normalization methods for each fly. Boxplots show the differences in the read count distribution for each fly. Flies are grouped by genotype, sex, and environment. (PDF 437 kb) [file 12864_2015_2353_MOESM2_ESM.pdf]

Line: *RAL-93*

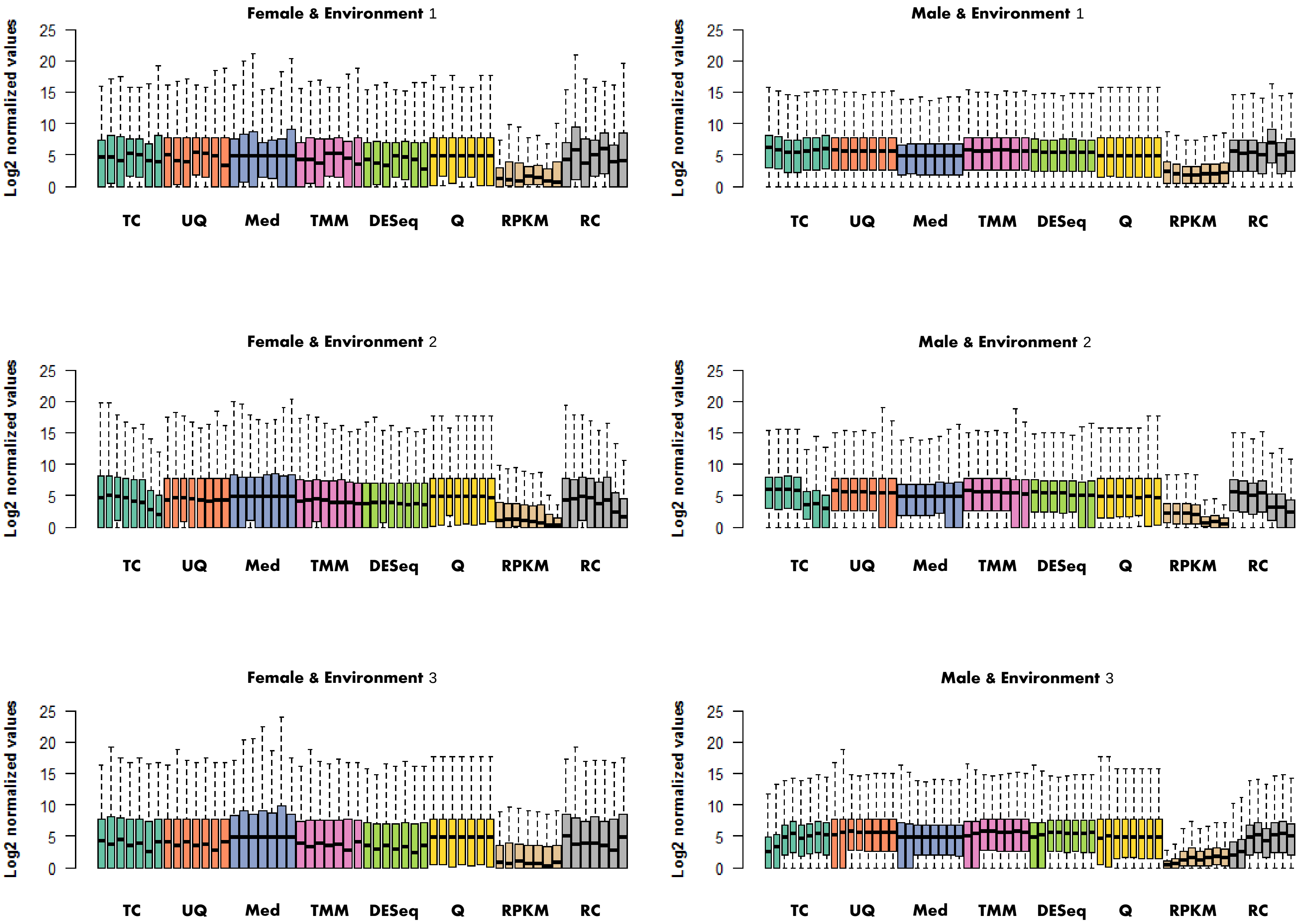
Female & Environment 1
Male & Environment 1
Female & Environment 2
Male & Environment 2
Female & Environment 3
Male & Environment 3
Log2 normalized values
0
5
10
15
20
25
TC
UQ
Med
TMM
DESeq
Q
RPKM
RC

Line: *RAL-229*

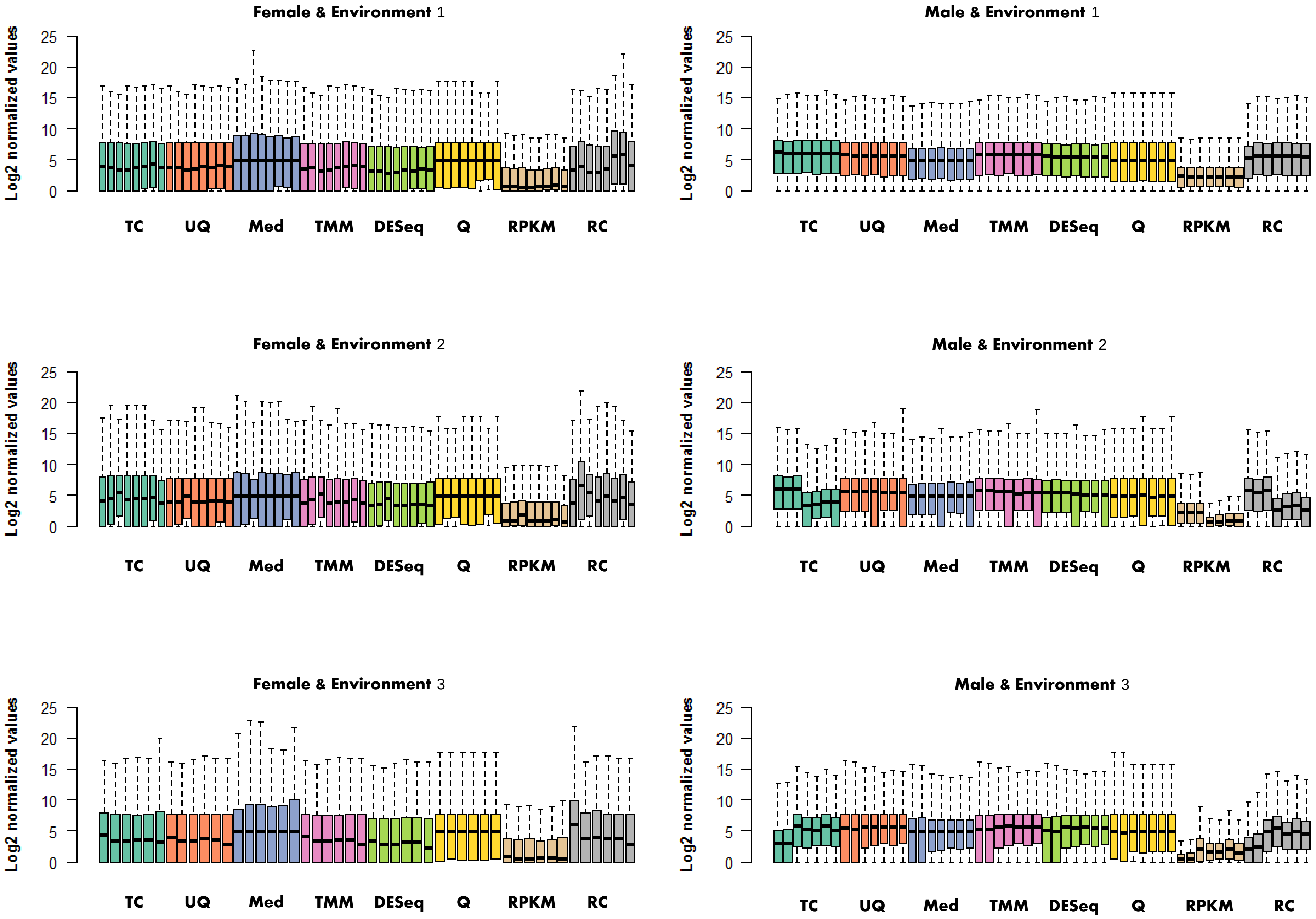
Female & Environment 1
Male & Environment 1
Female & Environment 2
Male & Environment 2
Female & Environment 3
Male & Environment 3
Log2 normalized values
0
5
10
15
20
25
TC
UQ
Med
TMM
DESeq
Q
RPKM
RC

Line: *RAL-320*

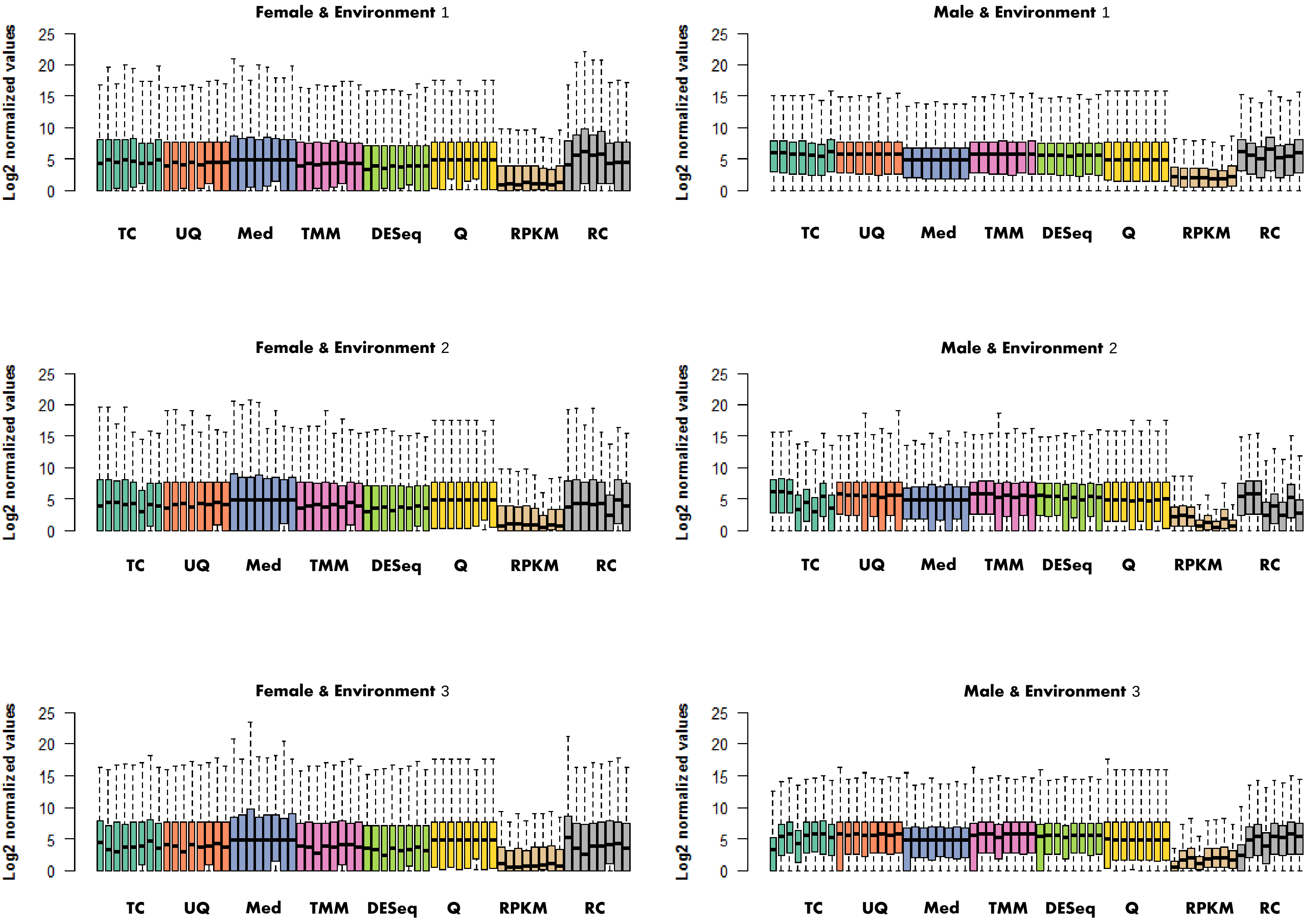
Female & Environment 1
Male & Environment 1
Female & Environment 2
Male & Environment 2
Female & Environment 3
Male & Environment 3
Log2 normalized values
0
5
10
15
20
25
TC
UQ
Med
TMM
DESeq
Q
RPKM
RC

Line: *RAL-352*

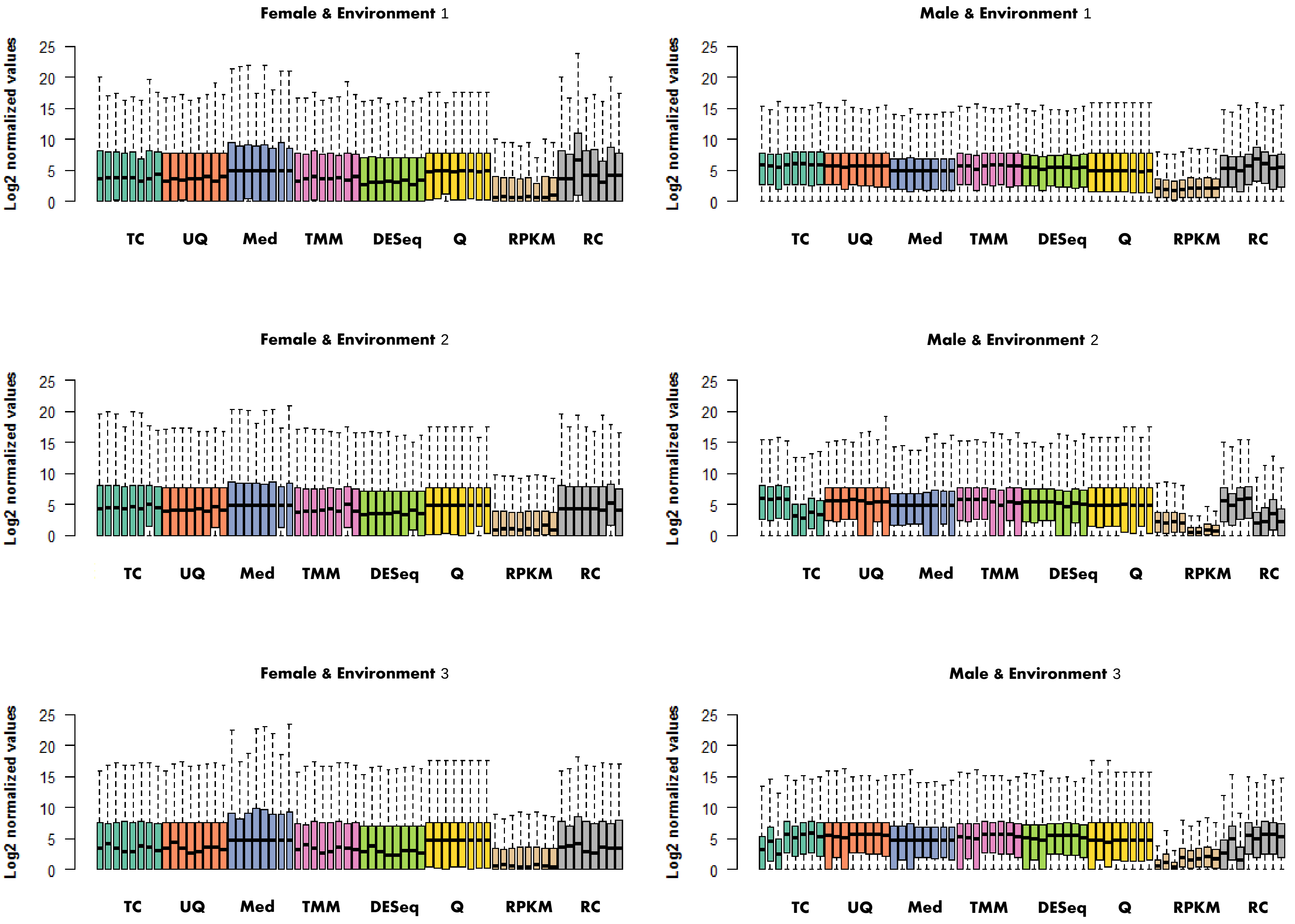
Female & Environment 1
Male & Environment 1
Female & Environment 2
Male & Environment 2
Female & Environment 3
Male & Environment 3
Log2 normalized values
0
5
10
15
20
25
TC
UQ
Med
TMM
DESeq
Q
RPKM
RC

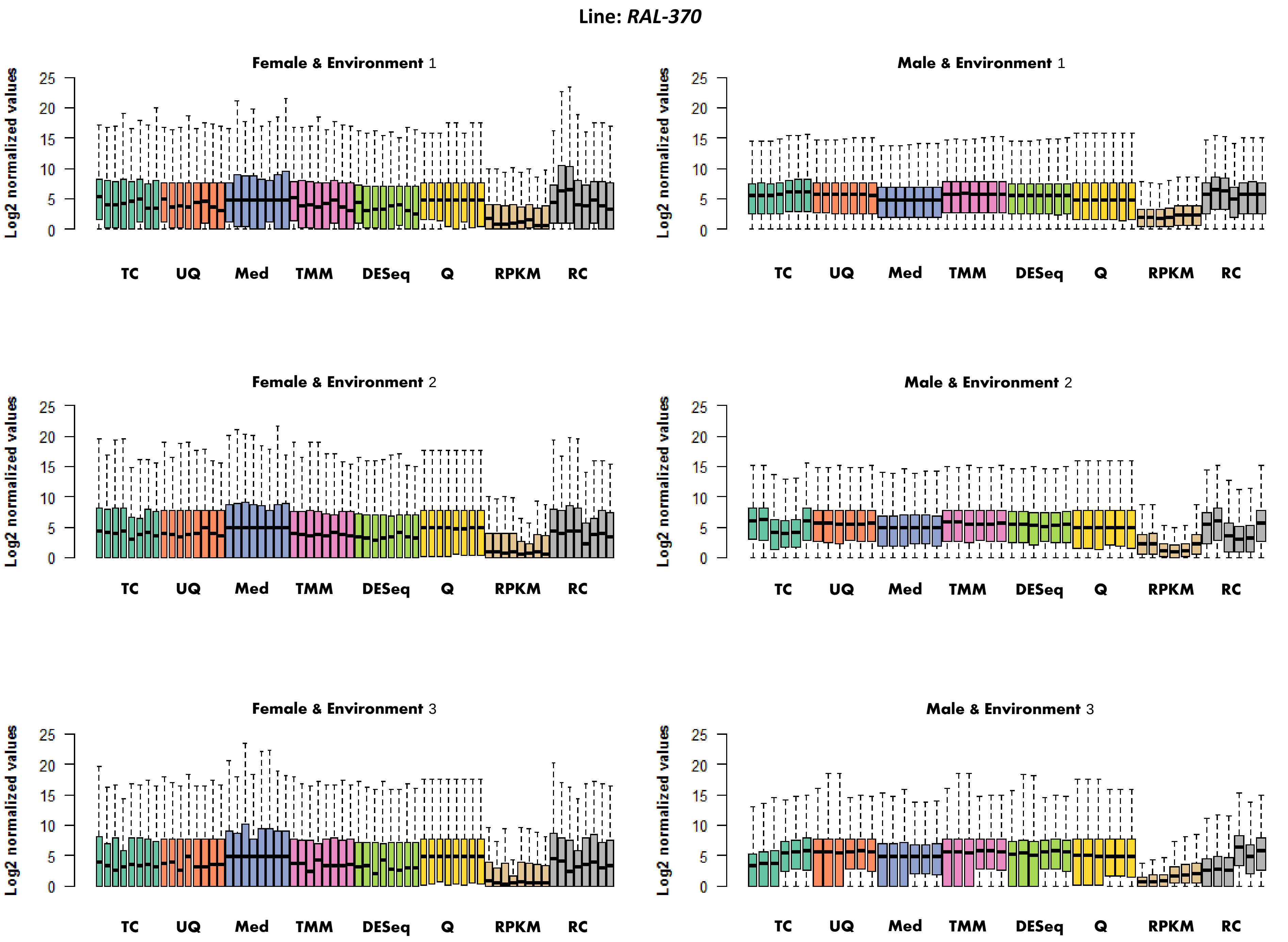
Line: RAL-370
Female & Environment 1
Male & Environment 1
Female & Environment 2
Male & Environment 2
Female & Environment 3
Male & Environment 3
Log2 normalized values
0
5
10
15
20
25
TC
UQ
Med
TMM
DESeq
Q
RPKM
RC

Line: *RAL-563*

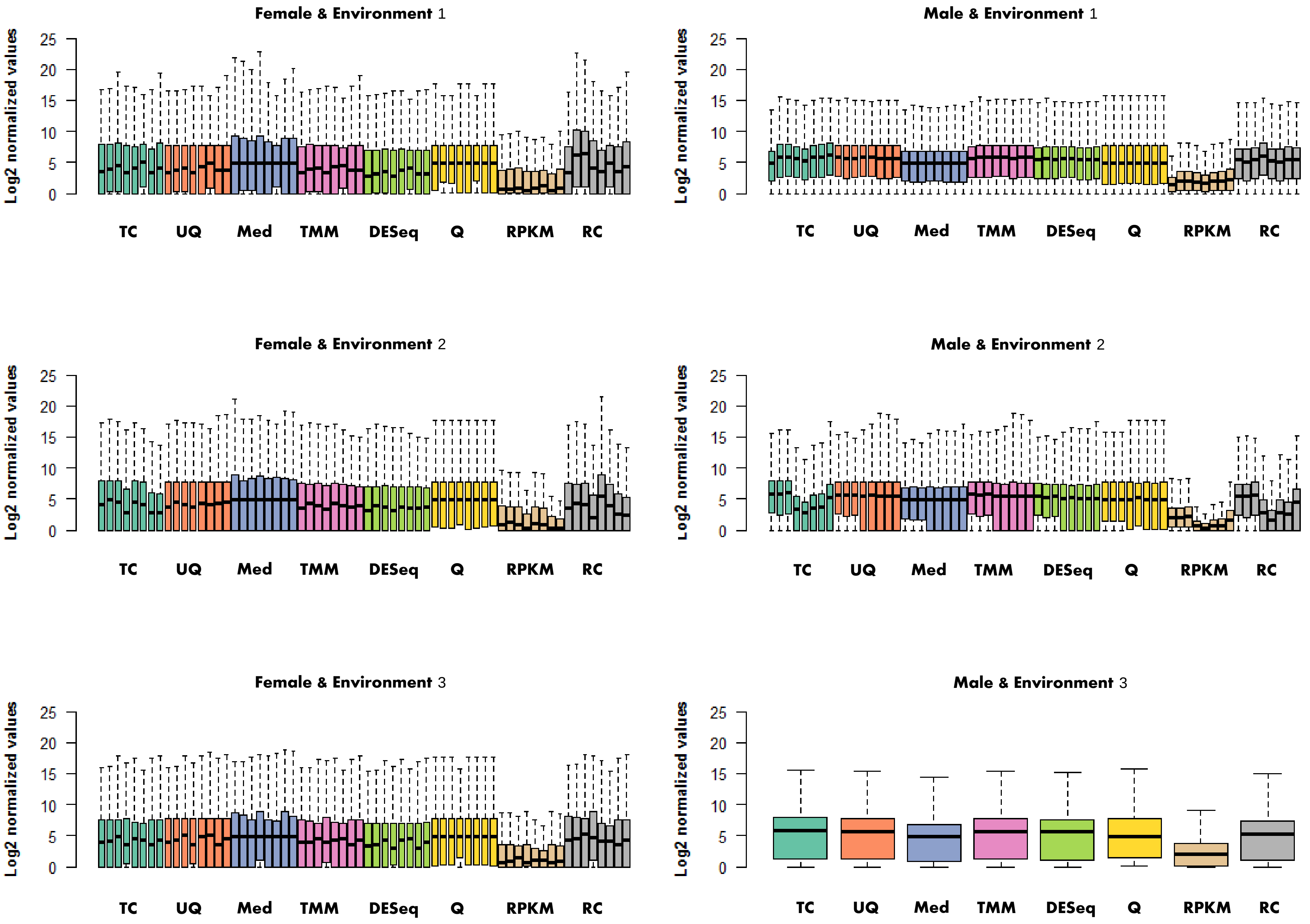
Female & Environment 1
Male & Environment 1
Female & Environment 2
Male & Environment 2
Female & Environment 3
Male & Environment 3
Log2 normalized values
0
5
10
15
20
25
TC
UQ
Med
TMM
DESeq
Q
RPKM
RC

Line: *RAL-630*

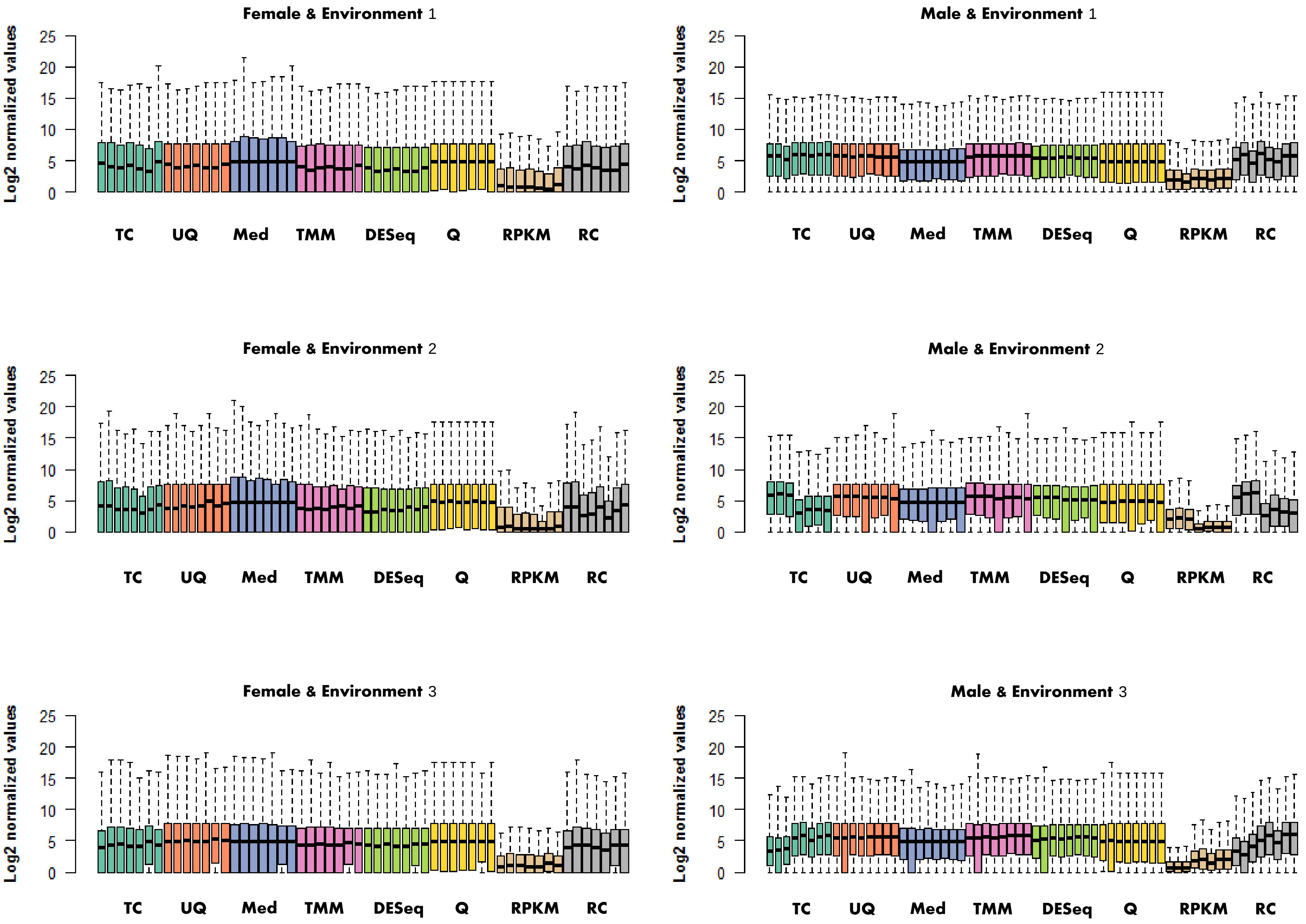
Female & Environment 1
Male & Environment 1
Female & Environment 2
Male & Environment 2
Female & Environment 3
Male & Environment 3
Log2 normalized values
0
5
10
15
20
25
TC
UQ
Med
TMM
DESeq
Q
RPKM
RC

Line: *RAL-703*

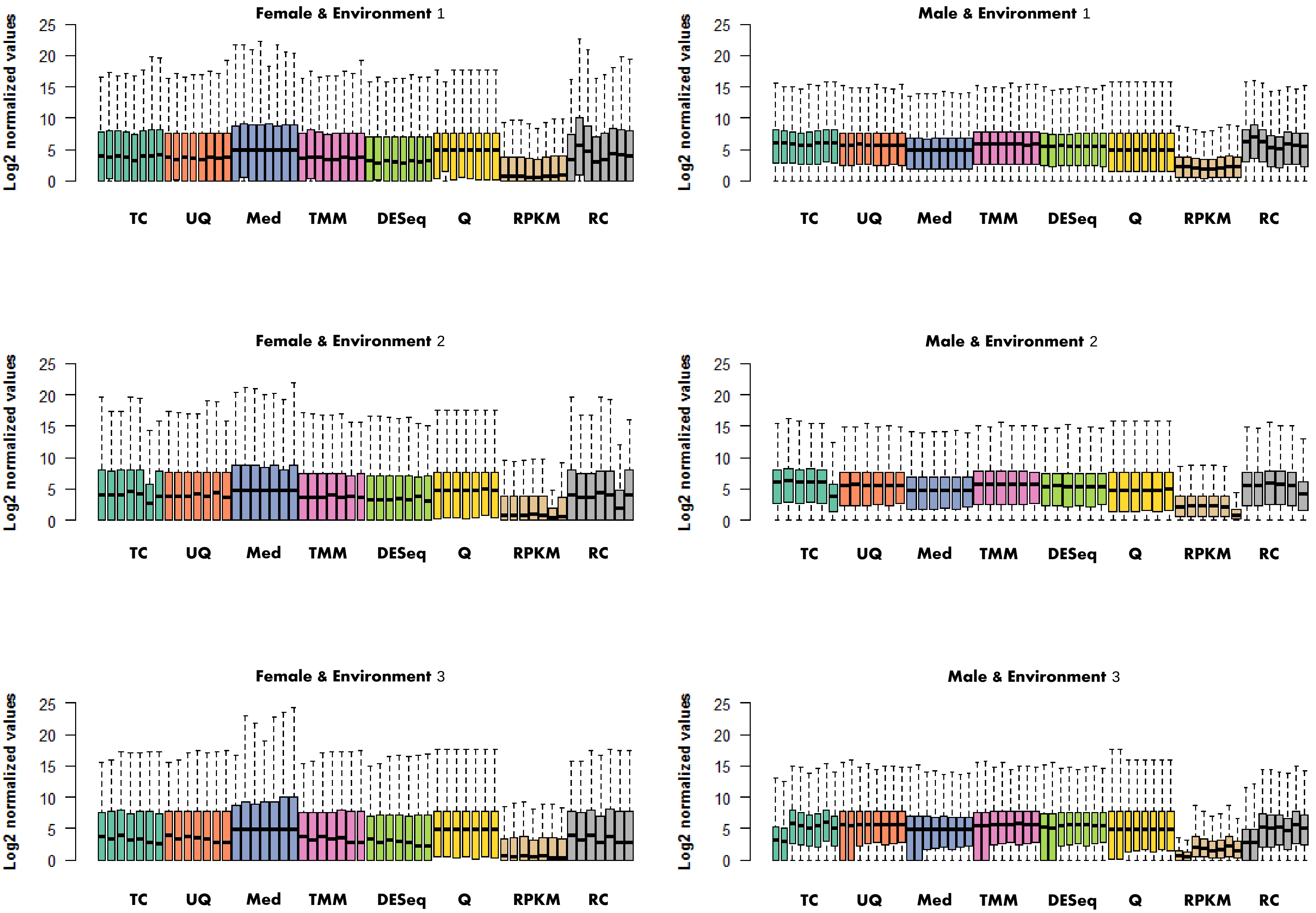
Female & Environment 1
Male & Environment 1
Female & Environment 2
Male & Environment 2
Female & Environment 3
Male & Environment 3
Log2 normalized values
0
5
10
15
20
25
TC
UQ
Med
TMM
DESeq
Q
RPKM
RC

Line: *RAL-761*

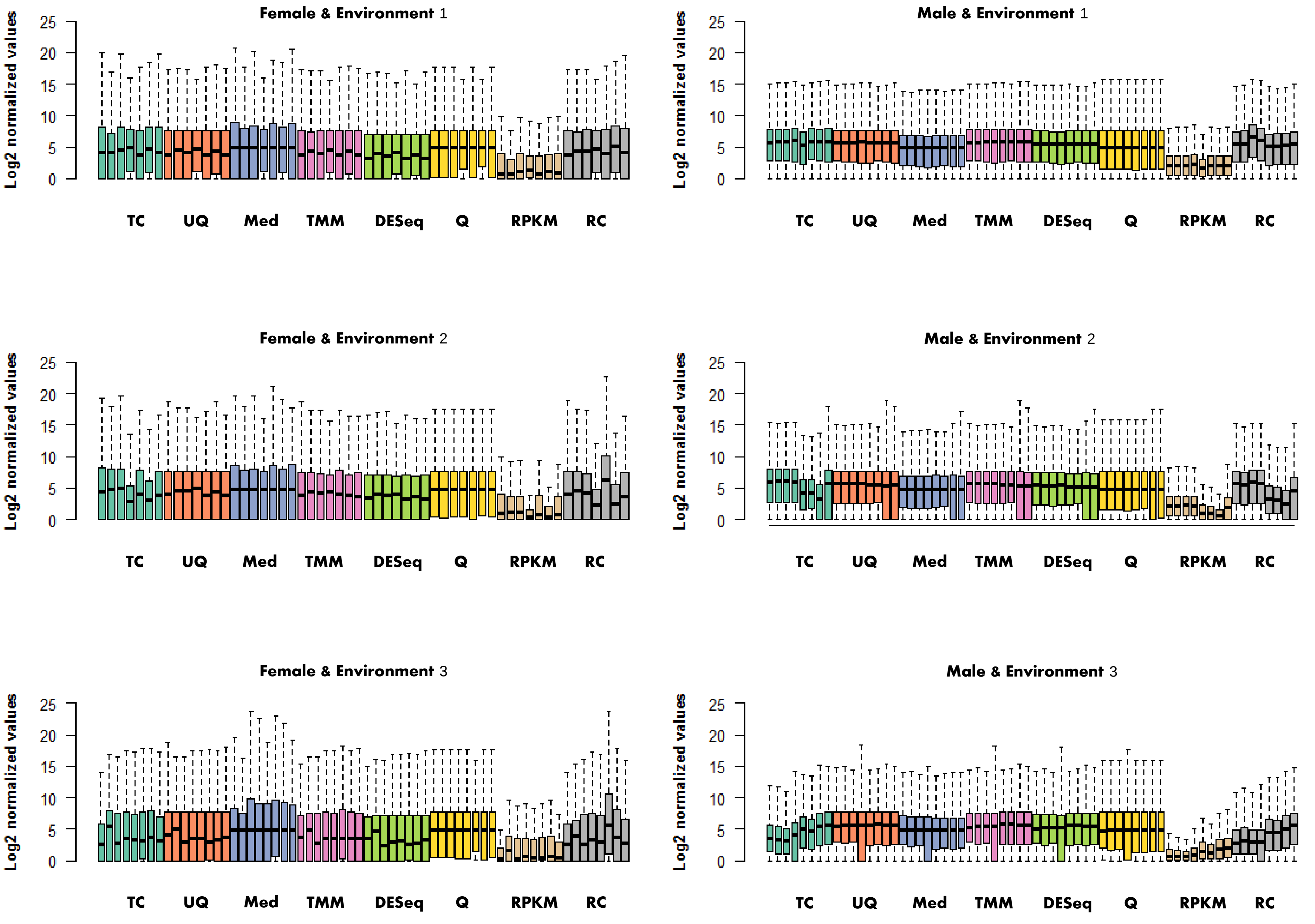
Female & Environment 1
Male & Environment 1
Female & Environment 2
Male & Environment 2
Female & Environment 3
Male & Environment 3
Log2 normalized values
0
5
10
15
20
25
TC
UQ
Med
TMM
DESeq
Q
RPKM
RC

Line: *RAL-787*

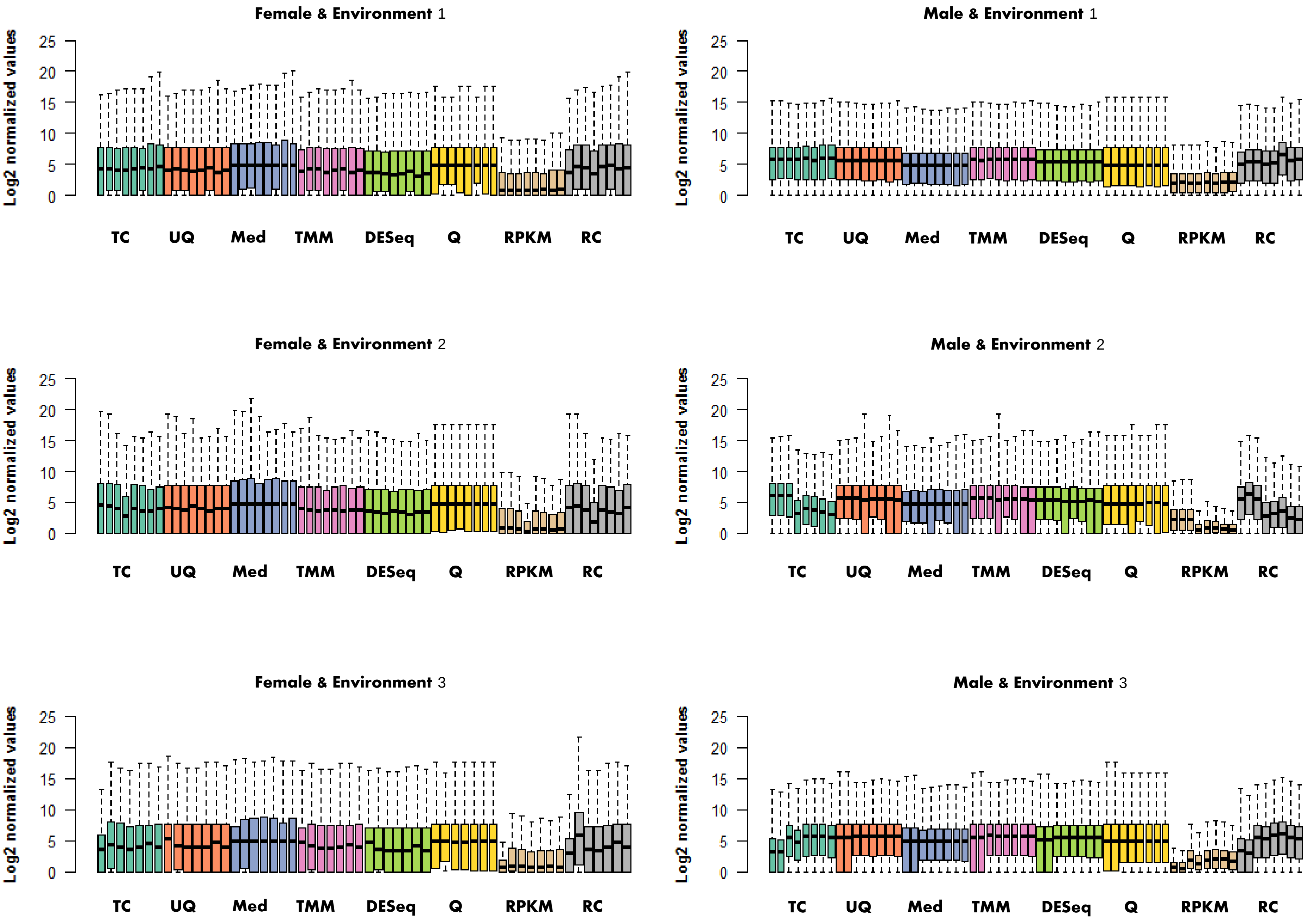
Female & Environment 1
Male & Environment 1
Female & Environment 2
Male & Environment 2
Female & Environment 3
Male & Environment 3
Log2 normalized values
0
5
10
15
20
25
TC
UQ
Med
TMM
DESeq
Q
RPKM
RC

Line: *RAL-790*

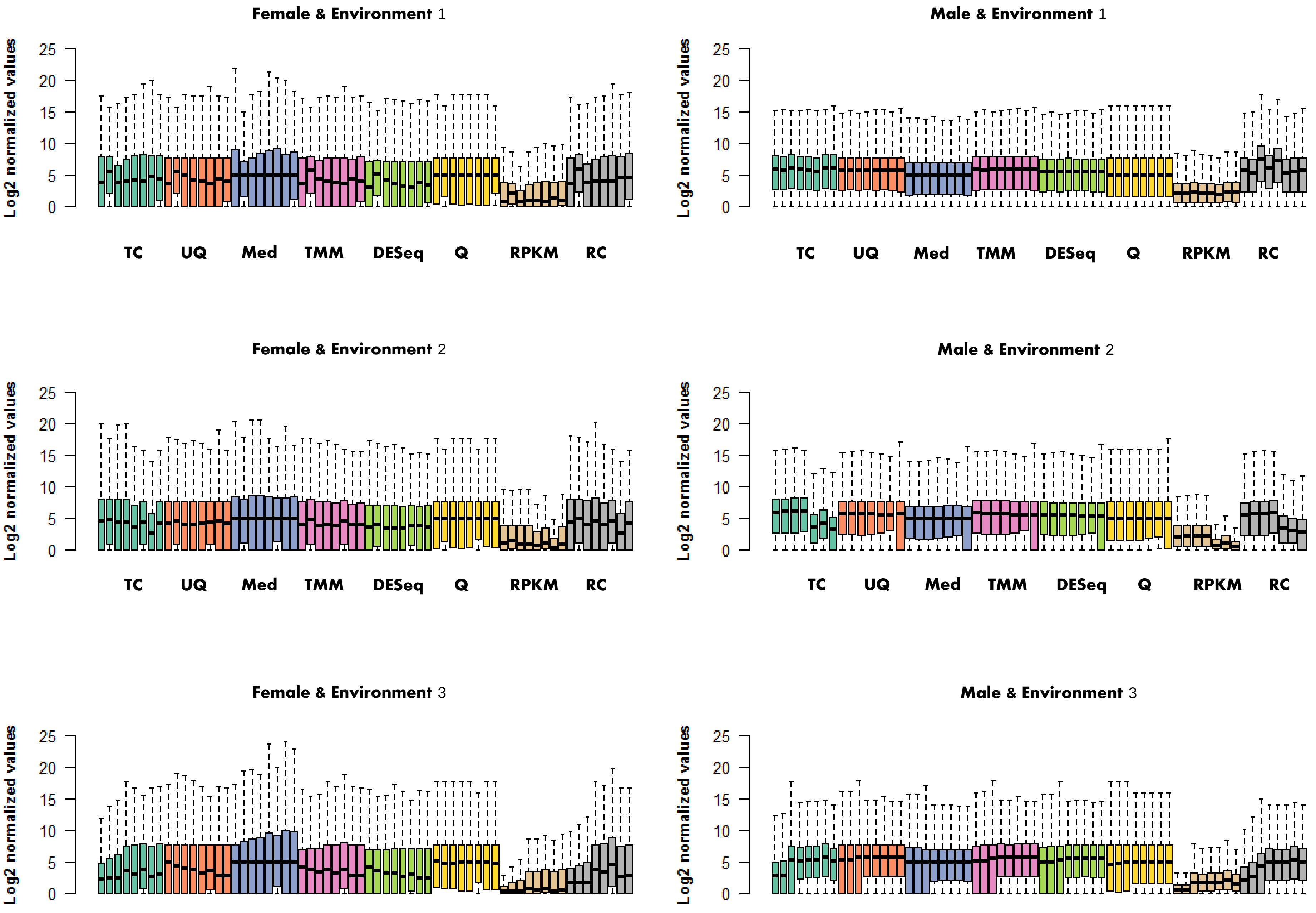
Female & Environment 1
Male & Environment 1
Female & Environment 2
Male & Environment 2
Female & Environment 3
Male & Environment 3
Log2 normalized values
0
5
10
15
20
25
TC
UQ
Med
TMM
DESeq
Q
RPKM
RC

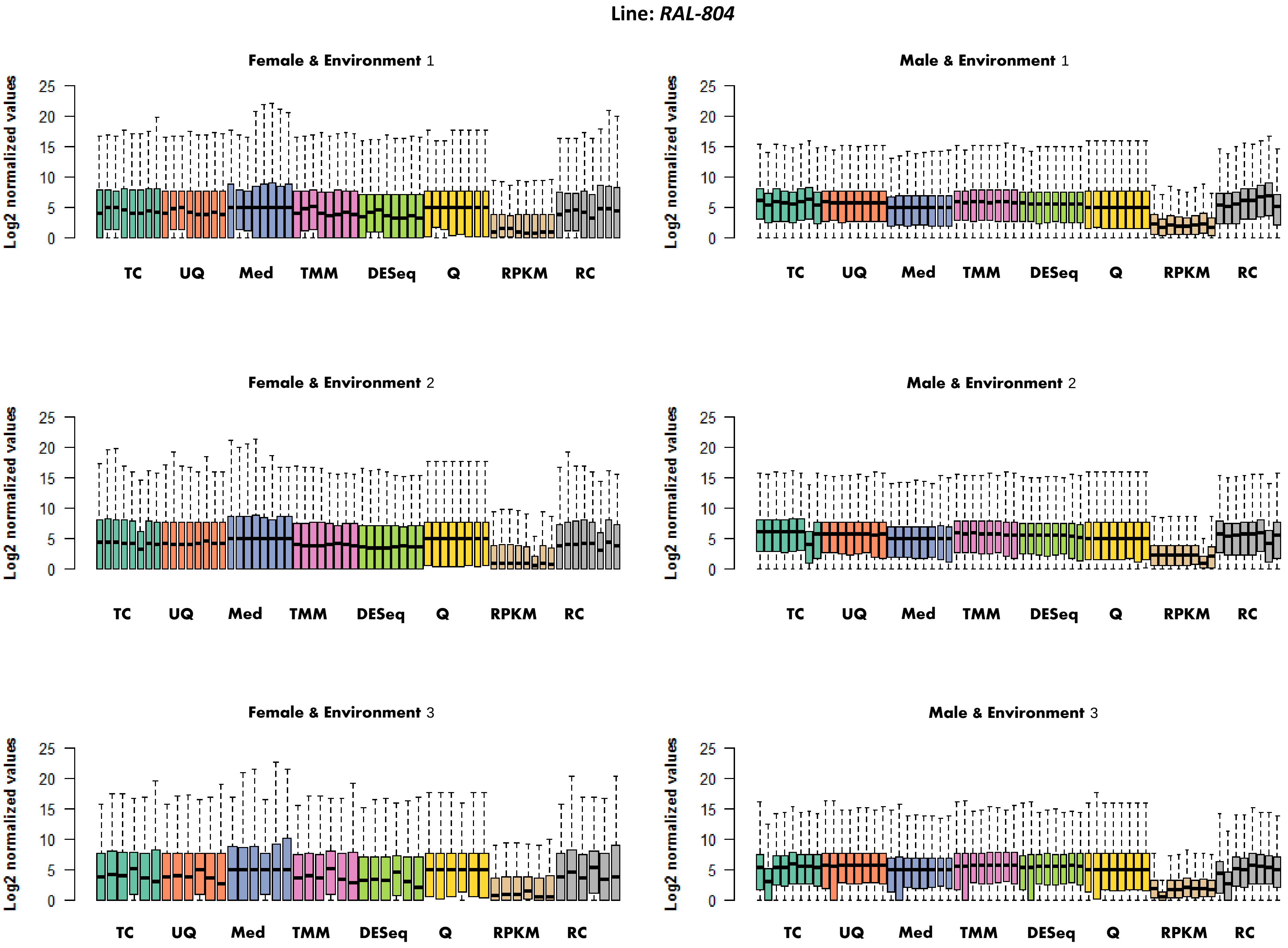
Line: RAL-804
Female & Environment 1
Male & Environment 1
Female & Environment 2
Male & Environment 2
Female & Environment 3
Male & Environment 3
Log2 normalized values
0
5
10
15
20
25
TC
UQ
Med
TMM
DESeq
Q
RPKM
RC

Line: *RAL-812*

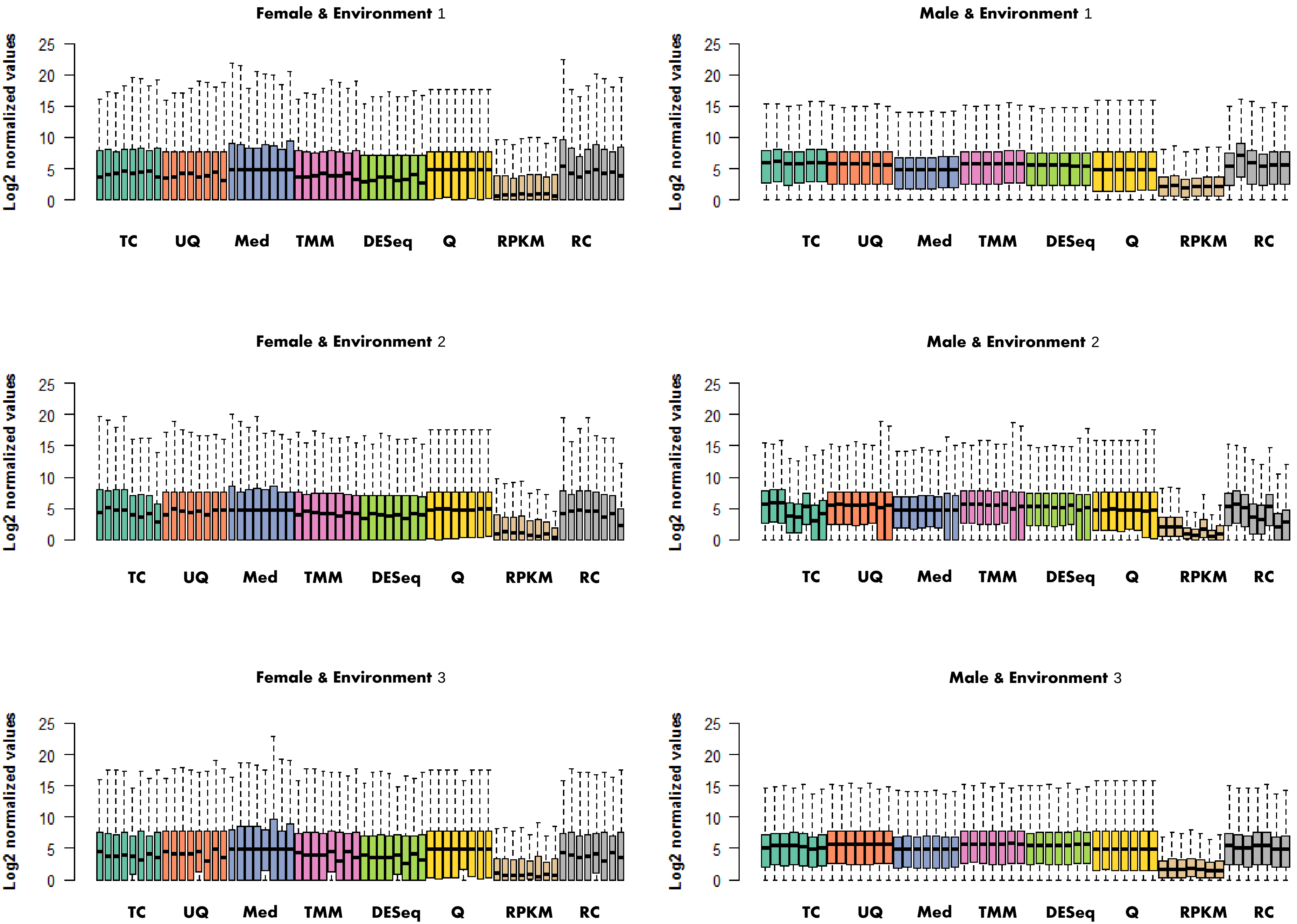

Female & Environment 1
Log2 normalized values
0
5
10
15
20
25
TC
UQ
Med
TMM
DESeq
Q
RPKM
RC
Male & Environment 1
Log2 normalized values
0
5
10
15
20
25
TC
UQ
Med
TMM
DESeq
Q
RPKM
RC
Female & Environment 2
Log2 normalized values
0
5
10
15
20
25
TC
UQ
Med
TMM
DESeq
Q
RPKM
RC
Male & Environment 2
Log2 normalized values
0
5
10
15
20
25
TC
UQ
Med
TMM
DESeq
Q
RPKM
RC
Female & Environment 3
Log2 normalized values
0
5
10
15
20
25
TC
UQ
Med
TMM
DESeq
Q
RPKM
RC
Male & Environment 3
Log2 normalized values
0
5
10
15
20
25
TC
UQ
Med
TMM
DESeq
Q
RPKM
RC

Line: *RAL-822*

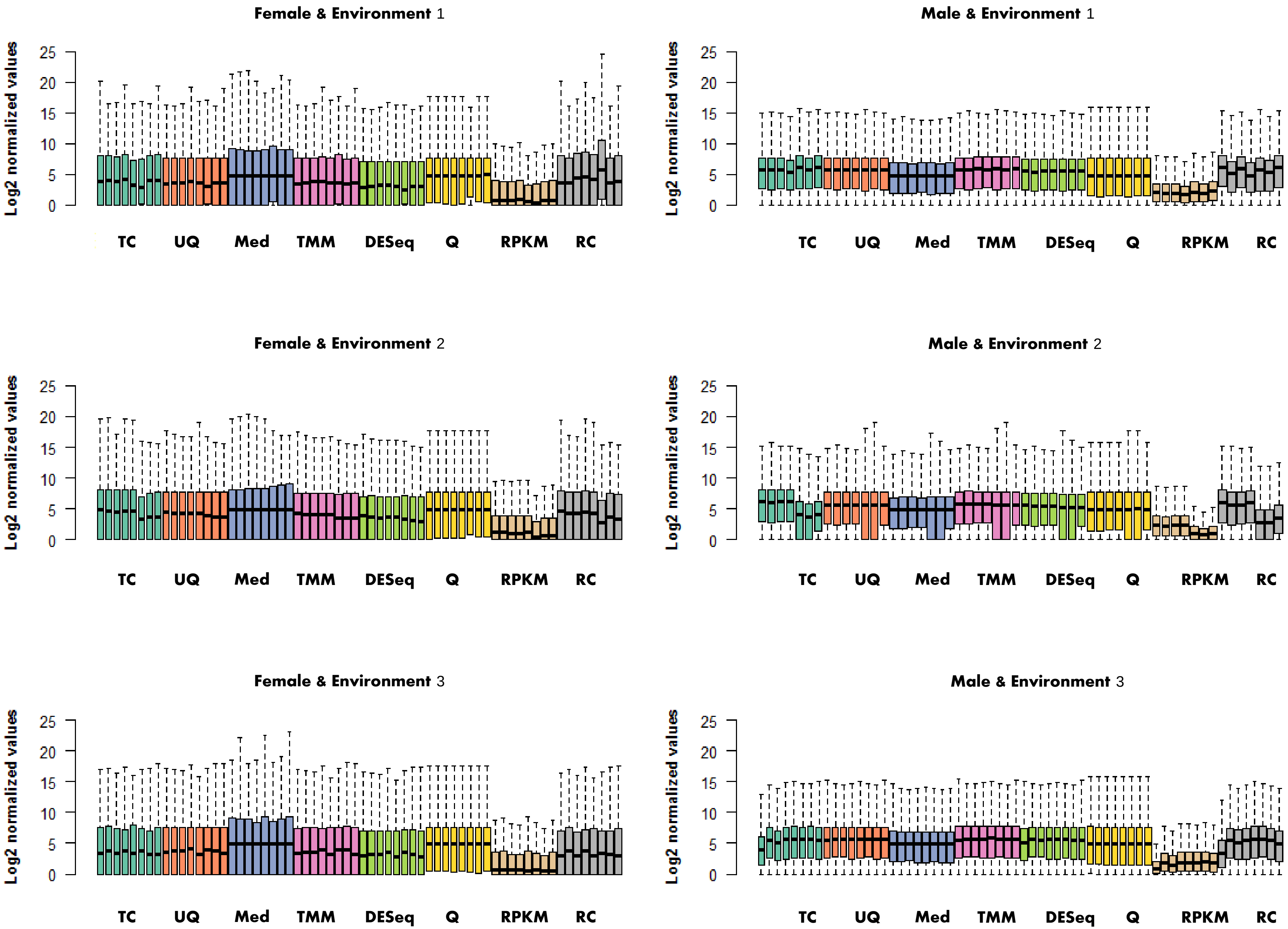

Female & Environment 1
Male & Environment 1
Female & Environment 2
Male & Environment 2
Female & Environment 3
Male & Environment 3
Log2 normalized values
0
5
10
15
20
25
TC
UQ
Med
TMM
DESeq
Q
RPKM
RC

Line: *RAL-850*

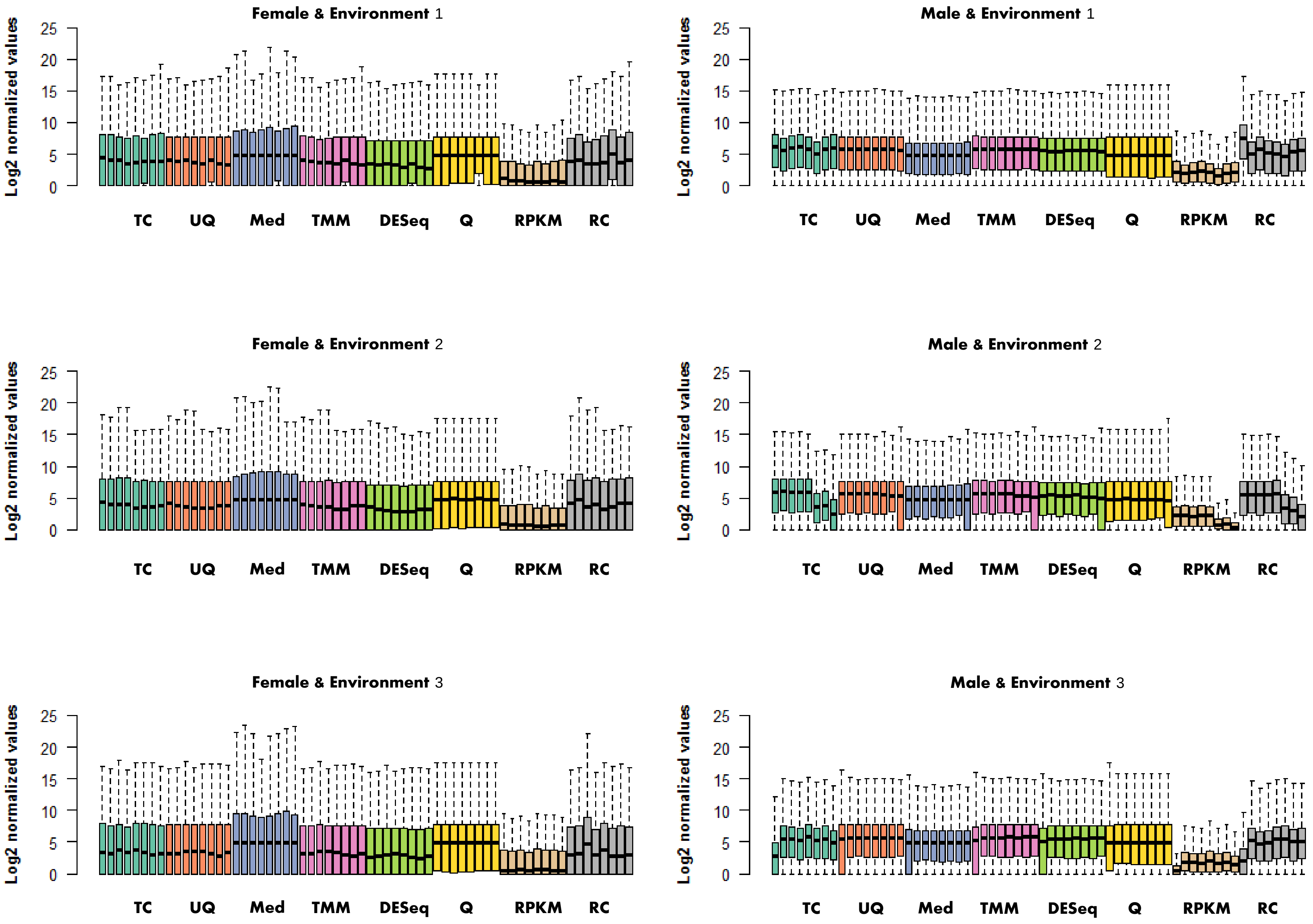
Female & Environment 1
Male & Environment 1
Female & Environment 2
Male & Environment 2
Female & Environment 3
Male & Environment 3
Log2 normalized values
0
5
10
15
20
25
TC
UQ
Med
TMM
DESeq
Q
RPKM
RC

Line: *RAL-900*

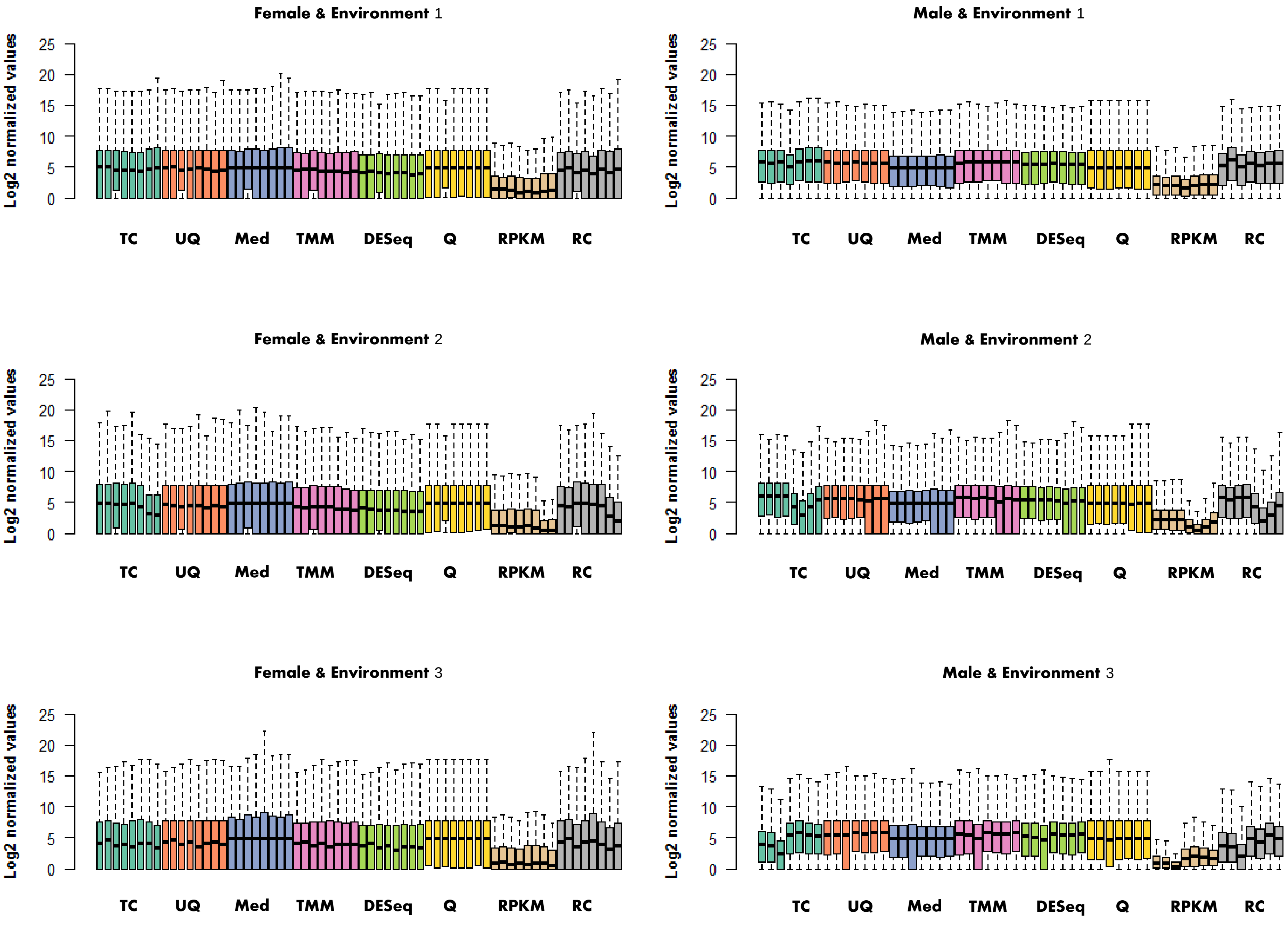
Female & Environment 1
Male & Environment 1
Female & Environment 2
Male & Environment 2
Female & Environment 3
Male & Environment 3
Log2 normalized values
0
5
10
15
20
25
TC
UQ
Med
TMM
DESeq
Q
RPKM
RC
