# Supplementary material for: Comparison of normalization and differential expression analyses using RNA-Seq data from 726 individual Drosophila melanogaster
Source: BMC Genomics. 2016 Jan 5;17:28. doi: 10.1186/s12864-015-2353-z (PMC4702322; doi:10.1186/s12864-015-2353-z)

CV for RAL93

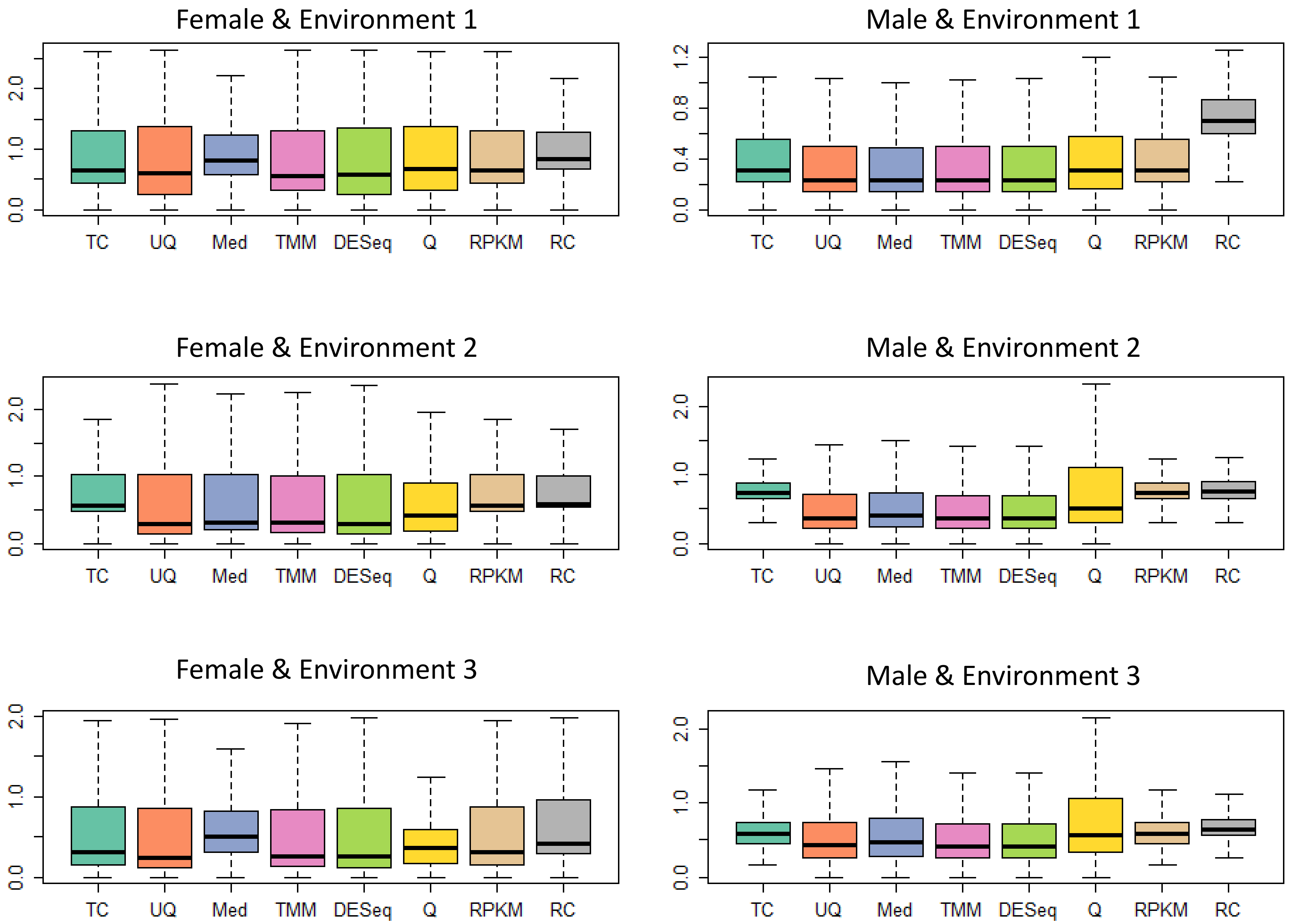

Female & Environment 1
Male & Environment 1
Female & Environment 2
Male & Environment 2
Female & Environment 3
Male & Environment 3
TC
UQ
Med
TMM
DESeq
Q
RPKM
RC
0.0
1.0
2.0
0.4
0.8
1.2

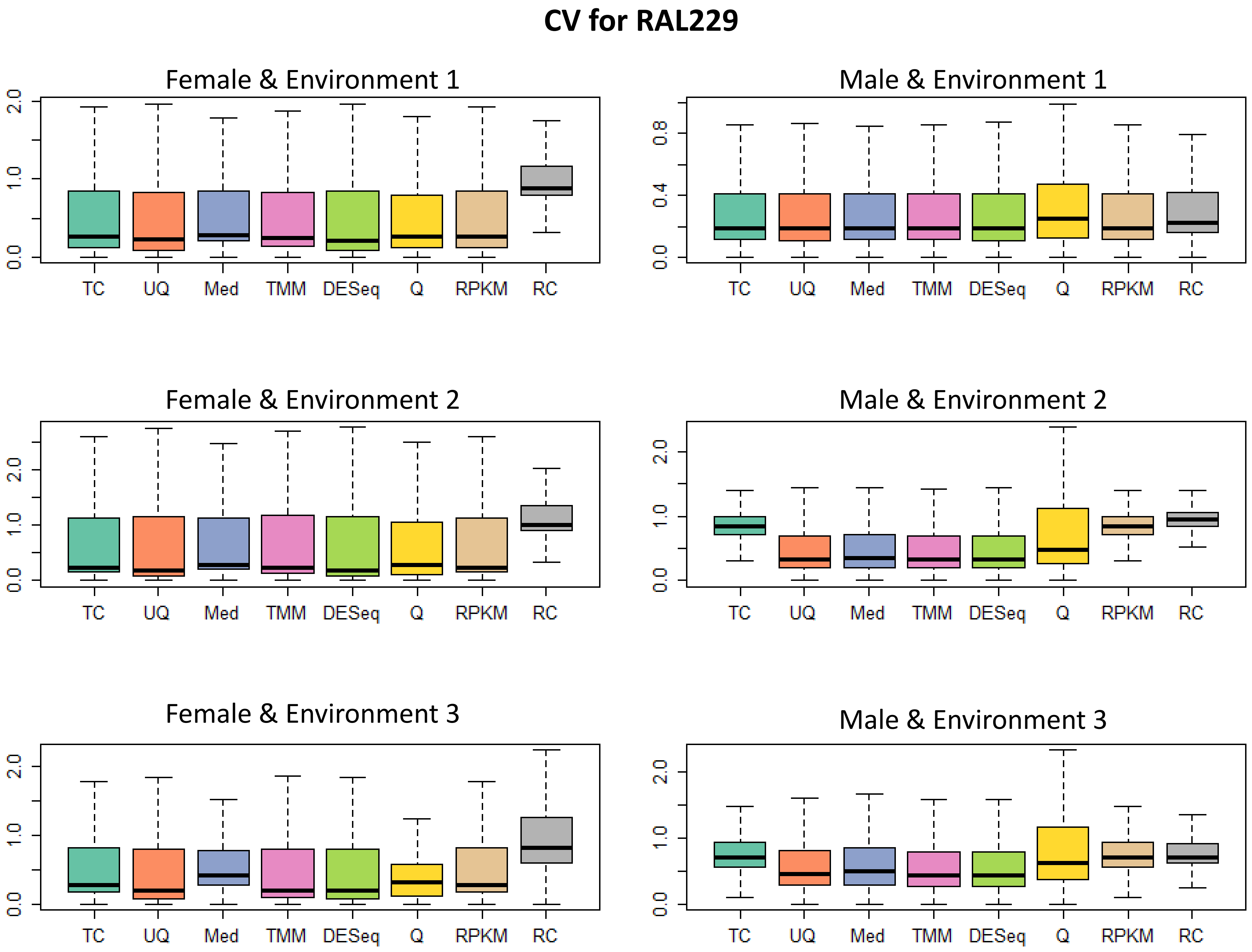
CV for RAL229
Female & Environment 1
Male & Environment 1
Female & Environment 2
Male & Environment 2
Female & Environment 3
Male & Environment 3
0.0
1.0
2.0
0.0
0.4
0.8
TC
UQ
Med
TMM
DESeq
Q
RPKM
RC

CV for RAL320

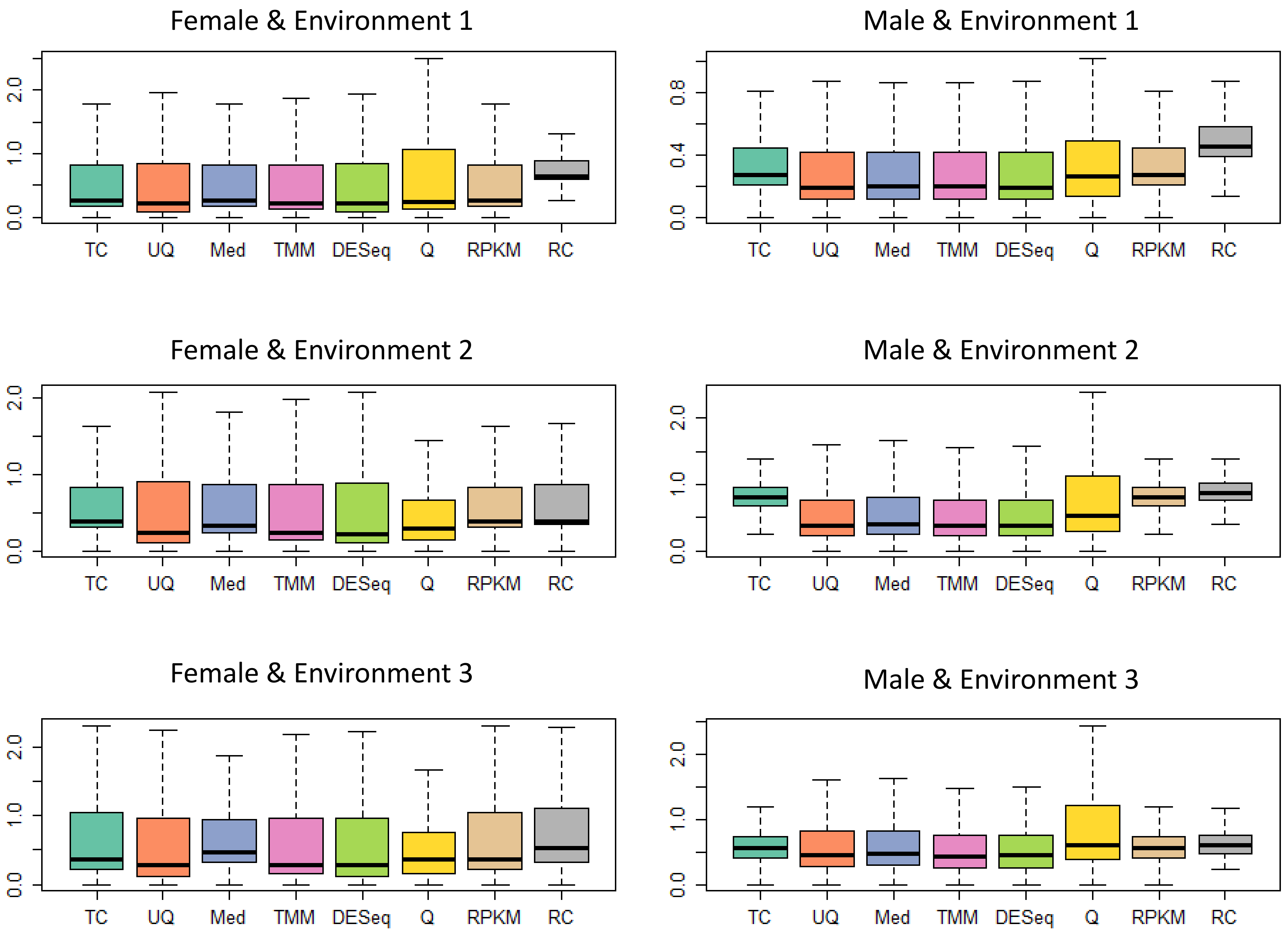

CV for RAL352

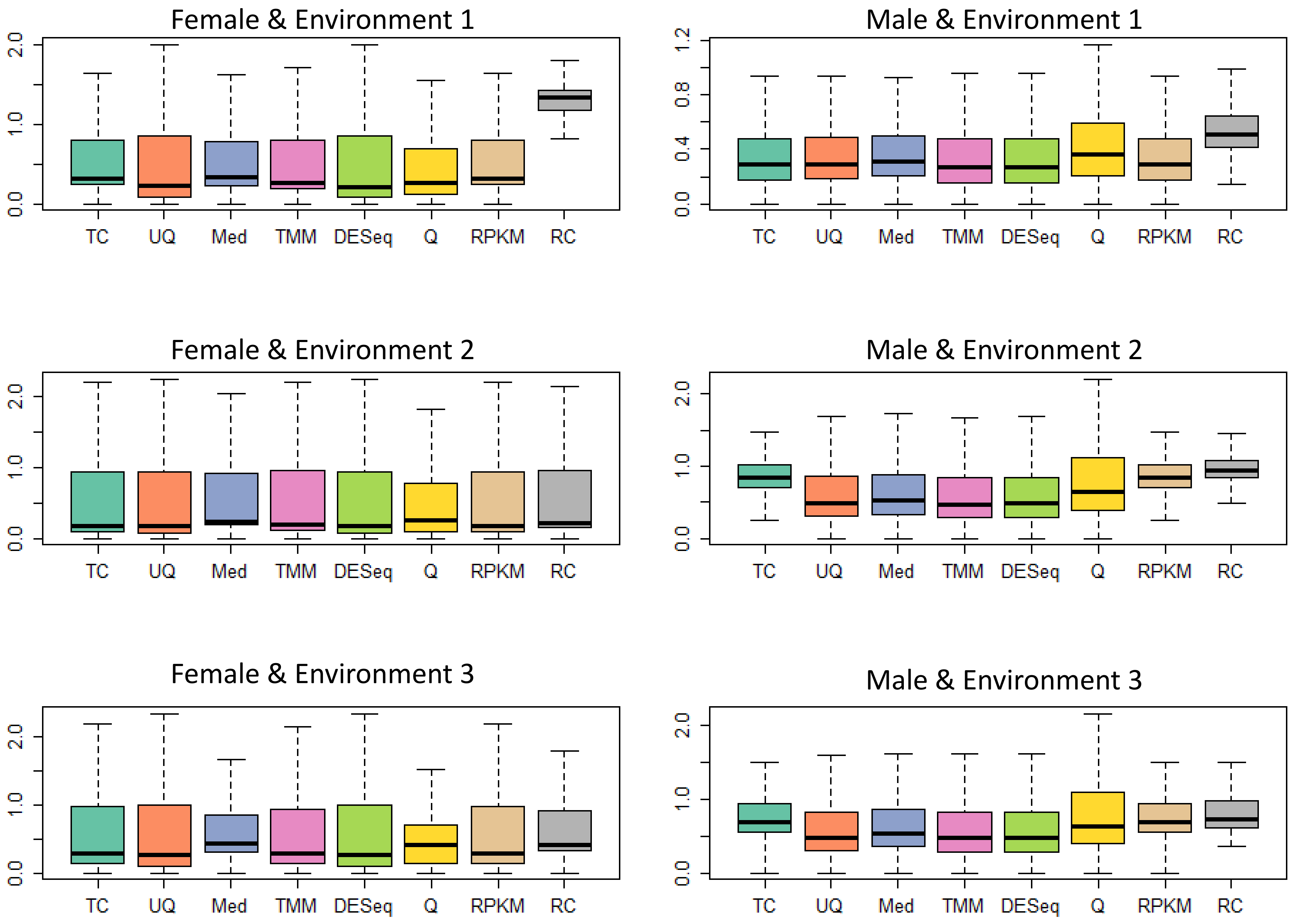

CV for RAL370

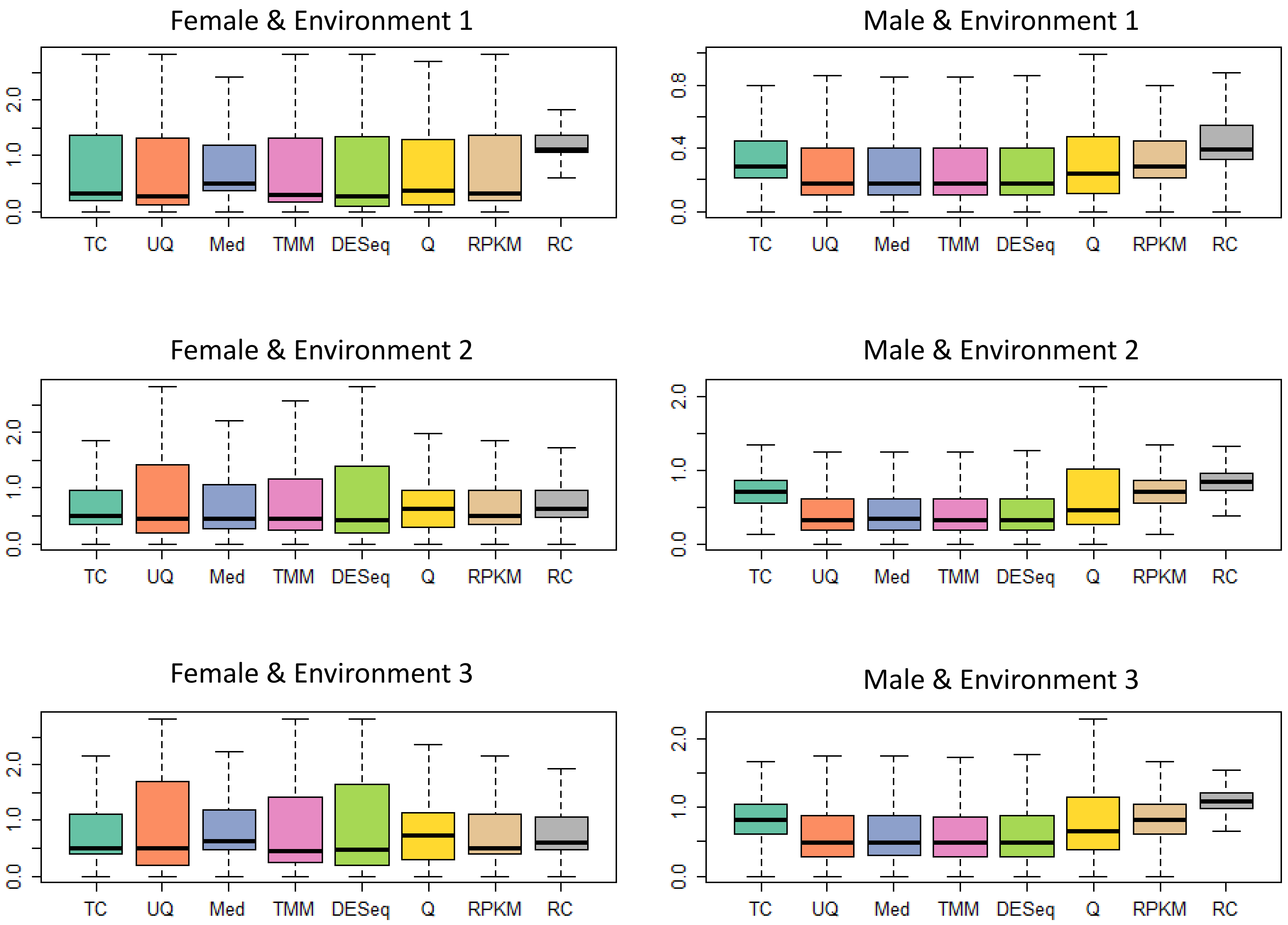

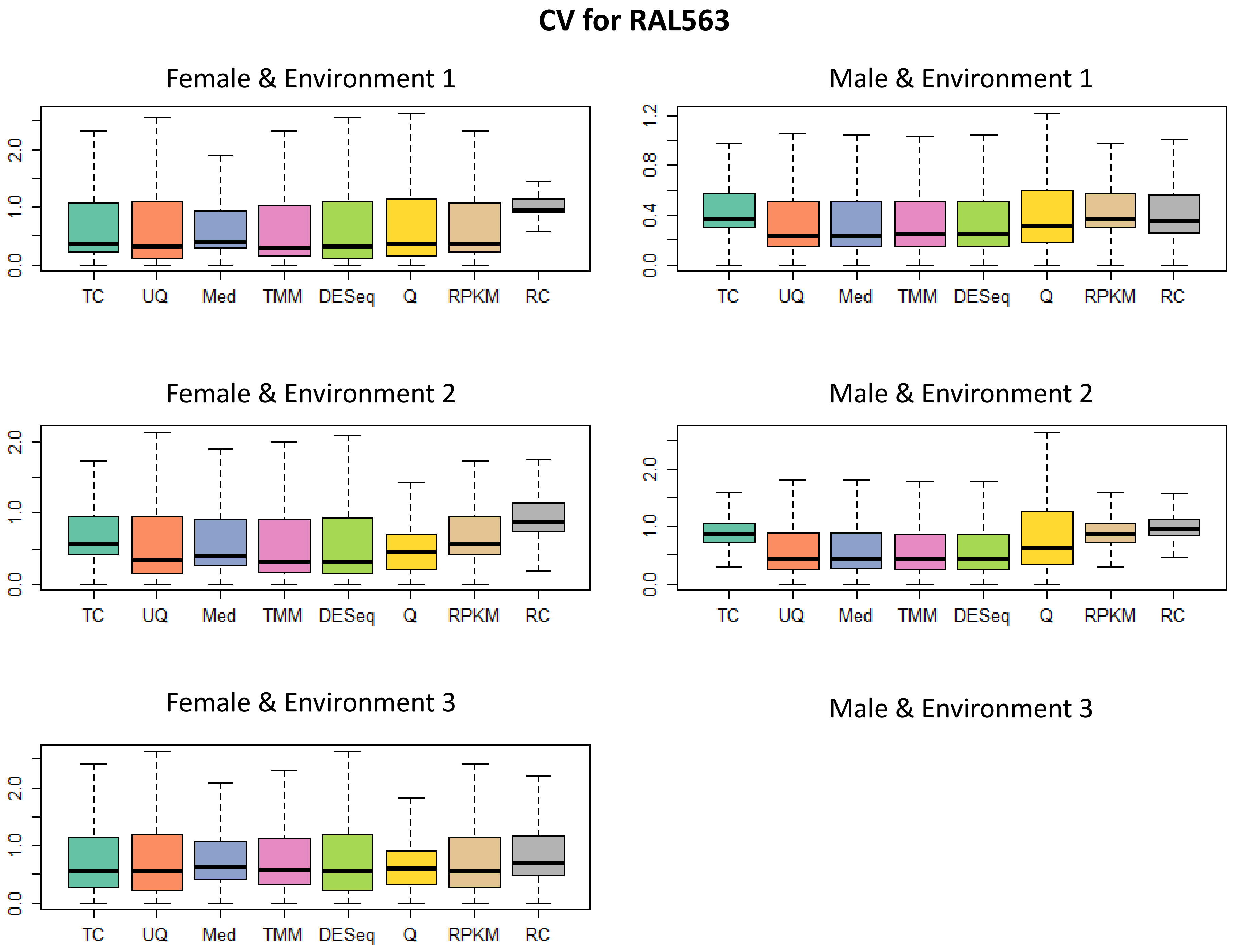
CV for RAL563
Female & Environment 1
Male & Environment 1
Female & Environment 2
Male & Environment 2
Female & Environment 3
Male & Environment 3
0.0
1.0
2.0
0.4
0.8
1.2
TC
UQ
Med
TMM
DESeq
Q
RPKM
RC

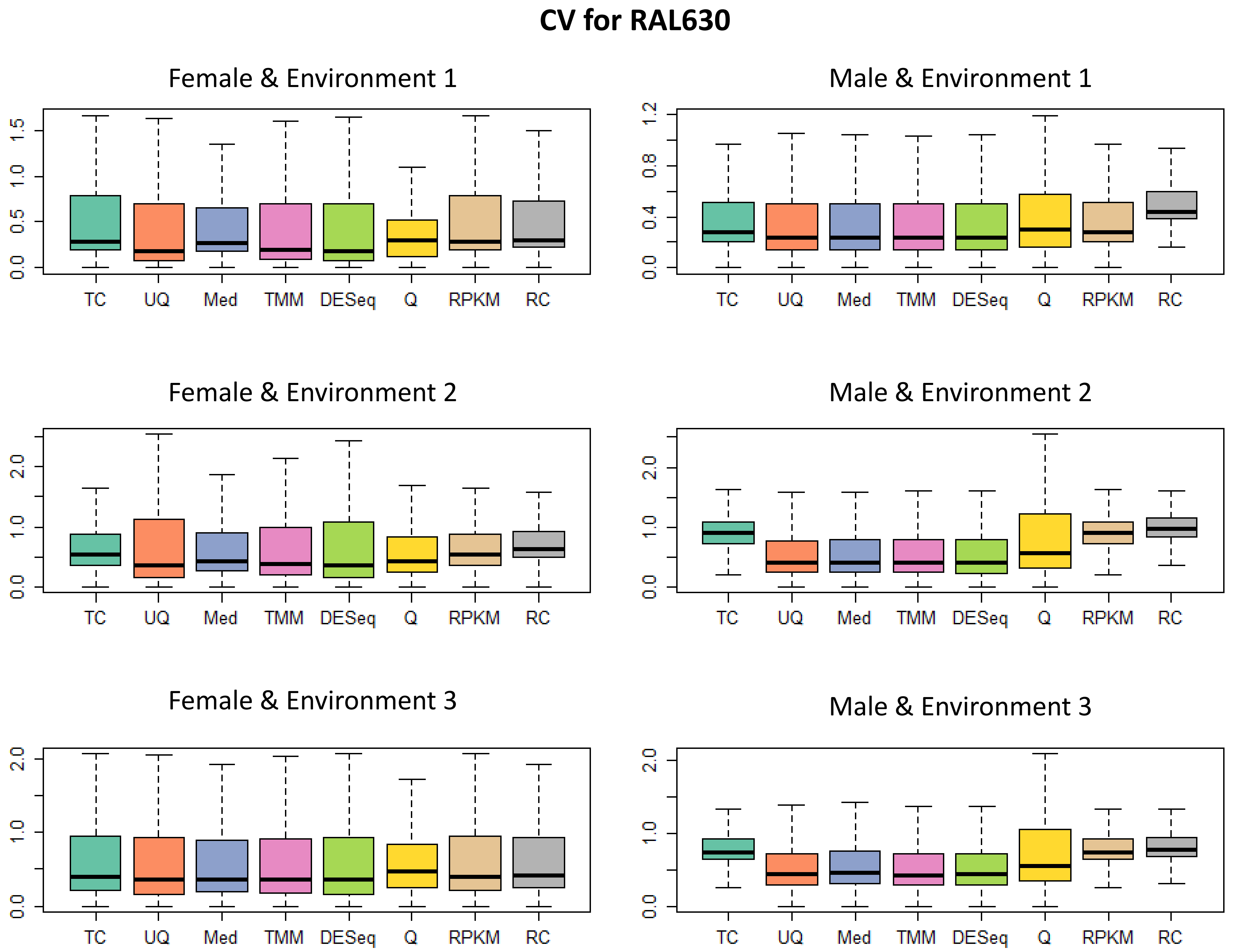
CV for RAL630
Female & Environment 1
Male & Environment 1
Female & Environment 2
Male & Environment 2
Female & Environment 3
Male & Environment 3
TC
UQ
Med
TMM
DESeq
Q
RPKM
RC
0.0
0.5
1.0
1.5
0.4
0.8
1.2
2.0

CV for RAL703

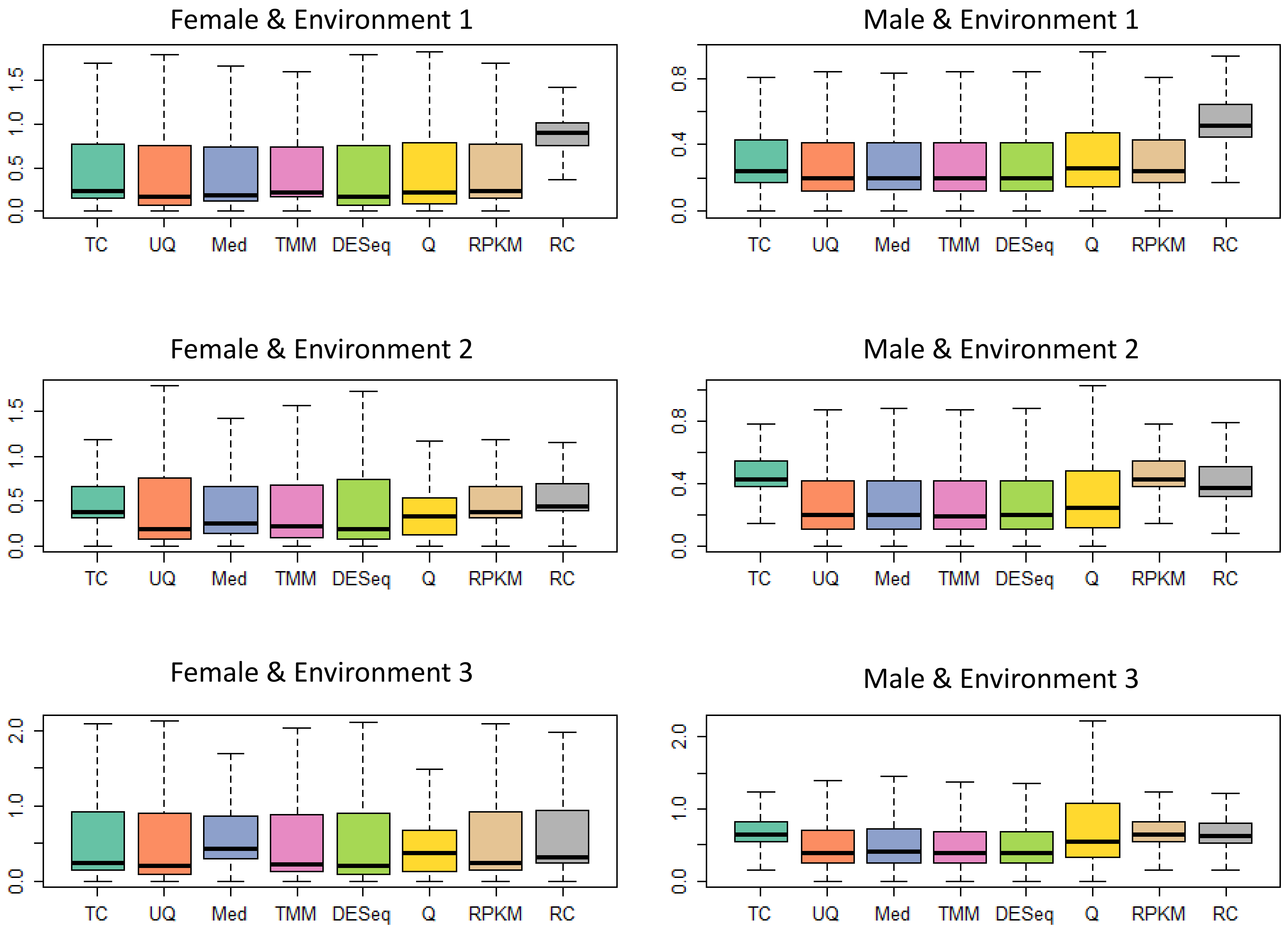

CV for RAL761

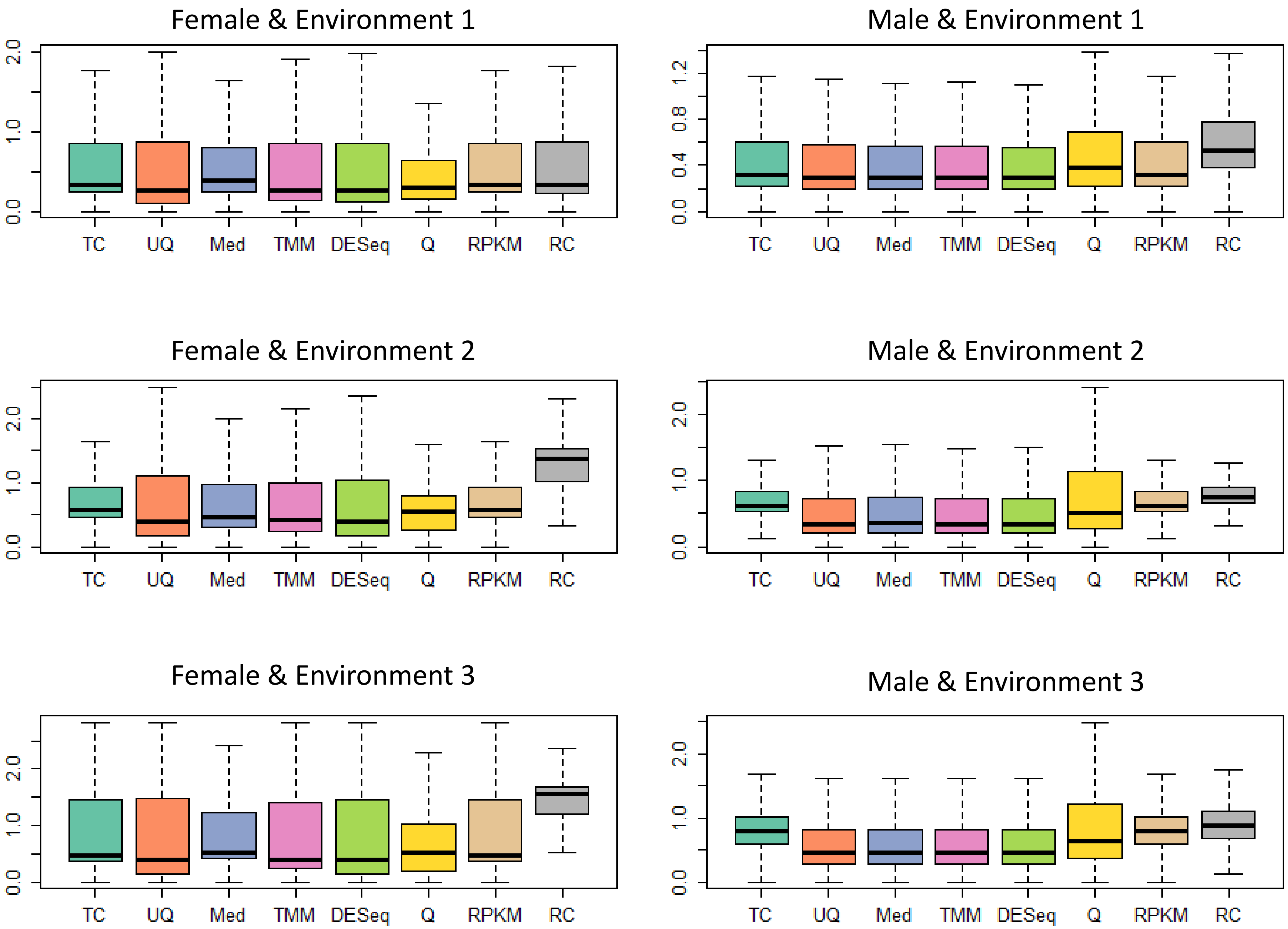

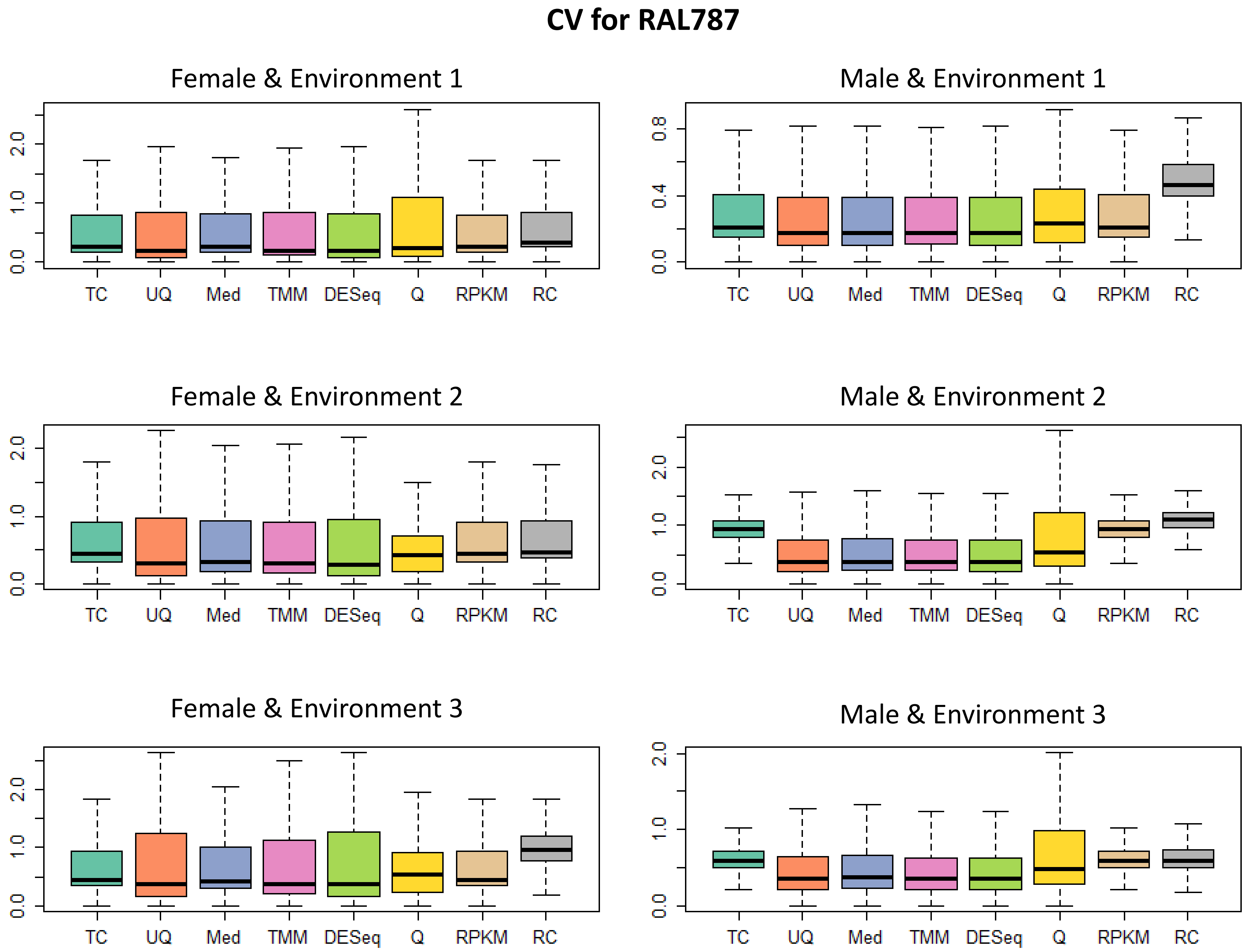
CV for RAL787
Female & Environment 1
Male & Environment 1
Female & Environment 2
Male & Environment 2
Female & Environment 3
Male & Environment 3
TC
UQ
Med
TMM
DESeq
Q
RPKM
RC
0.0
1.0
2.0
0.4
0.8

CV for RAL790

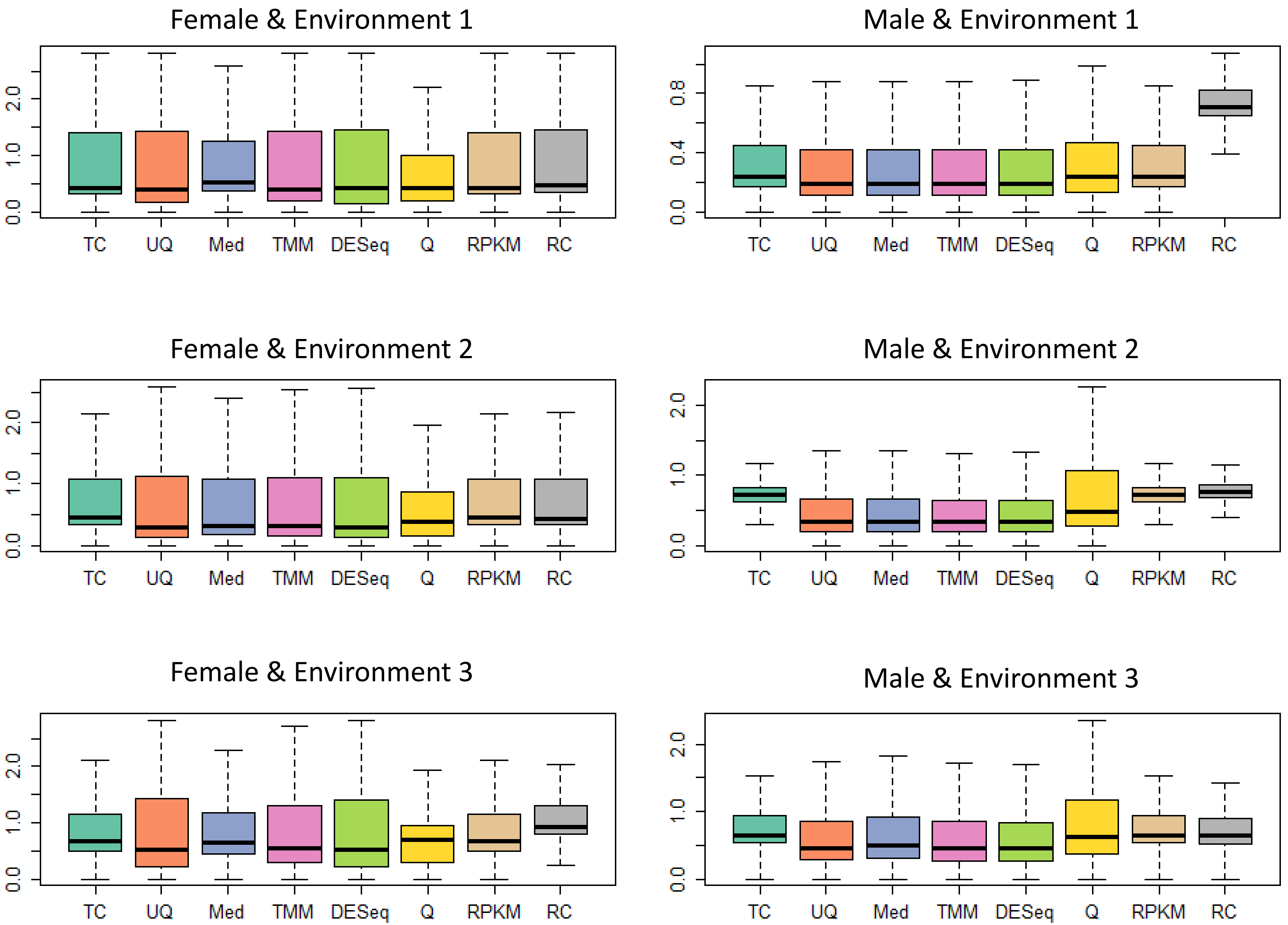

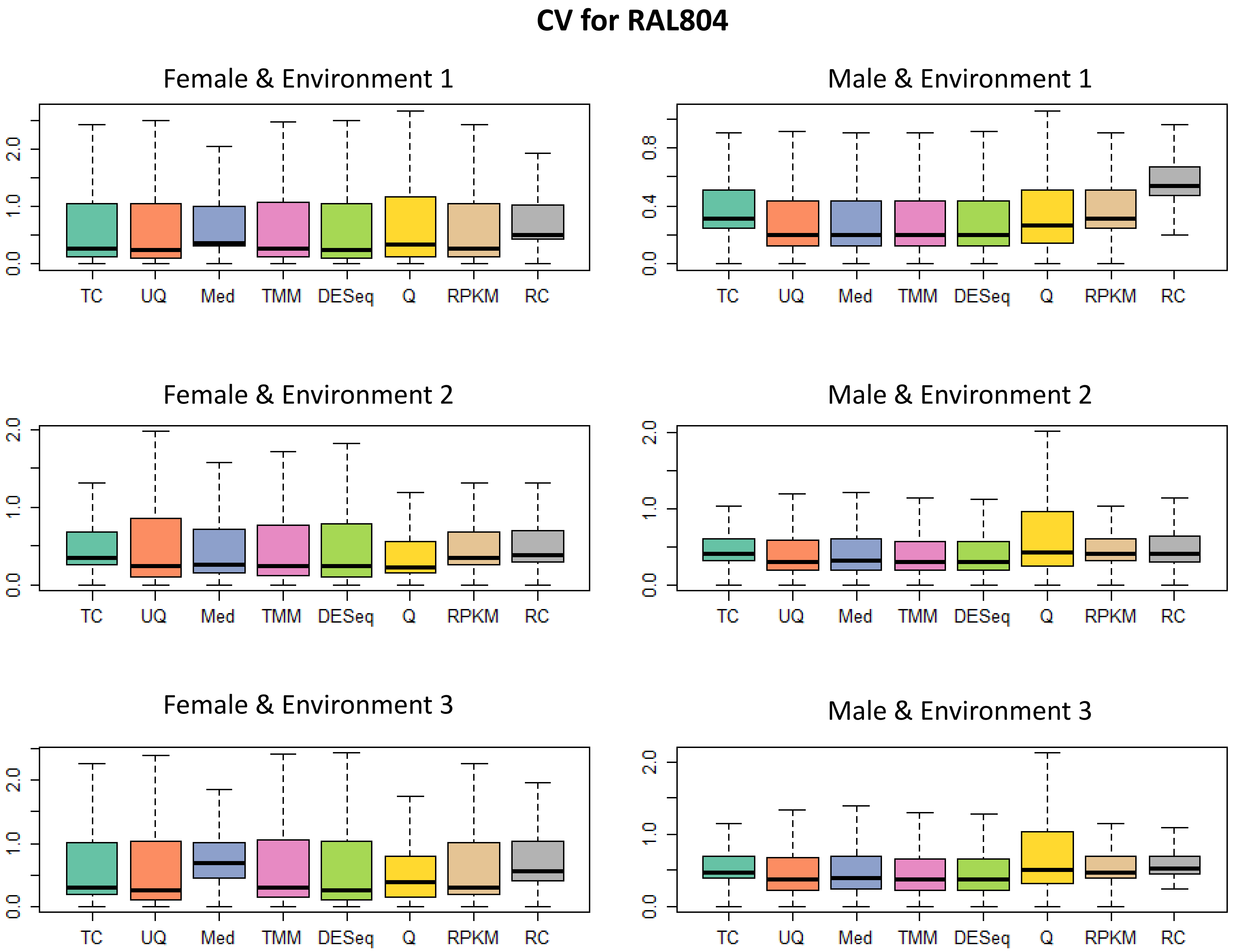

CV for RAL804
Female & Environment 1
Male & Environment 1
Female & Environment 2
Male & Environment 2
Female & Environment 3
Male & Environment 3
0.0
1.0
2.0
0.4
0.8
TC
UQ
Med
TMM
DESeq
Q
RPKM
RC

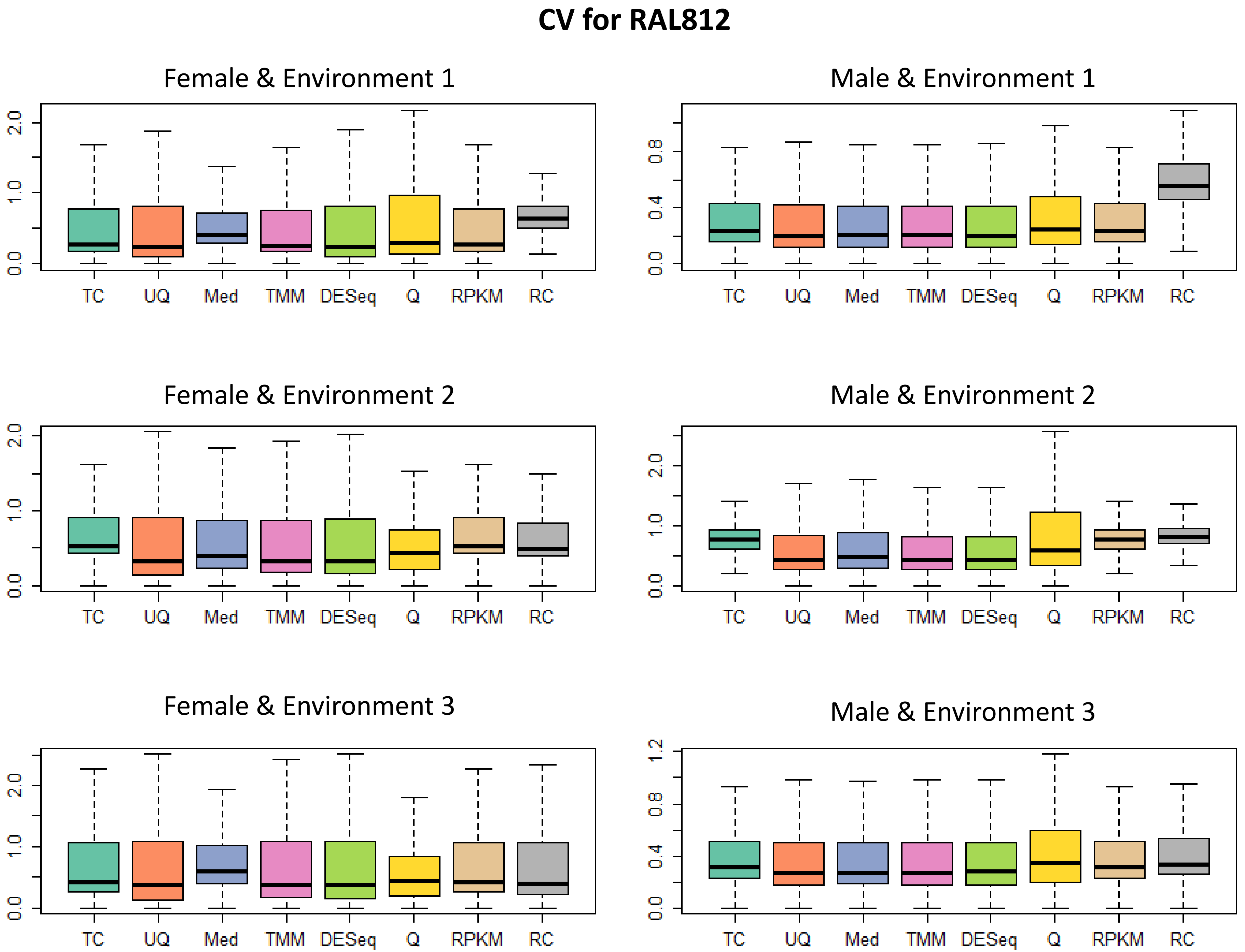
CV for RAL812
Female & Environment 1
Male & Environment 1
Female & Environment 2
Male & Environment 2
Female & Environment 3
Male & Environment 3
0.0
1.0
2.0
0.4
0.8
1.2
TC
UQ
Med
TMM
DESeq
Q
RPKM
RC

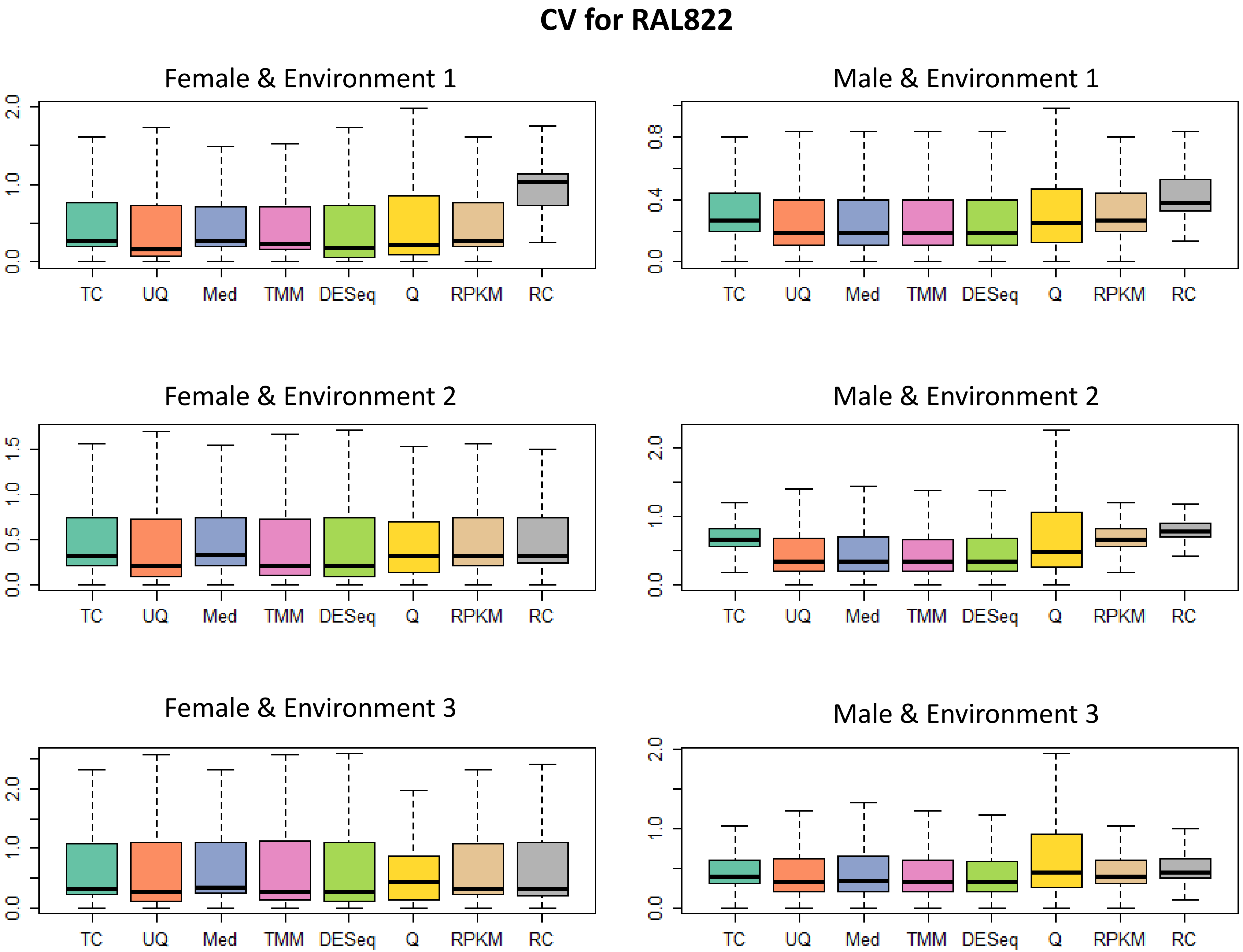

CV for RAL822
Female & Environment 1
Male & Environment 1
Female & Environment 2
Male & Environment 2
Female & Environment 3
Male & Environment 3
TC
UQ
Med
TMM
DESeq
Q
RPKM
RC
0.0
1.0
2.0
0.4
0.8
0.5
1.5

CV for RAL850

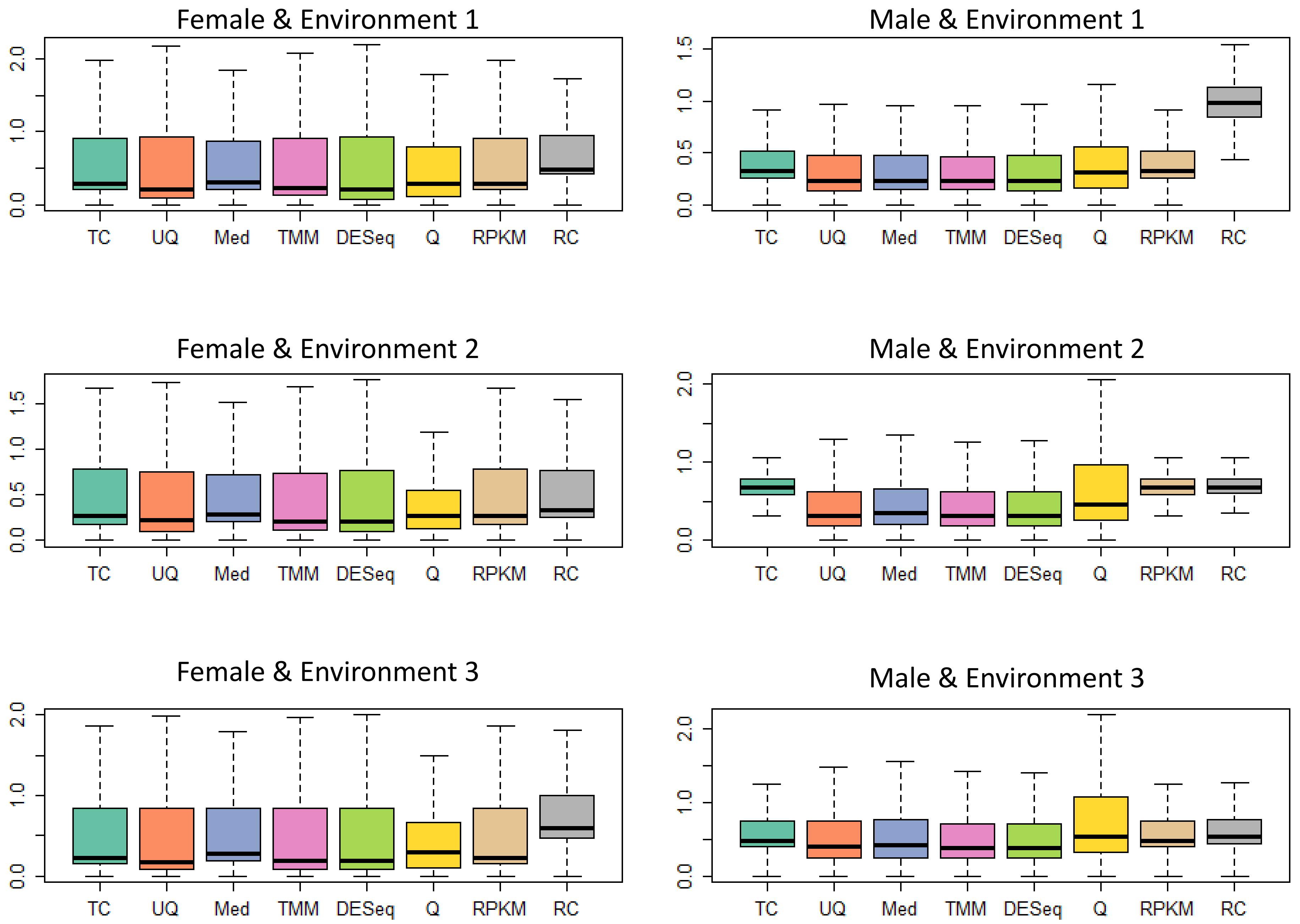

CV for RAL900

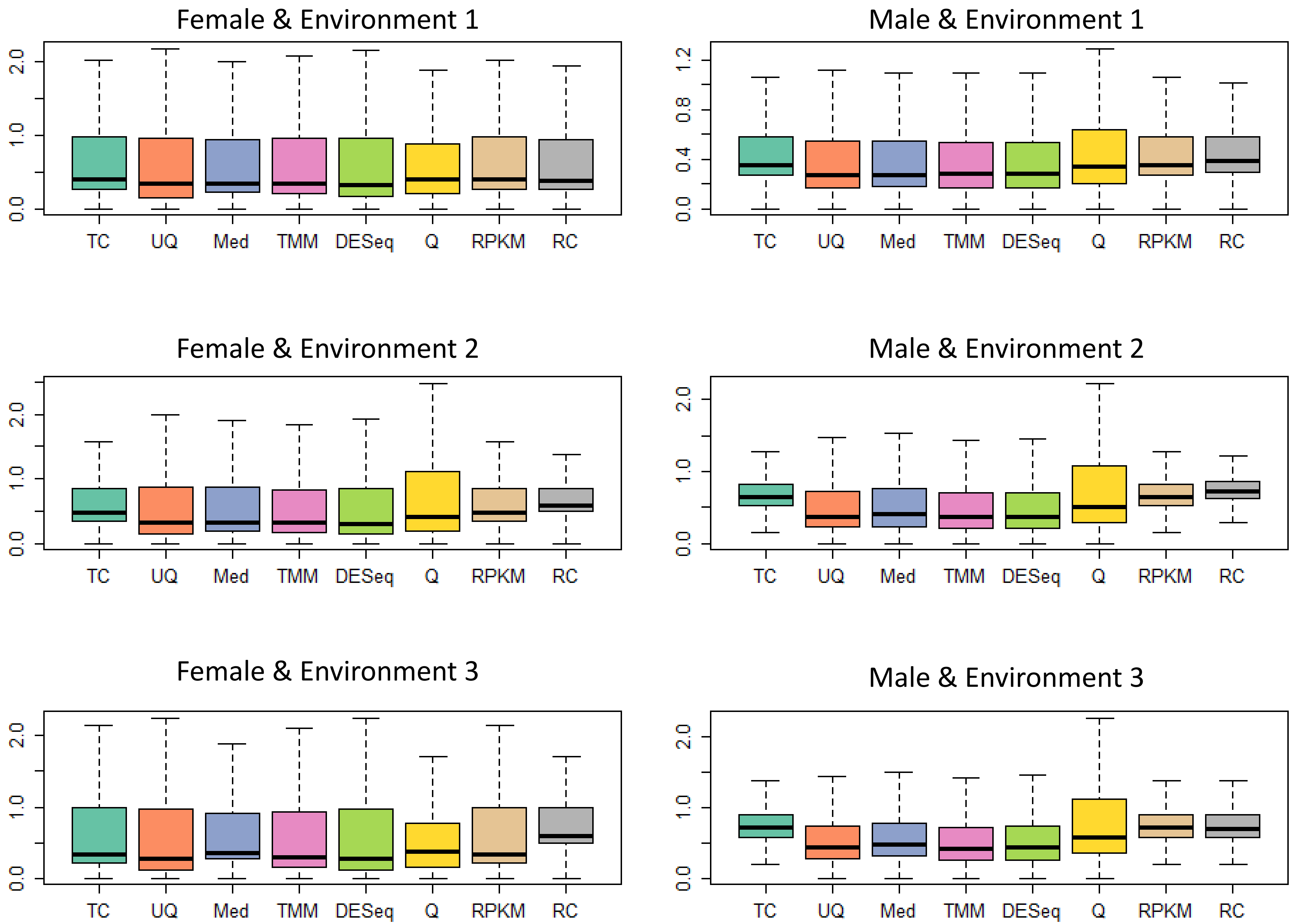

Supplement: Additional file 3: — Comparison of normalization methods across conditions. Boxplots show the differences in the coefficient of variation across flies in each genotype/sex/environment condition. (PDF 245 kb) [file 12864_2015_2353_MOESM3_ESM.pdf]
