# Supplementary material for: Comparison of normalization and differential expression analyses using RNA-Seq data from 726 individual Drosophila melanogaster
Source: BMC Genomics. 2016 Jan 5;17:28. doi: 10.1186/s12864-015-2353-z (PMC4702322; doi:10.1186/s12864-015-2353-z)
Supplement: Additional file 4: — Supplemental Methods. (PDF 133 kb) [file 12864_2015_2353_MOESM4_ESM.pdf]

Supplemental Methods

General molecular practices

Strand-specific libraries were prepared by modification of an existing protocol [1]. We completed all steps in 96-well plates. We incubated all enzymatic reactions in a Tetrad PTC-225 Thermal Cycler (MJ Research, Waltham, MA). To clean up enzymatic reactions and size-select nucleic acid species at any of several steps detailed below, we used 0.1% w/v carboxyl-modified Sera-Mag Magnetic Speed-beads (*MagNA* beads, Thermo Fisher Scientific, Waltham, MA) in XP buffer [20% PEG 8000, 2.5 M NaCl, (Sigma Aldrich, St. Louis, MO)] following the protocol in Rohland and Reich [2], except that we incubated samples with beads for 10 min and used 80% ethanol for washing. The volume of *MagNa* beads in XP buffer was 1.6X XP buffer for no size-selection (except where noted) and 1X XP buffer for selecting fragments > 200bp. Unless noted, we left the beads in the sample after each step. We used 96-well PCR plates (USA Scientific, Ocala, Fl) paired with Alpaqua 96R Ring Magnet Plates (Alpaqua, Beverly, MA) to separate RNA-bound magnetic beads from supernatant. We measured RNA and DNA quantity with Quant-iT™ RiboGreen and PicoGreen, respectively, (Invitrogen, Carlsbad, CA) in black FLUOTRAC 600 384-well plates, PS, (Greiner Bio-One Inc, Longwood, Fl) using a Gemini EM Fluorescence Microplate Reader (Molecular Devices, Sunnyvale, CA) according to manufacturer instructions. We used Agilent Bioanalyzer RNA chips and High Sensitivity DNA chips on the 2100 Bioanalyzer system (Agilent, Santa Clara, CA) according to manufacturer instructions and visually inspected electropherograms for RNA degradation, desired library size, and unwanted adapter dimers.

mRNA isolation

Single flies frozen on dry ice were placed into each well of Axygen 96 Deep Well Plates (Corning, Corning, NY) pre-loaded with 200µL of 1 mm glass beads (Biospec Products, Bartlesville, OK). We randomized fly placement in the wells of each plate within each environment. We isolated total RNA using the RNeasy 96 Plate Kit (Qiagen, Valencia, CA) according to the manufacturer instructions using either vacuum or spin technology with the following modifications. We added 200µl RTL buffer to each well, sealed the plates with Axygen Sealing Mats (Corning, Corning, NY), and homogenized flies at room temperature for 30 sec using a fixed-speed (36 oscillations/sec) Mini Bead Beater (Biospec Products, Bartlesville, OK). We added 200µl of 70% ethanol to the crude homogenate, mixed by pipetting several times, and transferred the supernatant to RNeasy plates. We recorded yield by fluorometry and pass/fail quality on the Bioanalyzer. We used Dynabeads Oligo $(dT)_{25}$ (Life Technologies, Carlsbad, CA) to purify mRNA according to the manufacturer instructions, except that we used 50-200ng of total RNA and adjusted the volume to 50µl with dH_2O, added 10µl of washed Dynabeads in a 50µl binding buffer slurry, and heated the samples at 65°C for 5 min before chilling on ice.

cDNA library preparation

We fragmented mRNA bound to Dynabeads at 94°C for 8 min in 16µl of 1.25X first strand MMuLV RT buffer (New England Biolabs, Ipswich, MA), with 100ng random primers (Invitrogen, Carlsbad, CA), and 10pg ERCC spike-in controls [3] from pools 78A and 78B [4] . We chilled the samples immediately on ice for 2 min and eluted the mRNA from the beads. We transferred 15µl of eluate to a fresh 96-well PCR plate. We added 5µl of the first strand synthesis mixture [0.3mM dNTPs, 5mM DTT, and 10U M-MuLV RT (New England Biolabs, Ipswich, MA), and 0.5U SuperRase-In (Life Technologies, Carlsbad, CA)] to the fragmented mRNA in fragmentation buffer and performed a reverse transcription reaction. We bound the mRNA/cDNA hybrid with 32µl of *MagNA* bead XP buffer slurry, washed the samples, and then eluted them in 16µl of dH_2O. We then performed a second strand synthesis with dUTP by adding 5µl of 1X NEB buffer2 (New England Biolabs, Ipswich, MA), with 1mM each of dATP, dCTP, dGTP and 2mM dUTP (Thermo Fisher Scientific, Waltham, MA), 10U DNA PolI, 2.5U RNAseH, and 2.5mM DTT (New England Biolabs, Ipswich, MA) and incubated at 16°C for 5 hours. We bound the samples to *MagNA* beads, washed, and eluted as above. We repaired ends by adding 4µl of NEBNext End Repair Module (New England Biolabs, Ipswich, MA) to the eluate following the manufacturer's instructions. We then bound samples to *MagNA* beads, washed, and eluted as above. We performed dA-tailing by adding 4µl of 1X Blue Buffer, with 1U Klenow 3'-5' exo- (Enzymatics, Beverly, MA) and 1mM dATP, to the eluate and incubated at 37°C for 30 min. We then bound samples to *MagNA* beads and washed as above, eluted the samples with 21µl dH_2O, and transferred 10µl of cDNA to each of two fresh plates. One plate was used for further processing; the other was frozen as a back-up. We ligated RNA Adapter Indexes AR001–AR016, AR018–AR023, AR025, and AR027 (Illumina, San Diego, CA) to dsDNA by adding 1µl of an adapter to each 10µl sample and 13µl of 1X Rapid Ligation Buffer with 30U T4 DNA Ligase (Enzymatics, Beverly, MA), and incubated at 25°C for 10 min. We stopped reactions with a final concentration of 0.01M EDTA. We added 25 µl *MagNA* beads in XP buffer (final PEG = 13.6%) to bind cDNA to the beads, washed, and eluted in 30µl of dH_2O as above. We added 1X XP buffer (30µl) to remove adapter dimers and select libraries for the desired size range (300-350 bp), washed samples and eluted them with 24µl dH_2O, and transferred 23µl of cDNA to fresh plate. 11.5µl of the eluate was transferred to another plate and used in the next step; the remaining samples were frozen as a back-up. We mixed dsDNA product with 2.5U (0.5µl) of Uracil DNA Glycosylase (New England Biolabs, Ipswich, MA) and 3µl of PCR Primer Cocktail (Illumina, San Diego, CA) and incubated at 37°C for 30 min to digest the second strand DNA. We added 15µl 2X KAPA HiFi HotStart ReadyMix (Kapa Biosystems, Woburn, MA) directly to the UDG-digested DNA mixture and performed a PCR amplification with the following programmed cycle: 95°C for 2 min, followed by 12 cycles of 98°C for 20 sec, 65°C for 30 sec, and 72°C for 30 sec; then 72°C for 5 min. We purified the product by adding 30µl *MagNa* beads in 1X XP buffer, eluted in 30µl dH_2O, and transferred 29µl of cDNA library to a fresh plate as described above. To assay plate-level failure, we examined electropherograms from 11 samples randomly chosen from each 96-well plate. We verified that dominant signals were in the range of the 300-350 bp library target size and not in the 100-150 bp range (primer dimer products).

Sequencing and alignment

Each 96-well plate was composed of 4 sets of samples with 24 unique indices. We added equal amounts of each library to create 4 pools of 24 samples for multiplex sequencing. All multiplexed libraries were

again quantified and checked for quality as above. Excess library pools were frozen for re-sequencing if required. We performed single-end 76 bp sequencing reactions on a HiSeq2000 (Illumina, San Diego, CA) according to the manufacturer. We mapped reads that passed Chastity (score > 0.6) base-calling filtering (Illumina CASAVA pipeline 1.8.2).

RNA sequences were aligned to the BDGP5/dm3 *Drosophila melanogaster* assembly using TopHat2 software (v2.0.8) [5]. We used a GTF format reference annotation of the *Drosophila melanogaster* genome (release 5, GenBank Assembly ID GCA_000001215.1), excluding "crhUextra" and "chrU" with 96 ERCC sequences supplied following the "-G" option. This software allows quality-based mapping with parameters: "-g 1 --library-type fr-firststrand" (-g 1: specifically keep only uniquely mapped reads; --library-type fr-firststrand: strand-specific RNA-seq) [5]. Only uniquely mapped reads with less than two mismatches were used. We decided to use samples having a minimum of 2.5 million uniquely mapped reads or greater (ModENCODE Consortium, personal communication) [6,7]. Raw reads mapped to each gene were calculated by HT-Seq (v0.5.3p9) (HTSeq) with parameters: "-m union -s reverse" (-m union: reads with only one overlapping gene will be assigned ambiguously to that gene; -s reverse: stranded RNA-seq). The output numbers of mapped reads were used for further normalization.

REFERENCES

1. Wang L, Si Y, Dedow LK, Shao Y, Liu P, Brutnell TP. A low-cost library construction protocol and data analysis pipeline for Illumina-based strand-specific multiplex RNA-seq. *PLoS One.* 2011;6:e26426.

2. Rohland N, Reich D. Cost-effective, high-throughput DNA sequencing libraries for multiplexed target capture. *Genome Research*. 2012;22:939-946.

3. Jiang L, Schlesinger F, Davis CA, Zhang Y, Li R, Salit M, Gingeras TR, Oliver B. Synthetic spike-in standards for RNA-seq experiments. *Genome Research*. 2011;21:1543-1551.

4. Zook JM, Samarov D, McDaniel J, Sen SK, Salit M. Synthetic spike-in standards improve run-specific systematic error analysis for DNA and RNA sequencing. *PLoS ONE*. 2012; doi: 10.1371/journal.pone.0041356.

5. Kim D, Pertea G, Trapnell C, Pimentel H, Kelley R, Salzberg SL. TopHat2: accurate alignment of transcriptomes in the presence of insertions, deletions and gene fusions. *Genome Biol.* 2013;14:R36.

6. Malone JH, Oliver B. Microarrays, deep sequencing and the true measure of the transcriptome. *BMC Biol.* 2011; 9:34.

7. Sturgill D, Malone JH, Sun X, Smith HE, Rabinow L, Samson ML, Oliver B. Design of RNA splicing analysis null models for post hoc filtering of Drosophila head RNA-Seq data with the splicing analysis kit (Spanki). *BMC Bioinformatics*. 2013; 14:320.
