# Supplementary material for: Comparison of normalization and differential expression analyses using RNA-Seq data from 726 individual Drosophila melanogaster
Source: BMC Genomics. 2016 Jan 5;17:28. doi: 10.1186/s12864-015-2353-z (PMC4702322; doi:10.1186/s12864-015-2353-z)
Supplement: Additional file 5: — R code pipeline for analysis. (PDF 245 kb) [file 12864_2015_2353_MOESM5_ESM.pdf]

RNA_Seq Data Analysis Pipeline

This pipeline provides the R commands used to run the RNA-Seq data analysis presented in "Comparison of normalization and differential expression analyses using RNA-Seq data from 726 individual *Drosophila melanogaster*" by Lin et al. Very small example files are presented and analyzed in this pipeline. Here we follow the convention of the R code terminal window: coding commands are in red, while the R terminal's response to those commands are given in blue. Users interested in this pipeline will need to have some familiarity with R code, DESeq, edgeR, limma, and RNA-Seq read count data before proceeding. This pipeline is designed to be executed in the order listed below.

Outline:

I. Some useful links for basic R commands

II. Package installation
(a) Installation with the use command
(b) Installation of R packages from the Bioconductor website

III. Calling libraries and packages in R

IV. Reading data into R
(a) A note about the directory path
(b) Two methods for reading in data

V. Saving your results from R

VI. Identification of the low-expression threshold using genic and intergenic read count data
(a) Read in the genic read counts
(b) Read in the intergenic read counts
(c) Merge the genic and intergenic read counts together
(d) Read in the background information of biosample
(e) Identify the low-expression threshold (LET) using 95% upper-quartile of log2(normalized_intergenic_read_counts +1)

VII. Performing the RNA-Seq data analysis using the DESeq R package
(a) Read in the gene read counts and background conditions
(b) Identify the genes that fail to pass the low-expression threshold cutoff value
(c) Fit the GLM and test hypotheses

VIII. Implementation of RNA-Seq read counts normalization methods in R (if desired by the user)

IX. Estimate dispersion using the DESeq R package (if desired by the user)
(a) Read in the data in the format required by DESeq
(b) Estimate the dispersion for hypothesis testing
(c) Estimate dispersion per gene separately for each analysis factor or combination of factors (if desired by the user)

X. Performing the RNA-Seq data analysis using edgeR R package (if desired by the user)
(a) Read in data in the format required by edgeR
(b) Normalize data and estimate dispersion
(c) Fit the GLM and test hypotheses
(d) Save the results including *P*-values and FDR values

I. Some useful links for basic R commands

R reference card: https://cran.r-project.org/doc/contrib/Baggott-refcard-v2.pdf

Official R Website with Good Manuals: http://www.r-project.org/

https://www.calvin.edu/~scofield/courses/m143/materials/RcmdsFromClass.pdf

http://www.math.umt.edu/olear/stat458/Rseminar_2.pdf

II. Package installation

Libraries or packages that are not already installed in R can be installed in the following two ways:

(a) Installation with the use command

```
>install.packages("R_package_name")
```

(b) Installation of R packages from the Bioconductor website

```
>source("https://bioconductor.org/biocLite.R")
>biocLite("R_package_name")
```

III. Calling libraries and packages in R

When you want to use some R functions within a particular library, you will always need to call the library first. For example, you need to type in

```
>library(DESeq)
```

if you want to use the R functions within the DESeq R package. If you happen to know the name of the R function, but you don't know which library it belongs to, you can either search for it using your web browser or type help(R_function_name) at the R terminal prompt as shown in the example below.

```
>help(estimateDispersions)
```

IV. Reading data into R

(a) A note about the directory path

The directory path of input file in R should be written using the forward slash "/", which is different from the backward slash “\” you might normally encounter in Windows and copy from the file properties.

For example: if you have a file saved in following directory L:\Lab\example
Your input directory path in R should be "L:/Lab/example"

(b) Two methods for reading in data

(i) read.table(): usually works for text files.

Ex1: read in a text file "read_count.txt" with "," delimiter into R and assign it to the variable infile:

```
>infile<-read.table("L:/Lab/example/read_count.txt",header=TRUE,sep=",")
```

Ex2: read in a text file "read_count.txt" with tab delimiter into R and assign it to the variable infile:

```
>infile<-read.table("L:/Lab/example/read_count.txt",header=TRUE,sep="\t")
```

Ex3: read in a text file "read_count.txt" with space delimiter into R and assign it to the variable infile:

```
>infile<-read.table("L:/Lab/example/read_count.txt",header=TRUE,sep=" ")
```

Ex4: if the input file doesn't have a header, then you can use the command below:

```
>infile<-read.table("L:/Lab/example/read_count.txt",header=FALSE,sep=" ")
```

Note: you can type help("read.table") in R for more details.

(ii) read.csv(): works for comma-separated values (csv) files.

Ex1: read in a csv file “read_count.csv” with comma delimiter into R and assign it to the variable infile:

```
>infile<-read.csv("L:/Lab/example/read_count.csv", header = TRUE, sep = ",")
```

V. Saving your results from R

If you would like to save a data set from the R terminal into your local drive, you can use write.table() function.

Ex: you would like to save a data set called ResDE.txt into the directory: "L:\Lab\example"

```
>write.table(ResDE,"L:/Lab/example/ResDE.txt",quote=FALSE,col.names=TRUE,row.names=TRUE,sep="\t")
```

Note: If the data doesn't have the row/column names or you don't want to save the data with the row/column names, you can use the option: col.names=FALSE,row.names=FALSE. Note also that sep="\t" means that you will use a tab delimiter in your saved file. You may use other delimiters, such as sep="," for a comma delimiter.

VI. Identification of the low-expression threshold using genic and intergenic read count data

The sample code that follows uses two small data sets for genic and intergenic read counts: readCounts_genic.txt, and readCounts_intergenic.txt. A third file, biosample_summary.txt, is used to identify the genotype, sex, and environment that the data belong to. These files can be constructed very quickly using a text editor.

(a) Read in the genic read counts
readCounts_genic.txt is as follows:

variable_name,biosample1,biosample2,biosample3,biosample4,biosample5,biosample6,biosample7,bio
sample8,biosample9,biosample10,biosample11,biosample12,biosample13,biosample14,biosample15,bi
osample16
gene_1,0,0,0,0,0,0,1,2,0,0,0,0,0,2,0,3
gene_2,92,161,76,70,140,88,70,90,8,218,4,10,76,23,0,103
gene_3,5,1,0,0,4,0,0,7,0,2,6,21,5,17,2,8
gene_4, 5,36,4,1,15,10,7,19,3,4,1,0,4,13,4,0
gene_5,4664,8714,3564,3150,6205,3072,3334,4300,253,459,1211,1452,542,482,1568,244
gene_6,583,761,245,310,722,299,308,437,111,450,32,29,158,80,194,269

To read in the genic read counts, type

```
>genic<-read.table("L:/Lab/example/readCounts_genic.txt",header=TRUE,sep=",")
```

(b) Read in the intergenic read counts

readCounts_intergenic.txt is as follows:

variable_name,biosample1,biosample2,biosample3,biosample4,biosample5,biosample6,biosample7,bio
sample8,biosample9,biosample10,biosample11,biosample12,biosample13,biosample14,biosample15,bi
osample16
intergenic_1,1,7,3,0,1,2,1,2,2,8,4,2,5,1,2,1
intergenic_2,0,0,0,0,0,0,1,3,0,4,0,0,0,0,0,1
intergenic_3,0,0,0,0,0,0,0,0,0,1,0,2,0,3,0,0
intergenic_4,0,0,0,3,0,0,0,10,0,1,1,1,0,0,2,0
intergenic_5,0,0,0,0,1,1,1,5,2,8,1,4,0,1,0,1
intergenic_6,0,0,0,5,0,0,0,6,0,4,6,3,3,0,0,1
intergenic_7,1,3,2,9,1,12,3,7,2,11,6,8,6,3,12,4
intergenic_8,0,5,6,1,1,8,4,15,3,4,7,6,4,0,0,2

To read in the intergenic read counts, type

```
>intergenic<-read.table("L:/Lab/example/readCounts_intergenic.txt",header=TRUE,sep=",")
```

(c) Merge the genic and intergenic read counts together

In order to calculate the low-expression threshold, the genic and intergenic read counts must be merged together. This is accomplished by typing

```
>HTSeqwIR<-rbind(genic,intergenic)
```

Note that when you use rbind to merge two data frames, the headers of two data frames must be the same.

If you type in

```
>HTSeqwIR
```

R will output the merged data set to the terminal as follows:

```
  variable_name biosample1  biosample2  biosample3  biosample4  biosample5
1      gene_1       0       0       0       0       0
2      gene_2      92      161      76      70      140
3      gene_3       5       1       0       0       4
4      gene_4       5      36       4       1      15
5      gene_5     4664    8714    3564    3150    6205
6      gene_6     583     761     245     310     722
7  intergenic_1     1       7       3       0       1
8  intergenic_2     0       0       0       0       0
9  intergenic_3     0       0       0       0       0
10 intergenic_4     0       0       0       3       0
11 intergenic_5     0       0       0       0       1
12 intergenic_6     0       0       0       5       0
13 intergenic_7     1       3       2       9       1
14 intergenic_8     0       5       6       1       1
  biosample6  biosample7  biosample8  biosample9  biosample10  biosample11
1      0       1       2       0       0       0
2     88      70      90       8      218       4
3      0       0       7       0       2       6
```

4	10	7	19	3	4	1
5	3072	3334	4300	253	459	1211
6	299	308	437	111	450	32
7	2	1	2	2	8	4
8	0	1	3	0	4	0
9	0	0	0	0	1	0
10	0	0	10	0	1	1
11	1	1	5	2	8	1
12	0	0	6	0	4	6
13	12	3	7	2	11	6
14	8	4	15	3	4	7

	biosample12	biosample13	biosample14	biosample15	biosample16
1	0	0	2	0	3
2	10	76	23	0	103
3	21	5	17	2	8
4	0	4	13	4	0
5	1452	542	482	1568	244
6	29	158	80	194	269
7	2	5	1	2	1
8	0	0	0	0	1
9	2	0	3	0	0
10	1	0	0	2	0
11	4	0	1	0	1
12	3	3	0	0	1
13	8	6	3	12	4

```
14        6        4        0        0        2
```

You may want to check the dimensions of the merged file with the dim command:

```
>dim(HTSeqwIR)
```

```
[1] 14  17
```

Here the first column includes the genes and intergenic region IDs.

(d) Read in the background information of biosample

biosample_summary.txt is as follows:

biosample_ID	genotype	sex	environment
biosample1	L1	F	1
biosample2	L1	F	1
biosample3	L1	M	1
biosample4	L1	M	1
biosample5	L2	F	1
biosample6	L2	F	1
biosample7	L2	M	1
biosample8	L2	M	1
biosample9	L1	F	2
biosample10	L1	F	2
biosample11	L1	M	2
biosample12	L1	M	2
biosample13	L2	F	2
biosample14	L2	F	2
biosample15	L2	M	2
biosample16	L2	M	2

Read in the file and define genotype, sex, and environment as factors.

```
>bioCond<-read.table("L:/Lab/example/biosample_summary.txt",header=TRUE,sep="\t")
>dim(bioCond)
```

```
[1] 16 4
```

```
>bioCond[,'genotype']<-as.factor(bioCond[,'genotype'])
>bioCond[,'sex']<-as.factor(bioCond[,'sex'])
>bioCond[,'environment']<-as.factor(bioCond[,'environment'])
```

(e) Identify the low-expression threshold (LET) using 95% upper-quartile of log2(normalized_intergenic_read_counts +1)

First, the read count data need to be normalized using DESeq normalization. If there are any genes or intergenic regions that have zero read counts for all samples, they will be removed.

```
>library("DESeq")
```

This step creates a file (named “T” in this example). It removes the genes and intergenic regions that have zero read counts across all samples. It also removes the first column of HTSeqwIR, which contains the gene and intergenic IDs.

```
>rc<- rowSums(HTSeqwIR[,-1])
>T<-HTSeqwIR[rc>0,-1]
>rownames(T)<- HTSeqwIR[rc>0,1]
>dim(T)

[1]  14 16
```

The experimental conditions are then added to the raw count data set, and the normalization parameters are calculated using estimateSizeFactors. The read counts are then normalized in the DESeqwIR data frame, and the genic and intergenic IDs are added.

```
>cds=newCountDataSet(T,bioCond)
>cds=estimateSizeFactors(cds)
>DESeqwIR<-counts(cds,normalized=TRUE)
>rownames(DESeqwIR)<-HTSeqwIR[rc>0,1]
```

The low-expression threshold (LET) is identified using the 95% upper quartile of log2(normalized intergenic read counts + 1):

```
>LET<-quantile(log2(DESeqwIR[grep("intergenic",rownames(DESeqwIR)),]+1),0.95)
>LET

      95%
 3.184019
```

VII. Performing the RNA-Seq data analysis using the DESeq R package

(a) Read in the gene read counts and background conditions

Commands:

```
>genic<-read.table("L:/Lab/example/readCounts_genic.txt",header=TRUE,sep=",")
>dim(genic)

[1] 6  17

>bioCond<-read.table("L:/Lab/example/biosample_summary.txt",header=TRUE,sep="\t")
>dim(bioCond)
```

```
[1]  16  4
```

Define genotype, sex and environment as factors:

```
>bioCond[,'genotype']<-as.factor(bioCond[,'genotype'])
>bioCond[,'sex']<-as.factor(bioCond[,'sex'])
>bioCond[,'environment']<-as.factor(bioCond[,'environment'])
```

(b) If desired, identify the genes that fail to pass the low-expression threshold cutoff value and remove them as in Workflow 1. This provides you with a file having the normalized read counts for each gene that are greater than the low-expression threshold; this data can be used for plotting or further analysis.

Use the data frame having genic raw read counts only and eliminate any gene that has zero read counts in all samples:

```
>rc<-rowSums(genic[,-1])
>N <- colSums(genic[,-1])
>T<-genic[rc>0,-1]
>rownames(T)<-genic[rc>0,1]
>dim(T)
```

```
[1] 6  16
```

Normalize the raw read counts using the DESeq normalization method:

```
>Design<-bioCond[,2:4]
>cds=newCountDataSet(T,Design)
>cds=estimateSizeFactors(cds)
>DESeq<-counts(cds,normalized=TRUE)
>dim(DESeq)
```

```
[1] 6   16
```

```
>oLET<-2^LET-1
```

Note that oLET is for the normalized read counts, not for the raw read counts.

```
>indNSC_DESeq<-which(apply(DESeq,1,function(x){all(x < oLET)})==TRUE)
>length(indNSC_DESeq)
```

```
[1] 1
```

```
>keep_DESeq<-rep(TRUE,nrow(DESeq))
>keep_DESeq[indNSC_DESeq]<-FALSE
```

The following data frame is the normalized read counts according to workflow 1. First, the data are normalized; then it is filtered.

```
>DESeq_w1<-DESeq[keep_DESeq,]
```

(c) Fit the GLM and test hypotheses

```
>rc<-rowSums(genic[,-1])
>N <- colSums(genic[,-1])
>T<-genic[rc>0,-1]
>rownames(T)<-genic[rc>0,1]
>dim(T)
```

```
[1] 6   16
```

Normalize the data using the DESeq normalization method. If you wish to use another normalization method, you can provide the size factor (i.e., normalization factor) using the command "pData(cdsFull)$sizeFactor<-TCSFactor". Please refer to section VIII for instructions to calculate the size factor if other normalization methods are desired.

```
>Design<-bioCond[,2:4]
>cdsFull=newCountDataSet(T,Design)
>cdsFull=estimateSizeFactors(cdsFull)
>cdsFull=estimateDispersions(cdsFull,fitType="local")
```

Fit the model and test hypotheses

For a multi-factor design, if you are interested in testing each main effect and interaction term (in multi-factor case), you need to fit the full and reduced GLMs, then perform the chi-squared test comparing the full and reduced GLMs.

Examples:
(i) Hypothesis testing of the main effect: genotype.

The full and reduced GLM fits are performed using fitNbinomGLMs().

The full model is

```
>fit1<-fitNbinomGLMs(cdsFull,count~genotype+sex+environment)
```

The reduced model is

```
>fit1G<-fitNbinomGLMs(cdsFull,count~sex+environment)
```

Next, nbinomGLMTest() is used to test hypotheses and report *P*-values

```
>mod_G<-nbinomGLMTest(fit1,fit1G)
```

Calculate the False Discovery Rate (FDR) values for each *P*-value.

```
>fdr_G<-p.adjust(mod_G,method="BH")
```

Save the results in a data frame ResDE_G with initial values 0 and dimension length(mod_G)x2. The first column is the gene; the second column is the *P*-value; and the third column is the FDR.

```
>ResDE_G<-data.frame(matrix(0,length(mod_G),2))

>ResDE_G[,1]<-mod_G
>ResDE_G[,2]<-fdr_G

>rownames(ResDE_G)<-genic[rc>0,1]
>colnames(ResDE_G)<-c("P-values","FDR")

>write.table(ResDE_G,"L:/Lab/example/ResDE_G.txt",quote=FALSE,col.names=TRUE,row.names=TRUE,sep="\t")

>ResDE_G

          P-values        FDR
gene_1 0.033559799   0.10067940
gene_2 0.751308704   0.75130870
gene_3 0.643374383   0.75130870
gene_4 0.173092060  0.34618412
gene_5 0.007815565  0.04689339
gene_6 0.467931626  0.70189744
```

To remove the genes that do not pass the low-expression threshold, as in Workflow 1, and save the results:

```
>modw1_G<-mod_G[keep_DESeq]
>fdrw1_G<-p.adjust(modw1_G,method="BH")
>ResDEw1_G<-data.frame(matrix(0,length(modw1_G),2))
>ResDEw1_G [,1]<-modw1_G
>ResDEw1_G [,2]<-fdrw1_G

>rownames(ResDEw1_G)<-genic[rc>0,1][keep_DESeq]
>colnames(ResDEw1_G)<-c("P-values","FDR")

>write.table(ResDEw1_G,"L:/Lab/example/ResDEw1_G.txt",quote=FALSE,col.names=TRUE,row.names=TRUE,sep="\t")

>ResDEw1_G


          P-values          FDR
gene_2   0.751308704   0.75130870
```

gene_3	0.643374383	0.75130870
gene_4	0.173092060	0.43273015
gene_5	0.007815565	0.03907782
gene_6	0.467931626	0.75130870

(ii) Hypothesis testing of two-way interaction term: genotype × environment

The full model is:

```
>fit2GE<-fitNbinomGLMs(cdsFull,count~genotype+sex+environment + genotype:environment)
```

The reduced model is:

```
>fit1<-fitNbinomGLMs(cdsFull,count~genotype+sex+environment)
```

Next, nbinomGLMTest() is used to test hypotheses and report *P*-values

```
>mod_GE<-nbinomGLMTest(fit2GE,fit1)
```

Calculate the False Discovery Rate (FDR) values for each *P*-value.

```
>fdr_GE<-p.adjust(mod_GE,method="BH")
```

Save the results in a data frame ResDE_GE with initial values 0 and dimension length(mod_GE)x2. The first column is the gene; the second column is the *P*-value; and the third column is the FDR.

```
>ResDE_GE<-data.frame(matrix(0,length(mod_GE),2))

>ResDE_GE[,1]<-mod_GE
>ResDE_GE[,2]<-fdr_GE

>rownames(ResDE_GE)<-genic[rc>0,1]
>colnames(ResDE_GE)<-c("P-values","FDR")

>write.table(ResDE_GE,"L:/Lab/example/ResDE_GE.txt",quote=FALSE,col.names=TRUE,row.names=TRU
E,sep="\t")

>ResDE_GE
```

	P-values	FDR
gene_1	0.99997682	0.9999768
gene_2	0.78750456	0.9999768
gene_3	0.78413333	0.9999768
gene_4	0.99550735	0.9999768
gene_5	0.01751661	0.1050997
gene_6	0.56990751	0.9999768

To remove the genes that do not pass the low-expression threshold, as in Workflow 1, and save the results:

```
>modw1_GE<-mod_GE[keep_DESeq]
>fdrw1_GE<-p.adjust(modw1_GE,method="BH")
>ResDEw1_GE<-data.frame(matrix(0,length(modw1_GE),2))
>ResDEw1_GE[,1]<-modw1_GE
>ResDEw1_GE[,2]<-fdrw1_GE

>rownames(ResDEw1_GE)<-genic[rc>0,1][keep_DESeq]
>colnames(ResDEw1_GE)<-c("P-values","FDR")

>write.table(ResDEw1_GE,"L:/Lab/example/ResDEw1_GE.txt",quote=FALSE,col.names=TRUE,row.names
=TRUE,sep="\t")

>ResDEw1_GE
```

	P-values	FDR
gene_2	0.78750456	0.98438070
gene_3	0.78413333	0.98438070
gene_4	0.99550735	0.99550735
gene_5	0.01751661	0.08758307
gene_6	0.56990751	0.98438070

(iii) Hypothesis testing of three-way interaction term: genotype x environment x sex

The full model is:

```
>fit3<-fitNbinomGLMs(cdsFull,count~genotype+sex+environment +
genotype:environment+genotype:sex+environment:sex+genotype:environment:sex)
```

The reduced model is:

```
>fit2<-fitNbinomGLMs(cdsFull,count~genotype+sex+environment +
genotype:environment+genotype:sex+environment:sex)
```

Next, nbinomGLMTest() is used to test hypotheses and report *P*-values

```
>mod_GES<-nbinomGLMTest(fit3,fit2)
```

Calculate the False Discovery Rate (FDR) values for each *P*-value.

```
>fdr_GES<-p.adjust(mod_GES,method="BH")
```

Save the results in a data frame ResDE with initial values 0 and dimension length(mod_GES)x2. The first column is the gene; the second column is the *P*-value; and the third column is the FDR.

```
>ResDE_GES<-data.frame(matrix(0,length(mod_GES),2))
```

```
>ResDE_GES[,1]<-mod_GES
>ResDE_GES[,2]<-fdr_GES

>rownames(ResDE_GES)<-genic[rc>0,1]
>colnames(ResDE_GES)<-c("P-values","FDR")

>write.table(ResDE_GES,"L:/Lab/example/ResDE_GES.txt",quote=FALSE,col.names=TRUE,row.names=TRUE,sep="\t")

>ResDE_GES
```

	P-values	FDR
gene_1	1.000000e+00	1.000000e+00
gene_2	3.712282e-01	5.536799e-01
gene_3	1.397979e-02	4.193936e-02
gene_4	4.613999e-01	5.536799e-01
gene_5	1.306267e-05	7.837603e-05
gene_6	3.186831e-02	6.373662e-02

To remove the genes that do not pass the low-expression threshold, as in Workflow 1, and save the results:

```
>modw1_GES<-mod_GES[keep_DESeq]
>fdrw1_GES<-p.adjust(modw1_GES,method="BH")
>ResDEw1_GES<-data.frame(matrix(0,length(modw1_GES),2))
>ResDEw1_GES[,1]<-modw1_GES
>ResDEw1_GES[,2]<-fdrw1_GES

>rownames(ResDEw1_GES)<-genic[rc>0,1][keep_DESeq]
>colnames(ResDEw1_GES)<-c("P-values","FDR")

>write.table(ResDEw1_GES,"L:/Lab/example/ResDEw1_GES.txt",quote=FALSE,col.names=TRUE,row.names=TRUE,sep="\t")

>ResDEw1_GES
```

	P-values	FDR
gene_2	3.712282e-01	4.613999e-01
gene_3	1.397979e-02	3.494947e-02
gene_4	4.613999e-01	4.613999e-01
gene_5	1.306267e-05	6.531336e-05
gene_6	3.186831e-02	5.311385e-02

VIII. Implementation of RNA-Seq read counts normalization methods in R (if desired by the user)

Preliminary calculations using the raw read counts for each gene:

```
>rc<-rowSums(genic[,-1])
```

```
>N <- colSums(genic[,-1])

>T<-genic[rc>0,-1]
>dim(T)

[1] 6    16
```

Total Counts (TC) normalization:

```
>N <- colSums(T)
>TCSFactor<-N/mean(N)
>TCSeq<-scale(T,center=FALSE,scale=TCSFactor)
```

Upper Quartile (UQ) normalization:

```
>UQSFactor<-apply(T,2,quantile,0.75)
>UQSFactor<-UQSFactor/mean(UQSFactor)
>UQSeq<-scale(T,center=FALSE,scale=UQSFactor)
```

Median (M) normalization:

```
>MedSFactor<-apply(T,2,median)
>MedSFactor<-MedSFactor/mean(MedSFactor)
>MedSeq<-scale(T,center=FALSE,scale=MedSFactor)
```

Quantile (Q) normalization:

Note that you may need to install the limma package from Bioconductor before proceeding.

```
>library(limma)
>QSeq<-normalizeQuantiles(T)
```

Trimmed Mean of M-values (TMM) normalization:

```
>library(edgeR)
>N <- colSums(T)
>f <- calcNormFactors(as.matrix(T),method="TMM")
>f.scale <- N*f / mean(N*f)
>TMMSeq<-scale(T,center=FALSE,scale=f.scale)
```

DESeq (DESeq) normalization:

```
>library(DESeq)
>cds=newCountDataSet(T,condition=bioCond)
>cds=estimateSizeFactors(cds)
```

```
>DESeq<-counts(cds,normalized=TRUE)
```

RPKM normalization:

First, provide the length of each gene in the read count data set.

```
>trsLength<-c(299,35867,22836,45027,32017,1851)

>RPKMSeq <- apply(T,2,function(s){
 lengthsInKb <- trsLength/1000
 millionMapped <- sum(s)/1e+06
 rpm <- s/millionMapped
 rpkm <- rpm/lengthsInKb
})
```

IX. Estimate dispersion using the DESeq R package (if desired by the user)

(a) Read in the data in the format required by DESeq.

The first column of genic, which contains the gene ID numbers, needs to be removed using “-1”.

```
>rc<-rowSums(genic[,-1])
>N <- colSums(genic[,-1])
```

Use the rc command to choose the genes that have at least one sample with nonzero read counts.

```
>T<-genic[rc>0,-1]
>dim(T)
```

```
[1] 6     16
```

Normalize the data using DESeq normalization:

```
>Design<-bioCond[,2:4]
>cdsFull=newCountDataSet(T,Design)
>cdsFull=estimateSizeFactors(cdsFull)
```

(b) Estimate the dispersion for hypothesis testing

(i) If there are no replicates in each condition, you will use the following command to estimate the dispersion

```
>cdsFull=estimateDispersions(cdsFull,fitType="local",method="blind",sharingMode="fit-only")
```

(ii) If there are replicates in each condition or replicates in partial conditions, you will use the following command to estimate the dispersion

```
>cdsFull=estimateDispersions(cdsFull,fitType="local")
```

Note: use help(estimateDispersions) for more details about other options for method, fitType and sharingMode.

(c) Estimate dispersion per gene separately for each analysis factor or combination of factors (if desired by the user). Here we provide three examples, which show how to estimate the dispersion among different factors in the model.

(i) Example: Find the dispersion among genotypes.

```
>cdsFullG=newCountDataSet(T,condition=bioCond[,2])
>cdsFullG=estimateSizeFactors(cdsFullG)
>cdsFullG=estimateDispersions(cdsFullG,method="per-condition",fitType="local")

>perGeneDispG<-matrix(0,nrow(T),length(levels(bioCond[,2])))

>for (i in 1:2){
  tt<-get(levels(bioCond[,2])[i], envir=cdsFullG@fitInfo)
  perGeneDispG[,i]<-tt$perGeneDispEsts

}

>colnames(perGeneDispG)<-levels(bioCond[,2])
>rownames(perGeneDispG)<-genic[rc>0,1]

>perGeneDispG
```

Each column has one genotype.

	L1	L2
gene_1	NaN	1.90459354
gene_2	1.9201511	0.94839945
gene_3	3.9902526	2.53925080
gene_4	0.4895308	1.63669785
gene_5	0.3320107	0.09747364
gene_6	0.5171646	0.30754295

Note that the ‘NaN’ in the above output occurs because all read counts for gene_name1 for L1 are zero.

(ii) Example: Find the dispersion among sexes.

```
>cdsFullS=newCountDataSet(T,condition=bioCond[,3])
>cdsFullS=estimateSizeFactors(cdsFullS)
>cdsFullS=estimateDispersions(cdsFullS,method="per-condition",fitType="local")
```

```
>perGeneDispS<-matrix(0,nrow(T),length(levels(bioCond[,3])))
>for (i in 1:2){
  tt<-get(levels(bioCond[,3])[i], envir=cdsFullS@fitInfo)
  perGeneDispS[,i]<-tt$perGeneDispEsts

}

>colnames(perGeneDispS)<-levels(bioCond[,3])
>rownames(perGeneDispS)<-genic[rc>0,1]

>perGeneDispS
```

Each column is for either sex.

	F	M
gene_1	6.1160571	3.8797993
gene_2	1.0278695	1.7775499
gene_3	4.2500223	2.5473077
gene_4	1.1691711	0.4439577
gene_5	0.1260737	0.2592768
gene_6	0.2349392	0.6213733

(iii) Example: Find the dispersion among environment conditions.

```
>cdsFullE=newCountDataSet(T,condition=bioCond[,4])
>cdsFullE=estimateSizeFactors(cdsFullE)
>cdsFullE=estimateDispersions(cdsFullE,method="per-condition",fitType="local")



>perGeneDispE<-matrix(0,nrow(T),length(levels(bioCond[,4])))
>for (i in 1:2){
  tt<-get(levels(bioCond[,4])[i], envir=cdsFullE@fitInfo)
  perGeneDispE[,i]<-tt$perGeneDispEsts

}

>colnames(perGeneDispE)<-levels(bioCond[,4])
>rownames(perGeneDispE)<-genic[rc>0,1]

>perGeneDispE
```

Each column is for each environment condition.

	1	2
gene_1	1.019417303	1.9733691
gene_2	0.014635991	1.1438251
gene_3	1.536922671	1.1686239

gene_4	0.304140038	1.8480639
gene_5	0.008413909	0.6184193
gene_6	0.005427313	0.4028957

(iv) dispersion of each condition of interaction term: genotype x environment

```
>cdsFullGE=newCountDataSet(T,condition=as.factor(paste(bioCond[,2],bioCond[,4],sep='')))
>cdsFullGE=estimateSizeFactors(cdsFullGE)
>cdsFullGE=estimateDispersions(cdsFullGE,method="per-condition",fitType="local")


>GE<-levels(as.factor(paste(bioCond[,2],bioCond[,4],sep='')))


>perGeneDispGE<-matrix(0,nrow(T),length(GE))

>for (i in 1:length(GE)){
  tt<-get(GE[i], envir=cdsFullGE@fitInfo)
  perGeneDispGE[,i]<-tt$perGeneDispEsts

}

> colnames(perGeneDispGE)<- levels(as.factor(paste(bioCond[,2],"E",bioCond[,4],sep='')))

>rownames(perGeneDispGE)<-genic[rc>0,1]

>perGeneDispGE
```

Each column is for one genotype/environment condition.

	L1E1	L1E2	L2E1	L2E2
gene_1	NaN	NaN	1.112050e-01	0.6965195
gene_2	0.01517909	2.1614642	1.824153e-02	0.7829998
gene_3	2.02696670	1.7697790	1.435044e+00	1.0033168
gene_4	0.92983789	0.3337691	3.348200e-02	1.7510700
gene_5	0.01522943	0.6272304	2.115951e-03	0.1634978
gene_6	0.01294851	0.6995052	-2.684236e-05	0.2879320

Note that DESeq assumes the read counts follows a negative binomial distribution with mean μ and dispersion a such that variance v= μ+ a μ^2. Then it uses a method-of-moments estimator to estimate the dispersion a, i.e., a=(v- μ)/ μ^2. For large μ, it may be that the estimate for v is smaller than that for μ, which leads to the negative estimate value for the dispersion a. DESeq will automatically replace all negative values with small positive ones before any statistical test is performed.

(v) dispersion of each condition of three-way interaction term: genotype x environment x sex

```
>cdsFullGES=newCountDataSet(T,condition=as.factor(paste(bioCond[,2],
bioCond[,3],bioCond[,4],sep='')))
>cdsFullGES=estimateSizeFactors(cdsFullGES)
>cdsFullGES=estimateDispersions(cdsFullGES,method="per-condition",fitType="local")


>GES<-levels(as.factor(paste(bioCond[,2], bioCond[,3],bioCond[,4],sep='')))


>perGeneDispGES<-matrix(0,nrow(T),length(GES))

>for (i in 1:length(GES)){
  tt<-get(GES[i], envir=cdsFullGES@fitInfo)
  perGeneDispGES[,i]<-tt$perGeneDispEsts

}


>colnames(perGeneDispGES)<- levels(as.factor(paste(bioCond[,2],bioCond[,3],bioCond[,4],sep='')))

>rownames(perGeneDispGES)<-genic[rc>0,1]

>perGeneDispGES
```

Each column is for one genotype/environment/sex condition.

	L1F1	L1F2	L1M1	L1M2	L2F1	L2F2	L2M1	L2M2
gene_1	NaN	NaN	NaN	NaN	NaN	1.16448624	-0.627199551	1.5118293
gene_2	-0.001939391	1.32593825	-0.004169841	0.194478196	0.043245209	0.26373409	-0.011447787	1.9857814
gene_3	0.904055215	0.14399636	NaN	0.510499103	1.197880227	0.81661772	1.663893192	1.0902229
gene_4	0.769770491	-0.07357025	0.369441223	0.040677228	-0.013522503	0.75867149	0.141505838	1.2118010
gene_5	0.015829383	0.07544373	0.015708449	0.009011146	0.003732940	0.03716155	0.000869951	0.4947524
gene_6	0.014456912	0.07374273	0.012390383	-0.022989851	0.001658889	0.03015452	-0.001619112	0.4921805

X. Performing the RNA-Seq data analysis using edgeR R package (if desired by the user)

(a) Read in data in the format required by edgeR

```
>library(edgeR)
```

```
>rc<-rowSums(genic[,-1])
>N <- colSums(genic[,-1])
>T<-genic[rc>0,-1]
>rownames(T)<-genic[rc>0,1]
>colnames(T)<-colnames(genic[,-1])

>Design<-data.frame(bioCond[,c(2:4)])
>genotype<-as.factor(Design$genotype)
>sex<-as.factor(Design$sex)
>environment<-as.factor(Design$environment)


>Group<-factor(paste(Design$genotype,Design$sex,Design$environment,sep="."))
>y<-DGEList(counts=T,group=Group)
```

(b) Normalize data and estimate dispersion

```
>y<-calcNormFactors(y,method="RLE")
```

Note that RLE stands for “relative log expression” and implements the method used by Anders and Huber in the DESeq package. Type help(calcNormFactors) for additional detail.

Note that other normalization methods are also available in this function, such as the TMM normalization command: y<-calcNormFactors(y,method="TMM") or the Upper Quartile normalization: y<-calcNormFactors(y,method="upperquartile").

```
>y<-estimateCommonDisp(y)
>y<- estimateTagwiseDisp(y)
```

(c) Fit the GLM and test hypotheses

(i) Test the interaction term: genotype x environment

```
>design<-model.matrix(~genotype+sex+environment+genotype:environment)
>fite_GE<-glmFit(y,design)
>colnames(fite_GE)

[1] "(Intercept)"          "genotypeL2"          "sexM"               "environment2"
[5] "genotypeL2:environment2"
```

Based on this output, you will be able to choose the correct coefficients used in the next command, such as "coef=5" in testing the interaction term genotype x environment:

```
>lrte_GE<-glmLRT(fite_GE,coef=5)
>head(lrte_GE$table)
```

	logFC	logCPM	LR	PValue
gene_1	2.8190517	9.920413	-9.109836e-09	1.00000000
gene_2	0.7554257	14.976983	2.361810e-01	0.62697811
gene_3	-0.3998179	12.147280	5.707176e-02	0.81118548
gene_4	0.4147551	11.883808	8.445810e-02	0.77134444
gene_5	-0.3245378	19.768691	2.876729e-01	0.59171568
gene_6	0.4960305	16.846218	2.923919e+00	0.08727564

Based on this output, you will know which column reports the *P*-values for testing the genotype x environment effect.

```
>length(which(p.adjust((lrte_GE$table)[,4],method="BH")<0.05))
```

```
[1] 0
```

(ii) Test the main effects of genotype, environment and Sex

```
>design2<-model.matrix(~genotype+environment +sex)
>fite2<-glmFit(y,design2)
>colnames(fite2)
```

```
[1] "(Intercept)"  "genotypeL2"   "environment2" "sexM"
```

```
>lrte_G <- glmLRT(fite2,coef=2)
>lrte_E <- glmLRT(fite2,coef=3)
>lrte_S <- glmLRT(fite2,coef=4)
```

(a) *P*-values for genotype effect:

```
>head(lrte_G$table)
```

	logFC	logCPM	LR	PValue
gene_1	3.2125076	9.920413	6.7794082	0.009221546
gene_2	0.4298146	14.976983	0.3125905	0.576094885
gene_3	0.6449546	12.147280	0.5902954	0.442304906
gene_4	1.3961964	11.883808	4.3050309	0.037999805
gene_5	-0.2005686	19.768691	0.4367117	0.508713886
gene_6	0.3142677	16.846218	4.6680041	0.030729620

Based on this output, you will know which column reports the *P*-values for testing genotype effect.

```
>length(which(p.adjust((lrte_G$table)[,4],method="BH")<0.05))
```

```
[1] 0
```

Result: none gene is reported to be significant for genotype effect based on fdr less than 0.05.

(b) *P*-values for environment effect:

```
>head(lrte_E$table)
            logFC     logCPM         LR       PValue
gene_1  2.1299920   9.920413  2.7542708 9.699503e-02
gene_2  0.6807991  14.976983  0.7345931 3.913984e-01
gene_3  4.1902207  12.147280 20.8813508 4.886287e-06
gene_4  0.2194118  11.883808  0.0909979 7.629123e-01
gene_5 -0.5252454  19.768691  3.0525553 8.061022e-02
gene_6  0.3880328  16.846218  7.2043567 7.272681e-03
```

Based on this output, you will know which column reports the *P*-values for testing environment effect.

```
>length(which(p.adjust((lrte_E$table)[,4],method="BH")<0.05))

[1] 2
```

Result: there are two genes reported to be significant for environment effect based on fdr less than 0.05.

(c) *P*-values for sex effect:

```
>head(lrte_S$table)

            logFC     logCPM         LR       PValue
gene_1  0.6566719   9.920413  0.5235772  0.469319717
gene_2 -0.6590087  14.976983  0.6994613  0.402964767
gene_3  0.2296472  12.147280  0.0734507  0.786377269
gene_4 -1.6235918  11.883808  5.6013517  0.017946626
gene_5  0.5370514  19.768691  3.0627478  0.080106098
gene_6 -0.4888427  16.846218 11.2527975  0.000795031
```

Based on this output, you are able to know which column reports the *P*-values for testing sex effect.

```
>length(which(p.adjust((lrte_S$table)[,4],method="BH")<0.05))

[1] 1
```

Result: there is one gene reported to be significant for sex effect based on fdr less than 0.05.

(d) Save the results including *P*-values and FDR values

If you choose not to filter the data for low-expression genes, then

```
>ReseR<-data.frame(matrix(0,nrow(y),8))
>ReseR[,1]<-(lrte_G$table)[,4]
>ReseR[,2]<-p.adjust((lrte_G$table)[,4],method="BH")
>ReseR[,3]<-(lrte_E$table)[,4]
>ReseR[,4]<-p.adjust((lrte_E$table)[,4],method="BH")
>ReseR[,5]<-(lrte_S$table)[,4]
```

```
>ReseR[,6]<-p.adjust((lrte_S$table)[,4],method="BH")
>ReseR[,7]<-(lrte_GE$table)[,4]
>ReseR[,8]<-p.adjust((lrte_GE$table)[,4],method="BH")

>colnames(ReseR)<-
c("Pvalue_G","FDR_G","Pvalue_E","FDR_E","Pvalue_S","FDR_S","Pvalue_GE","FDR_GE")
>rownames(ReseR)<-genic[rc>0,1]

>write.table(ReseR,"L:/Lab/example/ReseR.txt",quote=FALSE,col.names=TRUE,row.names=TRUE,sep="\
t")

>ReseR
```

```
            Pvalue_G        FDR_G           Pvalue_E        FDR_E           Pvalue_S
FDR_S             Pvalue_GE         FDR_GE

gene_1     0.009221546     0.05532928     9.699503e-02     1.454925e-01     0.469319717
0.563183660     1.00000000          1.0000000

gene_2     0.576094885     0.57609488     3.913984e-01     4.696781e-01     0.402964767
0.563183660     0.62697811          0.9734226

gene_3     0.442304906     0.57609488     4.886287e-06     2.931772e-05     0.786377269
0.786377269     0.81118548          0.9734226

gene_4     0.037999805     0.07599961     7.629123e-01     7.629123e-01     0.017946626
0.053839878     0.77134444          0.9734226

gene_5     0.508713886     0.57609488     8.061022e-02     1.454925e-01     0.080106098
0.160212196     0.59171568          0.9734226

gene_6     0.030729620     0.07599961     7.272681e-03     2.181804e-02     0.000795031
0.004770186     0.08727564          0.5236538
```

If you choose to follow Workflow 1 from the paper, which is normalization and dispersion estimation first, followed by the filtering out of low-expression genes, then

```
>y1<-y[keep_DESeq,]

>ReseRw1<-data.frame(matrix(0,nrow(y1),8))
>ReseRw1[,1]<-(lrte_G$table)[,4][keep_DESeq]
>ReseRw1[,2]<-p.adjust(ReseRw1[,1],method="BH")
>ReseRw1[,3]<-(lrte_E$table)[,4][keep_DESeq]
>ReseRw1[,4]<-p.adjust(ReseRw1[,3],method="BH")

>ReseRw1[,5]<-(lrte_S$table)[,4][keep_DESeq]
>ReseRw1[,6]<-p.adjust(ReseRw1[,5],method="BH")
>ReseRw1[,7]<-(lrte_GE$table)[,4][keep_DESeq]
```

```
>ReseRw1[,8]<-p.adjust(ReseRw1[,7],method="BH")

>colnames(ReseRw1)<-
c("Pvalue_G","FDR_G","Pvalue_E","FDR_E","Pvalue_S","FDR_S","Pvalue_GE","FDR_GE")
>rownames(ReseRw1)<-genic[rc>0,1][keep_DESeq]

>write.table(ReseRw1,"L:/Lab/example/ReseRw1.txt",quote=FALSE,col.names=TRUE,row.names=TRUE,s
ep="\t")

ReseRw1
```

	Pvalue_G	FDR_G	Pvalue_E	FDR_E	Pvalue_S	FDR_S	Pvalue_GE	FDR_GE
gene_2	0.57609488	0.57609488	3.913984e-01	4.892480e-01	0.402964767	0.503705959	0.62697811	0.8111855
gene_3	0.44230491	0.57609488	4.886287e-06	2.443144e-05	0.786377269	0.786377269	0.81118548	0.8111855
gene_4	0.03799981	0.09499951	7.629123e-01	7.629123e-01	0.017946626	0.044866565	0.77134444	0.8111855
gene_5	0.50871389	0.57609488	8.061022e-02	1.343504e-01	0.080106098	0.133510164	0.59171568	0.8111855
gene_6	0.03072962	0.09499951	7.272681e-03	1.818170e-02	0.000795031	0.003975155	0.08727564	0.4363782

Acknowledgements: We thank M.-A. Dillies for R normalization code.

References

1. Anders S and Huber W. Differential expression analysis for sequence count data. *Genome Biol*. 2010;doi:10.1186/gb-2010-11-10-r106.

2. Robinson MD, McCarthy DJ and Smyth GK. EdgeR: a Bioconductor package for differential expression analysis of digital gene expression data. *Bioinformatics.* 2010:139-140.

3. Ritchie RE, Phipson B, Wu D, Hu Y, Law CW, Shi W, Smyth GK. *limma* powers differential expression analyses for RNA-sequencing and microarray studies. *Nucl Acids Res* 43:e47.
